# What do patients and the public know about clinical practice guidelines and what do they want from them? A qualitative study

**DOI:** 10.1186/s12913-016-1319-4

**Published:** 2016-02-24

**Authors:** Naomi Fearns, Joanna Kelly, Margaret Callaghan, Karen Graham, Kirsty Loudon, Robin Harbour, Nancy Santesso, Emma McFarlane, Judith Thornton, Shaun Treweek

**Affiliations:** Healthcare Improvement Scotland, Gyle Square, 1 South Gyle Crescent, Edinburgh, EH12 9EB UK; University of Stirling, Scotland, UK; Department of Clinical Epidemiology and Biostatistics, McMaster University, 1280 Main Street West, Hamilton, Ontario L8S4L8 Canada; National Institute for Health and Care Excellence, England, UK; Health Services Research Unit, University of Aberdeen, Scotland, UK

**Keywords:** Guidelines, Clinical practice guideline, Patient guidelines, Patient version

## Abstract

**Background:**

Guideline producers are increasingly producing versions of guidelines for the public. The aim of this study was to explore what patients and the public understand about the purpose and production of clinical guidelines, and what they want from clinical guidelines to support their healthcare decisions.

**Methods:**

Participants were purposively selected to represent a range of the likely users of patient versions of guidelines, including individuals with health conditions (diabetes and depression), general members of the public, health communication professionals and a group of young people. Participants were asked about their awareness and understanding of clinical guidelines and presented with scenario recommendations, or draft materials from patient guidelines to prompt discussion. Each discussion was facilitated by one or two researchers. All focus groups were recorded and transcribed prior to analysis. Data were analysed using framework analysis.

**Results:**

We ran nine focus groups involving 62 individuals, supplemented by four interviews with people experiencing homelessness. Eight groups were held in Scotland, one in England. The four interviews were held in Scotland. The framework analysis yielded five themes: access and awareness; what patients want to know; properties of guidelines; presenting evidence; and format. Awareness of guidelines was low. Participants emphasised the need for information that enables them to choose between treatment options, including harms. They would like help with this from healthcare professionals, especially general practitioners. Participants differed in their support for the inclusion of numerical information and graphs.

**Conclusions:**

Members of the public want information to help them choose between treatments, including information on harms, particularly to support shared decisions with health professionals. Presenting numerical information is a challenge and layered approaches that present information in stages may be helpful. Ignoring the themes identified in this study is likely to lead to materials that fail to support public and patient healthcare decision making.

**Electronic supplementary material:**

The online version of this article (doi:10.1186/s12913-016-1319-4) contains supplementary material, which is available to authorized users.

## Background

Clinical practice guidelines and health technology assessments have emerged as a source of support to individuals wanting to use research evidence in their healthcare decisions. The science and methodology behind clinical practice guidelines has developed enormously over the last 20 years [1]. Good quality guidelines are now widely recognised as useful authoritative statements of best practice across many clinical areas [2, 3]. Guidelines are typically produced for health care providers but there is an increasing interest in developing derivative products for the public and many organisations now produce patient versions of guidelines. In the UK, for example, the National Institute for Health and Care Excellence (NICE) and the Scottish Intercollegiate Guidelines Network (SIGN) produce freely accessible patient versions. The Finnish Medical Society, Duodecim, publishes patient versions of national Current Care guidelines and a comprehensive collection of guideline-based patient information in Duodecim’s Health Library. Professional groups are also producing patient versions of their guidelines, for example, the Netherlands Association of Post-traumatic Dystrophy.

For patient versions of guidelines to be useful, members of the public and patients need to know about them (or their health professionals need to know about them), the information within them needs to be pertinent to their healthcare decisions and that information has to be understandable. This is not always the case, as our earlier literature review found [4]. Others have also highlighted challenges, for example, that public and patients may perceive guidelines negatively as a way to ration access to medications [5], or that research information is often general when what people want is information that is very specific to them [6]. Indeed, even for guidelines directed at health professionals, alternative presentation methods, especially presenting information in layers, with the most important coming first, makes it easier for guideline to be used [7]. In other words, it is important to know what your users need, and in what format.

The aim of this study was to explore what patients and the public understand about the purpose and production of clinical guidelines, and what they want from clinical guidelines to support their healthcare decisions. It complements our earlier systematic review [4] and is part of the DECIDE project [8].

## Methods

The study involved focus groups with a heterogeneous purposive sample selected to represent a range of the likely users of patient versions of guidelines. This included individuals with selected health conditions (diabetes and depression), members of the public without experience of the topic of the guideline under discussion, a group of health communication professionals, and a group of young people (18 to 25). Diabetes and depression were selected based on the availability of voluntary organisations in the SIGN network that were able to facilitate recruitment and because they had been selected by the DECIDE project management group as being relevant to all countries within DECIDE. Nine focus groups were undertaken in total.

Participants were asked about their awareness and understanding of clinical guidelines and were presented with scenario recommendations, or draft materials from patient guidelines to prompt discussion (see Additional file 1). The questions and materials shown to the groups followed an iterative process of development and were revised for use with the different groups sampled. All focus groups took place between May and November 2012, in eight in locations across Scotland, and one in England. All were 30 to 90 min long. All focus groups were recorded and transcribed prior to analysis. Each group was facilitated by one or two researchers and hand-written notes were taken to capture relevant non-verbal communication.

The focus groups were supplemented with four semi-structured interviews with people experiencing homelessness, using draft patient guideline materials to prompt discussion. Interviews were conducted with this group because they provided a more flexible format that seemed to work better for the people experiencing homelessness. All interviews were audio recorded and transcribed prior to analysis. Focus groups were conducted by several members of the working group (MC, KG, KL, & ST).

### Participants

Participants for the focus groups were recruited through the Scottish Health Research Register (SHARE) [9], the Scottish Health Council (SHC), the Scottish Intercollegiate Guidelines Network (SIGN) network of voluntary organisations and the University of Dundee. The communication professionals were recruited with the help of Sense About Science [10]. The communication professionals were included as part of a heterogeneous sample of the public. They were purposively selected because communication professionals provide much of the material patients and the public receive. We were interested in their views on what the public and patients (i.e. their audiences) knew about guidelines and what opinions they had on how guideline producers could make their own health communication jobs easier. The communication professionals included journalists/multimedia managers and editors from UK based voluntary organisations, newspapers, journals and broadcasting companies. They all used guidelines as the basis for some of the material they produced for the public.

A total of 62 people participated in the focus groups; each group was composed of between four and 11 participants (see Table 1). Participants were recruited from a range of geographical locations across Scotland, with the exception of the focus group with communication professionals which took place in England. This group was held in London, England because it was a more convenient location for the participants. Communication professionals in England may often access different resources and guidelines than those based in Scotland but their general requirements are likely to be similar.

**Table 1:** Details of focus groups

Focus group	Participant type	Number	Gender	Age range	Location
G1	No medical condition. Recruited from Dundee University.	7	7 female	30s – 60s	Dundee, Scotland
G2	People with a diagnosis of diabetes. Recruited from Diabetes UK, via the SIGN network of voluntary organisations.	7	4 male, 3 female	40 - 75	Glasgow, Scotland
G3	No medical condition. Recruited from the SIGN network of patient organisations.	4	1 male, 3 female	45 - 75	Inverness, Scotland
G4	People with a diagnosis of depression. Recruited via the SIGN network of voluntary organisations.	7	3 male, 4 female	30 - 75	Edinburgh, Scotland
G5	No medical condition Recruited through Scottish Health Research Register (SHARE) and the University of Dundee.	7	6 male, 1 female	41 – 70	Dundee, Scotland
G6	Young people. Recruited by the Scottish Health Council.	5	2 male, 3 female	18-25	Glasgow, Scotland
G7	Health communications professionals including representatives from the voluntary sector, media, and academic journals.	11	3 male, 8 female	not recorded	London, England
G8	No medical condition. Recruited through Scottish Health Research Register (SHARE) and the University of Dundee.	9	2 male, 7 female	40s-60s	Dundee, Scotland
G9	No medical condition. Recruited through Scottish Health Research Register (SHARE)	5	1 male, 4 female	40s – 70s	Dundee, Scotland

The four people experiencing homelessness, one woman and three men, were recruited via National Health Service (NHS) Tayside Health Board.

### Ethics

Ethics approval for work done by KL and ST was given by the University of Dundee, where both worked at the time. Other aspects of this research were undertaken as part of SIGNs service evaluation. Information on the planned data collection for the DECIDE project was provided to the East of Scotland Research Ethics Service and they confirmed that it did not require full ethics committee review under the terms of the Governance Arrangement for Research Ethics Committee (GAfREC) in the UK. All participants were provided with study information, assured of confidentiality, and informed that they could withdraw consent at any time. All participants provided written consent.

### Data analysis

All analysis was carried out using QSR NVivo10®. NVivo® is software that supports qualitative and mixed methods research. The data were analysed using the framework analysis technique described by Ritchie & Spencer [11]. This method involves a number of separate but interconnected stages of rigorous and methodical qualitative analysis (Table 2 contains a description of the stages of framework analysis). It allows for the use of a priori themes linked to the study objectives, and for emergent themes to arise from the data. A number of themes were identified from the interview schedules and the previous work of DECIDE [4], however the majority of themes emerged directly from the data.

**Table 2:** Steps in the framework approach [12]

• Familiarisation – All data sources are read and sorted with reference to the study objective.
• Identify a thematic framework – Key issues concepts and themes are identified with reference to the objectives of the study and the work of the DECIDE project to date.
• Indexing – The thematic framework is applied to all the data sources.
• Charting *–* A matrix of findings for each theme is developed, allowing the comparison of different groups. Each cell of the matrix contains a summary of each group or individuals contributions to the theme, with a reference that can be directly linked to the original text.
• Mapping and interpretation *–* All charts and notes were compared to search for patterns and connections in the data. The process allows for the exploration of consensus and disagreement both within and across cases.

Two researchers (NF & JK) examined all the transcripts independently, and met to discuss and agree an initial thematic framework. Subsequently they carried out the indexing and charting stages independently and met to review and agree all findings derived from the data. The researchers who conducted the focus groups/interviews also examined the findings of the analysis, to ensure the themes identified were an accurate reflection of the material.

## Results

**Fig. 1:**
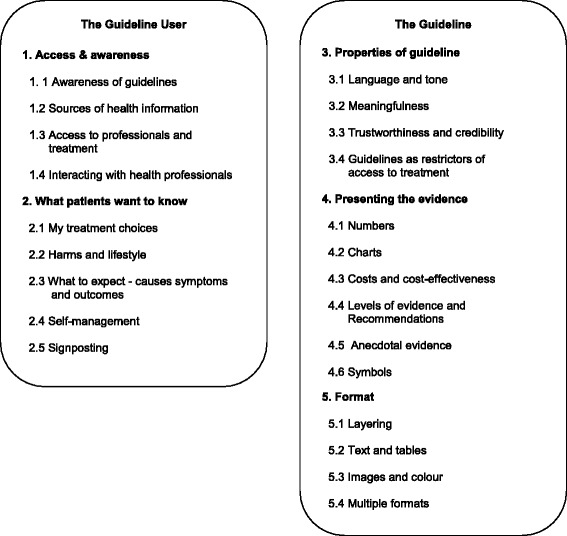
Thematic framework

The framework analysis yielded five themes, each with a set of sub-themes. The five themes have been grouped into two overarching themes: *The guideline* and *The guideline user*, as displayed in Fig. 1. The full analysis tables and participant quotes to further illustrating the findings of this analysis are available in the Additional file 2. The charting stage (see Table 2) of the framework analysis explicitly seeks differences and similarities between the samples gathered. Findings were largely similar across the samples, however all differences identified have been highlighted under the themes below.

### Overarching theme 1: The guideline user

#### Theme 1: Access and awareness (see Table 3)

##### Awareness of guidelines

Awareness of clinical guidelines overall was low with the exception of the communication professionals. This lack of awareness of the existence of clinical guidelines or patient versions clearly prevents people from accessing them and may underlie the low number of people in the sample that had done so. Awareness was higher in people with links to voluntary organisations/research networks, and people who work in a health or research field. Those that were aware of clinical guidelines generally had only a vague understanding of them and the type of evidence that underpins them.

Once made aware of patient versions of guidelines, there was overwhelming support for increasing access to them and the benefits that they could bring for patients:

*“If you’re seeking to empower, you’ve got to get the information to the patient…”* [P1, G2]

**Table 3:** Subthemes with illustrative quotes from the access and awareness theme

Subtheme	Illustrative quote	Participant & group number
Awareness of guidelines	Well the questions should be why is nobody asking for that [patient guideline]? And the response should be do your patients know they exist? If they say yes, well why have you still got them? If they say no you say well that’s part of the problem, you have to tell them.	P2, G2
Sources of health information	…I think nowadays when you’re, with any sort of diagnosis initially, whether it’s going to take you into the blackest hole in the world or not, the internet’s the place to go.	P1, G5
Access to professionals and treatment	You might have something else to ask but their body language you know if they’re bent over the computer, you might think that the time is up and you just think oh I’ll just go.	P3, G3
Interacting with healthcare professionals	…I don’t want to have to check on what GP is doing, I want to be able to trust them…but if I can’t, I need to have access to that information [patient guideline].	P3, G1

The participants with long term health conditions suggested that patient guidelines should be made available to all patients at diagnosis.

Despite the high value placed on health information, participants indicated that accessing good health information can be a challenge. They believed that the NHS is unable to prioritise the provision of good quality health information due to limited funding. Anger and frustration was expressed at the short-sightedness of this approach, given that information that enables self-management may reduce costs for health services in the long-term.

Under-use of the mainstream media in promoting patient guidelines was identified as a problem. The communication professionals group emphasised the failure of guideline producers to engage the public in accessing information directly from their websites. Conversely the other groups thought that newspapers and magazines could be used to promote patient guidelines and to inspire a more user friendly format for the information they contain.

##### Sources of health information

Participants identified three main sources of health information: healthcare professionals, the internet, and voluntary organisations. Unsurprisingly General Practitioners (GPs) were a key source of health information, and specialist nurses were an important source for people who had chronic health conditions or were experiencing homelessness. Older patients, and those without access to a computer, placed a strong emphasis on the availability of printed versions of patient guidelines from their healthcare professionals.

The internet was a widely utilised and valued source of health information. However, participants also found that it can be overwhelming and alarming because of the volume of available information. Identifying trustworthy sources of online information was important to the participants, and in this UK context, NHS websites were considered the most reliable.

Voluntary organisations were described as an excellent source of high quality user-friendly health information. Patients emphasised how helpful they had found them and expressed frustration over delays with signposting to non-health sector organisations (e.g. to weight-loss organisations) by health services. Communication professionals pointed out that voluntary organisations have organisational objectives and agendas that may lead them to disagree with guideline recommendations:

*“…charities have agendas and getting your, you know your advice on Abiraterone from our website will give you a very different view from NICE’s [National Institute for Health and Care Excellence] website simply ’cause we’re jumping up and down…trying to get it approved”* [P4, G7 Health Professionals]

##### Access to professionals and treatment

Waiting times and limited time with the GP during an appointment is a source of frustration for patients seeking health information. Lack of sufficient time with the GP for discussion of harms and contraindications was a particular issue for people with long-term conditions and/or multi-morbidity. The group of young people indicated that even the four-hour waiting time for a return call from NHS24 (a telephone helpline) [13] is too long for this to be a viable route to health information; by the time of a call-back they would already have found an alternative source. This theme of frustrations with access to, and limited time with, healthcare professionals was widespread and seemed to underlie the desire to be able to access health information directly and without the need to make an appointment with a healthcare professional.

A lack of fairness in access to good health information and treatment was highlighted by participants. Variability in the knowledge and communication skills of healthcare professionals, and the “*postcode lottery”* of which services are offered in a given location, leads to “*luck*” potentially playing a role in access to good health information and referrals.

##### Interacting with health professionals

A dominant concern for the participants was the failure of healthcare professionals (particularly GPs) to provide sufficient information on treatment options. If insufficient information on the range of treatment options was provided people felt unable to trust their healthcare professionals; even wondering if information was being purposefully withheld:

*“…the doctors are not always forthcoming as to what’s on offer.”* [P3, G3]

However, patients expressed a strong desire to be able to trust their healthcare professionals. The patients who did feel fully able to trust their healthcare professionals thought that they could rely on them to implement recommendations from guidelines and they struggled to see what use they would make of a patient version of a guideline. Conversely, those with doubts about how forthcoming their healthcare professionals’ are with information on treatment options clearly saw the usefulness of patient versions of guidelines. These issues of trust seemed to underlie the concern that healthcare professionals would react negatively to a patient bringing a patient version of a guideline to a consultation, or even raising its contents in discussion.

#### Theme 2: What patients want to know (see Table 4)

##### My treatment choices

Participants emphasised the need for information that enables them to choose between treatment options. Patient versions of guidelines were considered potentially empowering, in providing the information necessary for people to discuss specific treatment preferences, suggest alternatives, and refuse treatments suggested by healthcare professionals. To make informed choices participants wanted information on effectiveness but they wanted this information tailored, taking account of family history, co-morbidities, harms and lifestyle implications.

The importance of shared decision making, and the need for input from healthcare professionals when making a treatment choice, was a recurrent finding. It appears that some patients are being asked to make health decisions without the support from healthcare professionals that they would like, leaving them fearful of making the wrong decision:

*“… it’s putting so much pressure on patients, I had a friend…she was given loads of information and asked to make her decision. She said to me why are they putting it back on me?” [P1, G3]*

**Table 4:** Subthemes with illustrative quotes from the what patients want to know theme

Subtheme	Illustrative quote	Participant & group number
My treatment choices	…it [patient guideline] gives you the knowledge then it gives you the ammunition to question why you’re getting the treatment or why you’re not getting treatment.	P1, G5
Harms and lifestyle	…the doctor wanted to give me an operation…He didn’t tell me about the consequences right of what he was going to do.	P2, G5
What to expect - causes symptoms and outcomes	…we and our families are living with this illness, we should know what to expect…	P2, G2
Self-management	How to help yourself…Help yourself, yes, I think that’s the top priority [for patient guidelines]...	P3, G2
Signposting	Where you should go. It’s not a case of being spoon-fed because we’re all adults but if you’re suffering from this or have the signs of this, this is where you can go for help? We need to know that.	P6, G2

##### Harms and lifestyle

The provision of information on harms was a priority for the participants. It was considered fundamentally important to making an informed treatment choice. In order to do so patients' required balanced information that allowed them to judge whether the benefits of a treatment outweighed the risks for them as an individual.

Unfortunately participants did not think that healthcare professionals placed as much emphasis on potential harms of treatment as patients do. Concern was expressed about a lack of information provision about the risks, and lifestyle implications, of both medicines and surgical interventions. Less commonly concerns were expressed about the minimisation of potential risks and side-effects by healthcare professionals.

##### What to expect - causes symptoms and outcomes

Participants wanted good quality information about what to expect from an illness, and health services throughout its entire course. This includes information on symptoms, treatments, causes and possible outcomes. The communication professionals group highlighted clinical care pathways (i.e. the stages of care that will be delivered to a patient, by which professional and when) as a helpful way to communicate this information.

##### Self-management

There was consistent demand for patient versions of guidelines to contain information that enables self management. Study participants were very interested in information about any ‘*easy*’ ways that they can change their lifestyle to help to manage a condition. For example groups that were shown obesity guideline materials emphasised the need for information about how to increase exercise levels and improve diet. Participants showed an awareness of the impact of financial drivers behind the provision of health information; they were keen to point out that good quality health information, focused on self-management, reduces the need for input from health services:

*“Don’t forget the more information we get, the better we can look after ourselves. If we have these guidelines, you see him [GP] less…”* [P2, G2]

##### Signposting

Patient versions of guidelines should contain information that refers users to appropriate sources of support and places to access treatment. Participants made it clear that it is not sufficient to provide links to websites when signposting an organisation. This was key for some groups such as older people, for whom information on services that can be accessed by telephone or by personally dropping into a health centre is particularly important.

### Overarching theme 2: The guideline

#### Theme 3: Properties of guidelines (see Table 5)

##### Language and tone

A dominant theme was the need for simple language in patient versions of guidelines. This included minimising the use of medical and technical language, keeping sentences and paragraphs short, and avoiding ambiguity. Medical terminology was identified as the key barrier to patients and clinicians using the same guideline. In fact, the use of language that the majority of the public cannot understand can be interpreted as purposefully misleading:

*“…in case you get a doctor who doesn’t want people to know much, and then he writes it in medical terms so it, it completely blinds you” [P2, G5]*

**Table 5:** Subthemes with illustrative quotes from the properties of guidelines

Subtheme	Illustrative quote	Participant & group number
Language and tone	“…there was a lot of medical jargon…I know from experience and you know congratulations for producing this booklet [SIGN patient guideline] but patient themselves cannot all read it…	P2, G4
Meaningfulness	There’s no point in teaching somebody, or getting somebody to look at how the guidelines are produced when they’re actually wanting to find out ”What Is Obesity"?	Interview 1
Trustworthiness and credibility	…it gives it more weight if it’s based on a guideline…because you assume…even…if you don’t understand exactly what a “guideline” means…that it’s based on some evidence from somewhere that this is a good thing to do, someone’s looked at some evidence and made a guideline…	P4, G1
Guidelines as restrictors of access to treatment	…CBT is the one that doctors know about but it’s not the only solution, and I think that this idea that because there is evidence then you’ve got to refer people to CBT, I think it’s really really dangerous…	P1, G4

Participants frequently found the tone of patient version guideline materials negative and dogmatic, particularly when a lack of evidence for an intervention was highlighted. Terms such as “*there is no good evidence*” were considered ambiguous and unhelpful. In contrast any attempt to emphasise the positive was more appealing.

##### Meaningfulness

It must be evident quickly that a patient version of a guideline is aimed at the patient group concerned and contains information on what the patient wants to know to encourage them to read it. Design aspects are important in appealing to the user group including the use of meaningful images and colour. Content and lay out is also key, for example participants did not want information on how guidelines were produced to be overly prominent, since this distracted from the information on the condition and how to manage it.

The usefulness of patient involvement in research and guideline development was highlighted. This can greatly increase the meaningfulness of the guideline to the patient, particularly in ensuring that key outcomes are reported on:

*“I think it’s important that they do go to Patient Groups when they’re doing their guidelines…because patients will soon tell you, you know what matters to them about coping with their condition…”* [P3, G1]

##### Trustworthiness and credibility

The source of guidelines’ credibility is the understanding that they are based on a thorough review of the available evidence. The threats to guideline credibility that participants noted included healthcare professionals’ ability to disregard them, the perception that strong evidence for effectiveness is ignored if the costs of a treatment are high, and changes to recommendations (i.e. something is said to harmful and later said to be beneficial, or vice versa). An example of this was confusion resulting from changes to government guidelines e.g. safe consumption of alcohol. Providing a clear rationale for any changes may aid acceptance and minimise patients’ frustration. Concerns about the role of financial considerations extended to potential conflicts of interest in pharmaceutical companies and manufacturer’s funding of research and information for patients. NHS branded information was considered the most trustworthy.

##### Guidelines as restrictors of access to treatment

There was a strong concern about guideline recommendations leading to decreased access to treatment, from groups other than the communication professionals. The perception that the decision to not recommend an intervention is based on costs rather than effectiveness leads to a negative perception of guideline bodies. In contrast, the communication professionals saw UK guideline bodies in a positive light. They considered them (e.g. NICE) to be objective and to represent a gold standard in guideline methodology. They suggested that this misperception of guideline bodies could be addressed by a more sophisticated media management strategy.

The lack of evidence for complex interventions “*that just aren’t easy to trial…”*, or that fail to attract funding for research, is also of concern. People fear that clinical guidelines will lead to access to these interventions being withdrawn or referrals to treatments with a stronger evidence base than their preferred option.

#### Presenting evidence (see Table 6)

##### Numbers

The participants fell broadly into two groups in relation to numerical information. The first group found information presented in a numerical format helpful, direct and credible. The other group found tables containing fairly complex numerical information off putting and suggested supplementing them with simpler text or graphics. The communication professionals highlighted the difficulty of interpreting relative risks without information on absolute risk. They also strongly supported the use of the simplest numerical information, such as frequencies, when communicating with patients.

Participants indicated that numerical information can appear cold and dehumanising, presenting the patient with a description of the health problem that is difficult to relate to. Personal stories were able to counterbalance this by putting a human face to the data being presented. However, personal stories can be alienating if the reader does not recognise themselves in the story, or potentially misleading if only one possible outcome of an intervention is presented. Some potential patient guideline users found personal stories unnecessary and preferred the presentation of more numerical information.

**Table 6:** Subthemes with illustrative quotes from the presenting evidence theme

Subtheme	Illustrative quote	Participant & group number
Numbers	…in numbers rather than percentages … so if this many people do this, then…this many people benefit, but…this many people may have a serious side-effects…	P8, G7
Charts	…I personally will look at graphs and, and, and pie charts and things, but if I saw…this [Prostate cancer screening graphic] I, I just would’nae …I’d just scoot, scoot over it”	P5, G5
Costs and cost effectiveness	I don’t think you need that information [on cost-effectiveness] because …that’s down to the, the NHS…it’s not down to the patient.	Interview 4
Levels of evidence & recommendations	I wouldn’t want to hear it [a recommendation] was weak…I would take it with a pinch of salt if it’s based on weak evidence and actually I would think what are they doing producing a recommendation if it’s not strongly endorsed.	P3, G3
Anecdotal evidence	“I joined self-help groups, I think they’ve been helpful to me but there’s no research evidence to say how effective they are whereas there’s medication and talking therapies which are quite reasonably researched, you can see the evidence for them. Other things can be just quite as helpful.	P7, G4
Symbols	“What the wee ones wi’ the plus in them?…because what is that for?	P10, G8

##### Charts

Simple charts were greatly appreciated for their ability to convey a substantial amount of information quickly and in a visually attractive format. The communication professionals thought that online *“graphics really work” to* convey complex health information, such as risk and benefit ratios. However, the participants in other groups again fell into two broad groups, one expressing a preference for extracting meaning from charts and the other preferring a text explanation of evidence. It seemed therefore that both were necessary for all the participants to make sense of the material.

The simpler and more recognisable the chart (e.g. a simple bar chart) the more usable and well received it was. A dominant reaction to more complex charts and graphics was frustration and confusion and the suggestion that this material would be discarded or skipped over.

##### Costs and cost effectiveness

The presentation of any material on costs and cost-effectiveness was disliked and criticised as irrelevant to patients. They suggested that they “*don’t need to know about costs*”. This irrelevance appeared to stem from a demand for information that helps to choose between treatment options or enables self-management, while decisions about costs are “*for the NHS*” not the patient. The anger that costs information could trigger was strongly linked to the perception that recommendations against interventions are driven by costs and not effectiveness, and general concerns about the funding of healthcare.

In stark contrast to the other groups, the communication professionals wanted information on costs to be more accessible, so that the underlying rationale behind an intervention not being recommended is more evident. Providing an understandable rationale for decisions around a recommendation is extremely helpful and may stop people from assuming that decisions are entirely based on cost.

##### Levels of evidence and recommendations

There was widespread surprise and confusion caused by the possibility that recommendations, and interventions themselves, may not have a strong evidence base underlying them. This was, at least in part, due to unfamiliarity with the concept of levels of evidence, in particular the notion of poor quality research. Any references to ‘moderate’ or ‘low’ quality evidence or the use of symbols (e.g. the one to four plus signs used in GRADE [14]) to provide information about the quality of evidence was challenging for the groups to understand. The most widespread view was that information on the quality of evidence underlying recommendations was unnecessary in a patient version of a guideline; however a less commonly held perspective was that it would be misleading to withhold this level of detail.

Overall, the participants did not favour the use of levels of recommendation in patient versions of guidelines and did not understand why such a guideline would include a recommendation with a large amount of uncertainty attached to it. When recommendations were described as ‘weak’ (as done in GRADE), a strong negative reaction was expressed to the tone of the word itself, irrespective of the concept underlying it. While participants understood that a weak recommendation indicated that the recommendation is less strongly “*endorsed*” than a strong recommendation they interpreted this in a variety of ways. This included: as an indication that the intervention itself is not effective, that the intervention is supported by low quality evidence, or that the guideline developers are not certain about this recommendation. Participants wanted an honest explanation of the rationale behind a recommendation. Therefore avoiding the use of the term weak and instead giving an understandable explanation of the reasons for uncertainty was considered the best approach to explaining levels of evidence and recommendation.

To be meaningful to the patient, a patient version of a guideline has to strike a balance between being too simplistic and being overly complicated and technical. The amount, and complexity, of information contained in some materials shown to participants led to ‘*information overload*’. They suggested that faced with this level of complexity they may abandon the patient version of a guideline and find something simpler. However, participants were keen to emphasise that ‘*one size does not fit all*’ and what might be perceived as insufficient, or overly complex, information by one person may be ideal for another. This was reflected in a strong concern for the provision of appropriate information for people lacking in health literacy and for people with conditions that impair cognition (e.g. depression). A sub-group of our participants, including but not exclusively composed of the communication professionals, wanted access to an in-depth summary of the evidence underlying a recommendation.

##### Anecdotal evidence

The importance of personal experience and stories of what has happened to family members, friends and celebrities in making health decisions was a strong theme, and it was apparent that this can override evidence presented to patients. Anecdotal information about harms was a particularly recurrent aspect and seemed to have a powerful impact:

*“I’m quite interested in the fact that, does it say Warfarin doesn’t, doesn’t em ’cause any major lifestyle changes…yeah whereas people I know have been on it just hate…you know hate being on it because of the lifestyle implication”* [P1, G9]

Participants found it hard to understand why interventions that they had found personally helpful would not appear in a patient version of a guideline. This may be linked to another commonly expressed view that any treatment is “*worth a shot*”, regardless of the evidence of effectiveness, if a person’s quality of life is severely affected by a condition. While some people did indicate that they would be “*put off*” a treatment by a lack of evidence, many suggested that they would still want to try it if there was any chance it might be helpful. Descriptions of “*natural treatments*” and any intervention that people believed does no harm (an example given by participants was self-help groups), as lacking an evidence base were considered overly negative.

##### Symbols

The G8 group and the people experiencing homelessness were shown mock guideline materials that used plus symbols to communicate information about levels of evidence (see Additional files 3 and 4). Participants found the use of these symbols confusing, even when a key was presented on the same page. For the people in our sample that did not have a healthcare or research background, communicating meaningfully about levels of evidence using these symbols did not work, and a text explanation was also necessary.

#### Format (See Table 7)

##### Layering

There was consistent and wide-spread support for layering of information in electronic formats because it allows access to more complex evidence while keeping the surface level simple (for examples of layering in guidelines see Kristiansen 2015 [7]). This was highlighted as a solution to the problem of providing tailored information and a key benefit of online information. People with multi-morbidity (we did not specifically recruit people with multi-morbidity but participants self-identified as having multiple conditions during the research process) emphasised the importance of information that actively takes account of their health conditions. While younger people were very interested in technology that allows layering of information to the exact level required (e.g. apps), older people can experience referral to online information and other communication technologies as alienating and frustrating.

**Table 7:** Subthemes with illustrative quotes from the format theme

Subtheme	Illustrative quote	Participant & group number
Layering	I’d quite like a guideline that was on the internet and it had, if you wanted more information “click here” right and you could then progress…depending on how much information you wanted.	P3, G1
Text and tables	…not too many words close together. People will look at it and think ‘oh I don’t want to read that’ like seeing a massive text and it’s too much.”	P2, G6
Images and colour	…like a caricature, like sort of pointing to the throat or something like a, a man with a…you know so we know what we’re talking about…	P6, G5
Multiple formats	…they should be in a variety of formats, apps, internet so that anyone can source them…	P2, G4

##### Text

There was broad support for any form of chunking [15] in the text, including clear, headed sections, bullet points, and tables which were liked for their ability to convey factual information quickly and clearly. Participants indicated that long sections of unbroken text are likely to be skipped over until something more attractive catches the reader’s eye; the shorter and simpler the text is the more usable the patient version appears to be guideline. This is particularly true for information produced as a hard copy, which should be kept short, and focused, to appeal to the majority of readers. Although it should be noted that “*one size doesn’t fit all*” and facilitating access to more detailed information will be appreciated by some.

##### Images and colour

Images were found helpful when they conveyed meaning, such as making a topic obvious at first glance. Images draw attention to a particular section of text, which helps to break it up and make the guideline more usable and attractive.

The use of images can make a guideline appear more “*personal*”, and help to humanise the information being conveyed, giving the patient guideline a more friendly appearance. Conversely inappropriate, overly clinical or poor quality images may make a patient version of a guideline less attractive and give it a “*cheap*” appearance.

The use of colour also makes a guideline more attractive and therefore more likely to be read. It is important to be aware of peoples pre-existing associations with colour, for example red and its association with danger. The use of dull and indistinguishable colours in charts, such as grey and white, was found unhelpful and confusing, as was too many bright colours.

##### Multiple formats

Multiple formats are necessary if patient versions of guidelines are to be disseminated to the widest possible audience. Younger people appeared to particularly value the anonymity, and speed, offered by apps and Quick Response (QR) codes. However all age groups recognised that only a minority of people can currently access patient versions of guidelines in this way, so they can only supplement other formats. Patient versions of guidelines that were available in multiple formats, such as simplified versions, hard copy, large text, and video/audio formats may particularly benefit vulnerable groups.

## Discussion

The main aim of this study was to explore what patients and the public understand about the purpose and development of clinical guidelines, and what they want from clinical guidelines to support their healthcare decisions. The individuals who contributed to the study represent a range of people who may access patient versions of guidelines, including people with long-term conditions, younger people, homeless people, and other members of the public, as well as healthcare communication professionals who use guidelines as the basis for the materials they produce for the public.

The main message from this study is that those charged with the development and implementation of clinical guidelines have a considerable amount of work to do to increase awareness in the first instance and make patient versions of guidelines acceptable and useful to patients. Five themes emerged: Access and awareness, What patients want to know, Properties of the guideline, Presenting the evidence, and Format. These overlap with the themes coming from our systematic review (with which NF and JK, who led the analysis in the current study, were not involved): Applicability of guidelines, Purpose of guidelines for patient, Purpose of guidelines for healthcare system and physician, and Properties of guideline [4]. Again we conclude that these themes need to be incorporated into the design of patient versions of guidelines if they are to be useful to the public and patients.

The public is generally unaware of the existence of guidelines, though people are enthusiastic about them once they are made aware of them. It is unlikely that a guideline website is the first port of call for the majority of people looking for health information, meaning signposting by health professionals (or their organisations, such as the NHS) is important. The professional health communicators group mentioned the importance of making guidelines easy for charities (several members of this group worked for charities) to access and understand so that they can incorporate guideline information into their materials for the public. The relevance of this advice is easily demonstrated by a Google search of ‘diabetes treatment’, which, ignoring paid-for ads, puts the charity Diabetes UK as the #1 search result, with NICE at #17 on the second page of results. NHS Choices was #2, emphasising the importance of using brands that the public may be more familiar with and, as our study found, are already trusted.

Participants emphasised the need for information that enables them to choose between treatment options, which includes information on harms. Moreover, they would like help with this from healthcare professionals. Treatment harms will always be of concern to patients [16, 17] and healthcare professionals need to be aware that health information they provide may be competing with anecdotal information. Making the trade-offs between harms and benefits, especially when they are close, is an area where guideline-based tools to support shared decision making could have an important role, a point made by others linked to the DECIDE project [18]. Members of same group suggests that production of these tools can be semi-automated as demonstrated in the MAGIC guideline project for antithrombotic therapy [19]. What members of the public do not want is to be handed a lot of material and then left to make their own decision, which is a narrow view of shared decision making [20]. The public wants input from professionals.

Presenting risk information in patient versions of guidelines is challenging [4]. Participants in this study often differed in their support for numerical information and graphs, and suggested that there is no one-size-fits all approach. This has led DECIDE to suggest layering of information in guidelines [7] with the most important information (generally the recommendation) being presented first and then users can access more detailed information if they want it, down to the full evidence profile. Layering is possible even within a paper document using colour or boxes, as done by the Scottish Dental Clinical Effectiveness Programme in their patient guideline on preventing gum disease [21] and SIGN in their glaucoma guideline patient version [22], which attracts readers to the recommendations first.

### Strengths and limitations of this study

A strength of the study is that it involved a wide range of participants from many backgrounds and from across Scotland. The extent to which the findings are transferable to other populations, particularly non-UK populations, is uncertain. Qualitative research does not seek to gather a representative sample of a population but rather to purposively gather a heterogeneous sample and explore the transferability of the results beyond the sample gathered. The findings presented here do concur with those from our earlier systematic review [4], which was global in scope. We are confident about the wider relevance of the five themes that emerged from the focus groups and interviews.

A rigorous method of qualitative analysis was used to analyse the data, which was led by two researchers (NF and JK) working independently of each other and who were not involved in the data collection, or the earlier review. This has ensured that the themes emerged from the data, rather than being imposed upon it by the rest of the team. Finally, we have made our findings tables available as a supplemental document so that others may review it and potentially use if for their own work.

The sample was purposively selected and heterogeneous in nature and therefore the materials and the exact questions posed to the participants varied somewhat between the groups. The materials and questions were tailored to the groups and developed as the research progressed. Each focus group/interview focused around the topic of what each group understood about and wanted from clinical guidelines and was open in its structure. This did not hinder the analysis. Given the focus of the research question and the range of groups included, the focus of the analysis was on similarities across groups and where discussion moved away from guidelines this material was excluded from the analysis. The analysis process specifically sought to identify differences between groups at the charting stage (see Table 2), and where differences were found they have been highlighted.

There are issues of trust that need to be addressed alongside efforts to clearly convey the balance of risks and benefits associated with particular treatments or courses of action and the importance of evidence when making potentially life changing decisions. The GRADE process [23] has been instrumental in clarifying what that balance might be for specific interventions, and includes the values and preferences of patients and healthcare professionals. DECIDE has built on that work to improve the presentation of that information in ways that are meaningful to different audiences, including patients.

## Conclusions

Members of the public want information to help them choose between treatments, including information on harm, particularly to support shared decisions with healthcare professionals. Presenting numerical information is a challenge and layered approaches that present information in stages may be helpful. Guideline producers should carefully consider the themes identified in this study, together with those from our earlier review [4], so as to avoid producing materials that fail to support public and patient healthcare decisions.

The work coming from this study helped to support the update of the Guideline International Network Toolkit Chapter 4 on developing patient versions of guidelines [24].

## Additional files

Additional file 1:
**Focus group topic guides.** The topic guides used for the focus groups. (PDF 703 kb) Additional file 2:
**Findings table with supporting quotes.** A qualitative analysis table displaying the findings of the analysis with supporting quotes and notes. (PDF 887 kb) Additional file 3:
**Depression focus group mock guideline materials.** The mock guideline materials shown to the focus group with people with depression (PDF 134 kb) Additional file 4:
**Healthcare scenario workbook.** Healthcare scenario workbook used for interviews with people experiencing homelessness. (PDF 958 kb)
