# Supplementary material for: What do patients and the public know about clinical practice guidelines and what do they want from them? A qualitative study
Source: BMC Health Serv Res. 2016 Feb 24;16:74. doi: 10.1186/s12913-016-1319-4 (PMC4847193; doi:10.1186/s12913-016-1319-4)
Supplement: Additional file 1: — Focus group topic guides. The topic guides used for the focus groups. (PDF 703 kb) [file 12913_2016_1319_MOESM1_ESM.pdf]

Additional file 1

Focus group topic guides

G1

9th May 2011
Materials: Scenario used was the “Cervical cancer screening programme” with the topic guide for a focus group interview.
Scenario 1 – text 1: There is a well-established cervical cancer screening programme in Scotland for women between 20 and 60. It is recommended that women be screened. However, some GPs may not be recommending screening to their patients.
Q: What do you think about this?
Q: Did you know that guidelines for healthcare exist? .
Q: Do you think they are perceived as something positive or negative?
Q: if had guideline for treatment, if you wouldn’t use them to discuss with doctor, would you use them to discuss treatment with friends and family.
Q: So if you had cervical cancer would you be interested in knowing about these guidelines?
Q: Where do you go now for healthcare information?
Q: How do you know what you are reading is worth reading?
Q: Do you want to know all this information?
Q: Layered information
Q: Should there be patient version of guidelines?
Clinical Scenario – Going back to the cervical cancer screening programme. What if I told you that when the guideline committee was originally making their guideline about whether women between 20-25 should be screened, the recommendation was weak.
Q: What do you think weak means?
Q: So would you like further information about genetic aspect?
Q: So maybe could press on link like you talked about before to get further information.
Q: So what do you think if weak or strong evidence in guidelines?
Q: Do you want to hear about harms as well as benefits of a treatment.
Q: Have you all heard about NICE?
Q: Do you think about NICE in a negative or positive way?
Q: Is there anything else you would like to say about guidelines that we haven’t discussed today?

G2, G3, G4

Scenario

The [Scottish Intercollegiate Guidelines Network, or SIGN] has published guidelines recommending that people with severe asthma receive steroids. But we know that only 25% of people with severe asthma are receiving them. So, many doctors are not following these guidelines.

What do you think about this?

Follow-up questions about:

- Did you know that guidelines for care existed?
- Are they perceived negatively? Are they about rationing, do they help or hinder doctors?
- Where do you go for healthcare information?
- How do they use the information? To support an immediate decision, to reinforce what they already know, to get general information about an illness/disease?

If the people with severe asthma knew about the guidelines, would it be useful? How?

Follow-up questions about:

- Positive or negative comments about whether useful and why (anxiety, useful, empower patients, just want to know what to do)
- What patients could do (e.g. increased knowledge, talk to doctor, know what to ask about, use in decision making)
- What pieces of information would be helpful (e.g. who information applies to, reasons behind recommendations, quality of research, guideline producer, benefits and harms of treatments, description of condition or treatment)
- If you had (or have) severe asthma, would you be interested in knowing about these guidelines? Why?

How should the guidelines be presented to patients and by whom?

Follow-up questions about:

- Printed, internet (where – consumer organisation websites?), video.
- From doctor, consumer organisations, guideline producers.
- As narratives, personalised, personal stories, tables, short list, short summary.

Let's go back to the scenario….What if I told you that when the guideline committee was originally making the guideline about whether doctors should give steroids or not, the recommendation was 'weak'. What difference could this make as to whether doctors prescribe steroids or not?

Follow-up questions about:

- Comments about difference between a 'weak' or 'strong' recommendation, patients value different things, weak is low quality evidence, close call between benefits and costs, benefits and harms
- Do you think patients want to know something about how confident we are about the strength of the recommendation?

<u>G5 & G8, G9</u>

These focus groups used draft depression PCGL materials.

Before turning over the page:

Imagine that you, or someone that you know, is suffering from depression. You want to know what could help you. You are given this information by a doctor, nurse or another healthcare professional.

1. Where would you look for information?
2. What sort of information would you want to know? (What would you want the guide to include)?
3. I am now going to show you some information about depression. When I turn the page over can you give me your first spontaneous impression? Don't think about it; just say the first thing that comes into your head.

Now turn over to page 1.

4. Can you read the first section (some background) and tell me what you think about it? (Is it clear? Is it useful? Why? Do you understand the purpose of the symbols? Do you think that there is a better way to explain this? Do you know what the QR codes ARE and what do you feel about them? Would you know how to use them?)
5. Can you read the next section now (what are the best ways to make myself feel better)? Can you tell me what you think about it? (Is it clear? If you felt depressed would this advice be helpful to you? Are there ways in which the information could be presented in a more helpful way?)

Now turn over to page 2.

6. What is your first impression of this page?
7. We're interested in testing different types of graphs to see if they can be easily understood. Can you tell me what you think this graph means? (This is not a test of you - it is a test of the graph!) Do you like numerical information presented in graphs? How do you like numerical information to be presented?)
8. Now read the section on computerised CBT. (Is it clear? If you or someone that you knew was depressed would you find this useful?) What do you think about the information on cost effectiveness?
9. Now read the section on self help. (Is it clear? is it useful? What do you think about what it is saying about self-help? If you felt depressed what would you do about this advice?)

Now turn over to page 3.

10. What is your first impression of this page?
11. Now read the section on exercise. (is it clear? Is it useful?
    What do you think it is saying about exercise? If you felt depressed would you follow this advice?
12. Now read the two sections on diet and nutritional supplements. (Is it clear? Is it useful? What do you think it is saying about diet and nutritional supplements? If you felt depressed would you follow this advice? Why / why not?)

Now turn over to page 4.

13. What is your first impression of this page?
14. Can you read the section on complementary therapies?
    (Is it clear? Is it useful? What do you think it is saying about diet and nutritional supplements? If you felt depressed would you think about using complementary therapies? Why / why not? Has your opinion on complementary therapies changed as a result of reading this?
15. What do you think about this document overall?
    Does it cover what you wanted to know? What's missing? Is there anything that is included that you didn't want to know? Would you feel that you knew more about coping with depression as a result of reading this? Would it help you to have a discussion with your doctor or nurse? Would you prefer it to be presented in other ways?

G6

Diabetes scenario

The Scottish Intercollegiate Guidelines Network has published guidelines recommending thatpeople with Type 1 diabetes (ie. where a person does not produce insulin) should self-monitor their blood sugar levels. The guidelines also suggest that some people might benefit if they continuously monitor their blood sugar throughout the day using a special device. Doctors may choose to not offer the device.

What do you think about this?

Follow-up questions about:

- Did you know that guidelines for care existed?
- Are they perceived negatively? Are they about rationing, do they help or hinder doctors?
- Where do you go for healthcare information?
- How do they use the information? To support an immediate decision, to reinforce what they already know, to get general information about an illness/disease?

If the people with severe asthma knew about the guidelines, would it be useful? How?

Follow-up questions about:

- Positive or negative comments about whether useful and why (anxiety, useful, empower patients, just want to know what to do)
- What patients could do (e.g. increased knowledge, talk to doctor, know what to ask about, use in decision making)
- What pieces of information would be helpful (e.g. who information applies to, reasons behind recommendations, quality of research, guideline producer, benefits and harms of treatments, description of condition or treatment)
- If you had (or have) severe asthma, would you be interested in knowing about these guidelines? Why?

How should the guidelines be presented to patients and by whom?

Follow-up questions about:

- Printed, internet (where – consumer organisation websites?), video.
- From doctor, consumer organisations, guideline producers.
- As narratives, personalised, personal stories, tables, short list, short summary.

Let’s go back to the scenario….What if I told you that when the guideline committee was originally making the guideline about whether doctors should give steroids or not, the recommendation was ‘weak’. What difference could this make as to whether doctors prescribe steroids or not?

Follow-up questions about:

- Comments about difference between a ‘weak’ or ‘strong’ recommendation, patients value different things, weak is low quality evidence, close call between benefits and costs, benefits and harms
- Do you think patients want to know something about how confident we are about the strength of the recommendation?

<u>**G7**</u>

1. Cervical cancer screening scenario

There is a well-established cervical cancer screening program in Scotland for women between 20and 60. It is recommended that women be screened. However, some GPs may not be recommending screening to their patients. What if I told you that when the guideline committee was originally making the guideline about whether women between 20 and 25 should be screened, the recommendation was ‘weak’.

2. Bronchitis scenario

The Scottish Intercollegiate Guidelines Network has published guidelines recommending that antibiotics should not be prescribed for bronchitis. But many doctors still prescribe antibiotics for bronchitis and do not follow this guideline. What if I told you that when the guideline committee was originally making the guideline, the recommendation to not use antibiotics for bronchitis was ‘strong’.

Topic guide (about 1 hour)

Introductions – Facilitators, participants (5 minutes).
General – Hand out the DECIDE (Developing and Evaluating Communication strategies to support Informed Decisions and practice based on Evidence) general leaflet, talk about the aims of DECIDE (5 minutes)

What do you know about guidelines?
Follow-up questions:
• Who and what do you think healthcare guidelines are for?
• Do you think they are perceived as something negative or positive? Are they about rationing, do they help or hinder?
• What might make a guideline newsworthy from your point of view, or that of your editor?
• When thinking about healthcare guidelines, what role do you think journalists have?

Is giving more space to healthcare guidelines a good idea?
Follow-up questions:

• If your publication gave lots of space to discussing guidelines, would this bring any benefits? Would knowing more increase people’s knowledge, allow them to talk to their doctor, know what to ask about, use in decision making, shared or individual?
• What pieces of information do you think would be helpful for readers, viewers or listeners? (e.g. who information applies to, reasons behind recommendations, quality of research, guideline producer, benefits and harms of treatments, description of condition or treatment)
• What are the key parts of a guideline that you think the public might be interested in?
• Should there be ‘patient versions’ of guidelines?
• How could guideline producers make your job easier?

Thinking about uncertainty and recommendations
Have a look at the scenarios.
Follow-up questions:
• What do you think ‘weak’ means in the first scenario?
• What do you think the difference between a ‘weak’ or ‘strong’ recommendation is? Patients value different things, weak is low quality evidence, close call between benefits and costs, benefits and harms, was this a badly done guideline?
• Do you think patients want to know something about how confident we are about a recommendation? How should we deal with uncertainty?
• What do you think the best way of presenting this sort of information would be?
Are there any other points you would like to raise?
