# Supplementary material for: What do patients and the public know about clinical practice guidelines and what do they want from them? A qualitative study
Source: BMC Health Serv Res. 2016 Feb 24;16:74. doi: 10.1186/s12913-016-1319-4 (PMC4847193; doi:10.1186/s12913-016-1319-4)
Supplement: Additional file 2: — Findings table with supporting quotes. A qualitative analysis table displaying the findings of the analysis with supporting quotes and notes. (PDF 887 kb) [file 12913_2016_1319_MOESM2_ESM.pdf]

Additional file 2

Findings table with supporting quotes

Overarching theme 1: The user

Theme 1.1: Awareness of guidelines

Findings: 1.1: Awareness of guidelines	Key quotes	Notes
Awareness of clinical guidelines and patient versions of clinical guidelines was low. Awareness was higher in people with links to voluntary organisation, research networks, and people who work in a health or research field.	*"I don't think people, sort of the general public knows anything about them really, you know they don't know [yeah] …"* [P2, G1 cervical cancer group]<br><br>*"I've never seen guidelines.....I'm a type 2 diabetic, I've had it since I was a child, I wasn't aware.... I didn't know......."* [P6, G2 Diabetes group]	Awareness was low in most groups, none of the homeless interviewees or young people had heard of clinical guidelines. Awareness of guidelines was high amongst professionals who worked in a health related field e.g. the communications professionals and in some of the participants associated with voluntary organisations who had long term health conditions e.g. G2 diabetes group.
The majority of people thought that increasing awareness and access to patient versions of clinical guidelines would be of great benefit to patients, and distributing them should be a priority for health professionals.	*"If you're seeking to empower, you've got to get the information to the patient; GPs are not providing that information from guidelines on choices to the patients."* [P1, G2 Diabetes Group]<br><br>*"Well the questions should be why is nobody asking for that [patient clinical guideline]? And the response should be do your patients know they exist? If they say yes, well why have you still got them? If they say no you say well that's part of the problem, you have to tell them."* [P2, G2 Diabetes group]	This is supported across the groups. However, the diabetic group were particularly focused on making patient clinical guidelines more accessible and favoured compulsory provision, the depression group was also particularly strong in their support of patient clinical guidelines. Perhaps people with long-term conditions that require

	"It's amazing that we don't know about the guidelines, that's the first time I've seen them, we should have them, we should say to doctors where do we get them?" [P2, G2 Diabetes]<br><br>*"When I was newly diagnosed I went on a course, now the specialist nurse who runs the courses would have been delighted to give me these if she had them. There's a problem that you have in getting these to the patients." [P1, G2 Diabetes]*<br><br>*"You need to get this out to people, the shortened version. Now you've got the main guideline and this one is more detailed and suitable for the public and people need to get this"* [P2, G4 Depression Group]<br><br>*"... we have to give patients access to this I feel we can't let a resource like this go to waste, we're lacking.....a general patient source with solutions and routes and it's important that people are aware of this information source how they can improve the situation for themselves" [P2, G4 Depression]*<br><br>*"...I feel as if I had got that there and then it would have been a lot better for me. The guideline should automatically be sent to people who are newly diagnosed; in fact the guidelines should be given to everybody really." [P2, G2 Diabetes Group]*	considerable self management find patient Clinical guidelines more helpful. They may also be more angered by the current lack of information provision.
Most people that had accessed patient Clinical guidelines before had done so via voluntary organisations, or because of their professional background.	*"I've got the guidelines but the only reason I've got them is because a representative came to one of our support meetings and handed them out. I did not get them from the NHS or any other source."* [P1, G2 Diabetes]<br><br>*"it has the title sort of Allograft Transplantation, it's quite a complicated title, but it's aimed at patients, I thought that's great, you know if people ask us about that then I can send them this document, but I'd be amazed if they found it, you know…"* [P3, G7 Communication	All of the communications professionals and the group from university of Dundee/research network had heard of guidelines. Some participants in the groups that came from voluntary organisations had heard and seen patient clinical guidelines. Otherwise awareness and access

	Professionals]	was low.
The media could be used more effectively by guideline producers to promote patient versions and increase dissemination.	*"...do you think there's more of a role for this to get this information (patient version) out? You know the printed media, the public media, Scotsman and Herald and the likes so patients and carers and families are aware....and they can try to understand them." [P3, G4 Depression group]*<br><br>*"when you get some headline talking about a celeb.... I'm sure it would turn the heads of a lot of people who wouldn't pick this up, but would pick up Hello magazine....So in some way feeding in that kind of tabloid type message, so have the picture here and a message. I think that would be very helpful to a lot of people" [P4, G4 Depression Group]*<br><br>*"I think where NICE have perhaps fallen down, although I think they're getting better at it, is communicating to the public, so people pick up what they get from the media coverage rather than going to NICE's website and reading for themselves what they ought to do" [P4, G7 Communication Professionals]*	
A lack of good quality health information was identified as a concern across the groups. This was a particular concern for people with chronic health conditions and mental health problems. Diagnosis was considered the best time to be referred to a patient clinical guideline.	*"You know I feel as if I had got that there and then it would have been a lot better for me. The guideline should automatically be sent to people who are newly diagnosed; in fact the guidelines should be given to everybody really" [P2, G2 Diabetes]*<br><br>*"I think it's a good idea. I've had experience of people looking at these (patient version) and the feedback I'm getting is amazing and they wondered why they hadn't seen it, the leaflets are not there." [P2, G4 Depression Group]*<br><br>*"I went to my GP...there was absolutely no information at all about mental health, I looked through every single leaflet...You know are you depressed? There's plenty about prostate cancer, cervical cancer all*	Note that there were some participants that felt that there was enough health information available from their GP, but very few.

	sorts of other things that you could pick up...[P1, G4 Depression Group]<br><br>*"Well I just assumed that she [Dr] told me everything I needed to know you see, but she hadn't told me even the half of it..." [P2, G5 Dundee Group]*	
People with a diagnosed health condition value health information very highly. There is a big range in the level of information that a person wants, but everybody indicated that they value information.	*" I, I think, I, I think sometimes it, it depends if you're actually in a situation, and I was recently given some medical news and I then wanted to know absolutely everything, I wanted to read every piece of research, I wanted to look at the impact factor of the journal, how many other people, OK, had cited the work, you know because I, it was something really important and it affected me, so I wanted to know"* [P4, G1 Cervical Cancer]<br><br>*"...we have to give patients access to this I feel we can't let a resource like this go to waste, we're lacking.....and people are isolated and we have to identify a general patient source with solutions and routes and it's important that people are aware of this information source how they can improve the situation for themselves...where can they access information."* [P2, G2 Diabetes Group]<br><br>*"I mean they're, they're, I can almost kind of draw an analogy here wi' em … the building trade if you like in terms of you get electricians, joiners, paperers [ph], and a' these people who spend years learning their, their, and they know everything, and they look at a house and they, they "oh yeah, you could do this and you do that", if you go into B&Q, there's a stand wi' 20 or 30 different leaflets … like this … "how to paper a room", "how to change a plug", how to do this, how to do that, and it's … I mean a, a proper tradesman would look at it and laugh, but I'll be honest wi' yae, if you've never done anything like that before, and you go in and you say "how to change a plug" … "oh here … right, OK …", well it's, "I could do that", and there's pic-pictures on it and it, it's like a starter for 10, and it's always got the warning on the bottom "if you don't feel that you can do this … go to a professional"*	

	… you know so … I mean … there maybe is scope for coming away wi' something that would give somebody a general flavour, for somebody who knows nothing at all…" [NS: G5 Dundee Group]	
Providing good quality patient information does not appear to be a priority for the health service.	*"Alright saying the money's there the money's there [for patient information] but if it's not ring fenced it goes on other things..."* [P2, G2 Diabetes Group]<br><br>*"...we no formal structured patient education provided by NHS [board xx] and when you go to Nicola Sturgeon to say NHS [board xx] are not providing it she says ah but it's in their budget but it's not ring fenced so if they choose to spend money on ipads or whatever instead of structured patient education...the money is not ring fenced."* [P6, G2 Diabetes Group]<br><br>*"It all comes down to finance [the lack of access to patient clinical guidelines]"* [P4, G2 Diabetes Group]<br><br>*"And it also feels like very much, it's a sort of, a, a, it's sort of tacked on to their existing workflows, that there's a, they feel like they've got to produce a patient document somewhere on the thing, so they do it and they put it there, it's not embedded in the process..."* [S4, G7 Communication Professionals]<br><br>*"Well what's kind of frustrating as well is that you know people think, oh anyone, you know it's easy to write a patient version, it's easier than it is to write the doc…actually it's much harder..."* [P3, G7 Communication Professionals]<br><br>*"…as anyone who does that for a living will tell you, you know to explain really complicated concepts, for example around risk, around why some drugs may be suitable for people at a particular stage of their illness, but not at another, you know all of this, it's difficult stuff, and it's thought of as being a free or cheap add-on, and therefore*	Note semi contradictory view in bold, however this person was still saying that you must be assertive and request health information. It appears a consistent view that health information is not routinely supplied even when it is available.<br><br>Note emphasis on financial constraints as the reason for a lack of patient information.

	obviously you don't get a good job." [P3, G7 Communication Professionals]<br><br>*"Very but I haven't seen that quality of information anywhere else about other things and I mean like....I have osteoporosis and to get information like that would help, because it's a charity that sends you stuff, it's not your GP that's given you it."* [P3, G3 Flu Vacc Group]<br><br>**"...eh I have found....we usually got very good information … very good information … if you asked… [P2, G9 Dundee]**	

Theme 1.2: Sources of health information

Findings: 1.2: Sources of health information	Key quotes	Notes
Healthcare professionals were most people's key source of health information. GPs are the most important source for the majority of the public, and specialist nurses are an important source for people with a chronic health condition and vulnerable group's e.g. homeless people.	*"Just from my, my GP surgery... I always go to them, I would, I mean the nurses in the surgery are very good as well...I think they keep you up-to-date, and keep you right"* [P1, G1 Cervical Cancer]<br><br>*"The first contact obviously is your doctor I mean aye..."* [P1, G5 Dundee]<br><br>*"I was just told you have diabetes then the specialist nurse came and started to talk, she gave me information, I couldn't even check a monitor because I couldn't see"* [P2, G2 Diabetes]<br><br>*"I think I would go to the surgery and I would say that I had a concern"* [P2, G3 Flu Vac]<br><br>*"..we all go to the GP when there's something wrong and the GP is, I think, probably the most invaluable healthcare professional we make contact with...if you need support with something they can suggest places to get that.."* [P4, G1 Cervical Cancer]	The GP is particularly important as a source of information due to being most patient's first point of contact with health services and their role in referral. They are the main route to finding out, and accessing the most appropriate treatment.

	"I need to talk to a GP, so the GP, I'm talking simply about my, a GP...obviously the GP can't be a — what would you say? — an expert in this particular field so the GP is dependent on the guidelines that he or she is going to receive, you know, to pass it on to me or, or the patient" [P1, G8 Depression Group]	
Printed text versions of guidelines, accessible directly from health professionals and voluntary organisations, were highly valued. They were particularly important to older people and vulnerable groups who frequently lack access to a computer.	*"When one is diagnosed with diabetes, what should happen is that you should go on a course, an awareness course where you will learn to self-manage and that's when this guidance should be given out." [P1, G2 Diabetes]*<br><br>*"Do you put these [SIGN patient guidelines] in GP offices?" [P7, G4 Depression Group]*<br><br>*"Where would you pick this up from if it was in this form [print leaflet]?" [P10, G8 Dundee Group]*<br><br>*"They should automatically be given to you, if you get diagnosed in hospital or by your doctor, that should be automatic, you shouldn't have to ask." [P2, G2 Diabetes Group]*<br><br>*"if you get these guidelines sorted out, say the consultant is sitting there telling you, you know X, Y, Z and so on, you're often quite… you know … mesmerised by what's going, you know, you know, then would they be able then just to hand you the guidelines? [P1, G9 Dundee Group]*<br><br>*"...it's all very well talking about the internet but there is a lot of depression among older people who do not have access to internet so I mean it is...even more depressing to be told by your GP to go on a website you know that's not ok."* [P1, G4 Depression Group]<br><br>*"Referring people to the internet I think is just the modern way of saying just push off..."* [P5, G4 Depression Group]<br><br>*"The, the, the one problem is, about the Internet is that em, eh many,*	The time of diagnosis was considered the best time to be given a patient clinical guideline.

	many, well quite a substantial number of elderly people don't, just don't seem to have yeah connected the Internet…" [P1, G5 Dundee Group]<br><br>*"How, how would you achieve the volume of stuff that's available, say for eh … Dave, say Dave who doesn't like the Internet mmm, how would you go about getting all that information out..?" [P1, G5 Dundee Group]*	
The internet was a very widely utilised source of health information. The internet was considered a useful source of health information and support. However, it is also an overwhelmingly large repository of health information, which may not be trustworthy. Identifying trusted sites, particularly NHS sites, were highlighted as a way to counteract this.	*"Just talking about the internet, there's a lot more voice in the patient now because of health information, in my context we all use facebook and the likes, we're all on the internet."* [P7, G4 Depression Group]<br><br>"*so the thing I did was go on to look for late onset asthma....That can initially give you so many benefits such as what can be done, what can be causing it, what to avoid, what not to avoid, it's all readily there, and that is the good thing about the internet......secondly eh there was some of them were accredited NHS sites and such like so, common sense tells you if there's a certain accreditation..." [P1, G5 Dundee]*<br><br>*"… I think nowadays when you're, with any sort of diagnosis initially, whether it's going to take you into the blackest hole in the world or not, the internet's the place to go" [P1, G5 Dundee]*<br><br>*"It's like with anything with the web you, you put in "a cut finger," and you end up by being told that you're going to lose your arm" [I1, Homeless person]*<br><br>*"One of my friends has just been diagnosed with MS....And that was horrific with google. So it you want like proper advice and not just something that could be updated by anyone." [P1, G6 Young People]*	All groups mention the internet as a source of health information. Overlaps with other themes.
Older people, and those with chronic health conditions and co-morbid conditions, reported a	*"That this, this means very little to me...[a table extracted from a clinical guideline], eh I'd much have, rather have eh plain speaking than you know from a person, rather than something on a piece of*	General agreement that Apps/QR codes etc are useful, quick and more private in the young people

strong preference for the availability of face-to-face communication with healthcare professionals. Younger people also valued access to health professionals but seemed to particularly value the anonymity, and speed, offered by the internet and Apps.	*paper"* [P2, G5 Dundee Group]<br><br>*"...I mean if I have a, health issue, and I go to see my doctor, I'm inquisitive enough, and my particular doctor is helpful enough to be able to, to have a, a real good two-way discussion and I leave really not needing to go to the Internet…"* [P4, G5 Dundee Group]<br><br>*"I get my…I get the, the children to do all that [accessing the internet]"* [P5, G5 Dundee Group]<br><br>*"yeah like on the bus (mimes looking covertly at phone) rather than a big leaflet."* [P2, G6 Young People]	group.
Voluntary organisations are considered an excellent source of high quality health information in formats that are user friendly.	*"...they [voluntary organisation] would put out information in the magazine and also more specific information to people who run the helpline, so they, they can advise people..."* [P1, G9 Dundee Group]<br><br>*"If you're seeking to empower, you've got to get the information to the patient....that's one of the main functions of Diabetes UK is patient education..."* [P1, G2 Diabetes Group]<br><br>*"...voluntary groups are important, they are vital sources of health information, people there have been there, done that and got the t-shirt, you can use it the way you want and you are talking to people who are in the same boat as you.....it's a good way of getting information about diabetes."* [P2, G2 Diabetes Group]<br><br>*"...Chest Heart and Stroke have a group and there's a lot of people involved with chest heart and stroke things that they can get people right away to answer. And they've got paper they will send out you immediately..." [*P2, G3 Flu Vaccination Group]<br><br>*"I've got the guidelines but the only reason I've got them is because a representative came to one of our support meetings and handed them out. I did not get them from the NHS or any other source."* [P1, G2 Diabetes Group]	

	"...you should go to your doctor and they should say we've got these thingies, [patient clinical guidelines] as you say you don't know what to expect.... We know because we're involved with Diabetes UK Scotland." [P2, G2 Diabetes Group]	
Financial inequalities may affect access to recommended treatment and information	*"…I'm going to skip you out and I'm going to jump the next, I'm going to go and see a consultant...but see...you can do that if you've got money…"* [P1, G5]<br><br>*"...structured exercise programmes [that would put you off if you were]...strapped for cash"* [P10, G8 Dundee Group]	
Lack of access to a computer with internet access makes accessing health information more difficult, groups highlighted as at risk of this disadvantage are older people and homeless people.	*"There's a lot of people in my age group don't [have access to computers]"* [P4, G2 Diabetes Group]<br><br>*"No, my son in-law has got one of those phones, he does all this stuff, I feel exhausted thinking about how to work it."* [P3, G3 Flue Vacc Group]<br><br>*"...it's all very well talking about the internet but there is a lot of depression among older people who do not have access to internet so I mean it is I think even more depressing to be told by your GP to go on a website you know that's not ok."* [P1, G4 Depression Group]<br><br>*"...about the Internet is that em, eh many, many, well quite a substantial number of elderly people don't, just don't seem to have connected the Internet …"* [P1, G5 Dundee Group]<br><br>*"...and also we're dealing with homeless, now how do they access?"* [P2, G4 Depression Group]	
Groups that may suffer from impaired cognition, such as	*"For me this fitted well [SIGN PCGL], I could function to a much higher*	

depressed people, may struggle to process complex health information and be vulnerable to "misinformation" available online	*level even though I was depressed, but for others? Maybe too much to concentrate, I don't know what the answer would be."* [P3, G4 Depression Group]<br><br>*"The internet can be a really dangerous place. I know people have their own agendas, the agenda being I want to get better. So they're looking for this that and the next thing, you know a miracle cure and you're spending money and time and energy and then there's no cure outlook and that just makes you even more depressed because you've failed...I'm very weary, because when you're depressed you don't know which sites are good or bad."* [P4, G4 Depression Group]<br><br>*"...especially if you are feeling depressed and you want it simple enough to follow..."* [P10, G8 Dundee Group]<br><br>*"…in fact someone had made leaflets about half the size and it explained everything about the stroke to you...and it had pictures and, it was quite...it was very help...it was explicit, you could…read it and understand it"* [P2, G9 Dundee Group]	

Theme 1.3: Access to healthcare professionals and treatment

Findings: 1.3: Access to healthcare professionals and treatment	Key quotes	Notes
Variability in the knowledge and communication skills of healthcare professionals, and services offered in a given location, leads to "luck" playing too large a role in access to good health information and referrals.	*"...if you're lucky and you get one who's a good family GP, then they are aware of what's going on in the family as well, so if you need support with something they can suggest places to get that…"* [P4, G1 Cervical Cancer]<br><br>*"The surgery I attend, there is a doctor there who is well clued up and looks after the patients well, but the people just get no information, there are surgeries around who don't have this, it's a requirement by law"* [P4, G2 Diabetes Group]	Participants generally had concerns about variability in healthcare professionals and access to services. Primary care and GPs were most frequently mentioned in this regard.

	"personally I mean if I have a, health issue, and I go to see my doctor, I'm inquisitive enough, and my particular doctor is helpful enough to be able to, to have a, a real good two-way discussion and I leave really not needing to go to the Internet…" [P4, G5 Dundee Group]<br><br>*"some medical care now, or treatments, a lot depends on your postcode...a lot depends on which health area you live in. Patient A might qualify for it, Patient B may not simply because they're living in a different area, under a different healthboard, you know"* [P1, G8 Depression Group]	
Waiting times for an appointment with GPs and limited time with the GP during an appointment is a source of frustration for patients seeking health information. The group of young people indicated that even the 4hr waiting time for a return call from NHS24 is too long for this to be a viable route to health information.	*"...how often can you get an appointment to see your doctor? If your blood sugars are low how long do you wait to see a doctor? It may be two to three days..." [P2, G2 Diabetes Group]*<br><br>*"within the health-eh-care establishments...you need to have an appointment to get information, appointment with ...with some professional and it's, it's being able to get information without having to necessarily make an appointment..."* [P1, G5 Dundee Group]<br><br>*"Yeah but a couple of my friends like have tried to use that [NHS 24] and they'd had to wait ages. For them to phone up like you're on the waiting list and we'll phone you back in like 4 hours and then they go and try something else."* [P2, G6 Young People]	*Communication Professionals suggest people do not like to be given the guidance "consult your GP" in guidelines. This* probably links to difficulties gaining access to HPs and some even the distrust of healthcare Professionals mentioned by some people, even believing information is actively withheld.<br><br>*"… which is basically the guidance is 'talk to your GP'… and people really don't like that …they want "should I have a PSA test - or not?" [P4, G7 Communication Professionals]*
Lack of sufficient time with the GP for discussion of treatment benefits, harms and contraindications was a particular issue for older patients and those with chronic and/or co-morbid	*"...they don't give you enough time to speak about this...you've got a heart problem or a chest problem...I was speaking to my GP...within 7 minutes it was up at the door, right eh, we'll just speak about that next time... I was already anaemic and...I had double pneumonia and chest problems and my heart, right?"* [P2, G3 Flu Vac]	

conditions.	*"...GPs should be capable of em... treating...I've had a few things happening and nobody asks you what the best way is to deal with it, so you've got to just like the flu vaccine you have to decide is this going to be ok for me, especially if you're on a lot medication and as you were saying you might react badly to anything...I think the GPs, I think there's something wrong."* [P2, G3 Flu Vac]<br><br>*"I think I expect a lot of more of GPs than what we receive and the time thing is ridiculous"* [P1, G3 Flu Vac]<br><br>*"you might have something else to ask but their body language you know if they're bent over the computer, you might think that the time is up and you just think oh I'll just go."* [P3, G3 Flu Vac]<br><br>*"...but I think there is a certain cynicism now about treatment, you know people go in for 10 minutes with the most serious illnesses and …they'll only be in there for 10 minutes"* [P1, G5 Dundee Group]<br><br>*"there is a certain GP I have but like I have been to other ones before, if you can't get an appointment and they don't know you at all. Let's say you've got epilepsy and you need to go to a different doctor and they don't know your situation."* [P2, G6 Young People Group]	
People with mental health difficulties highlighted the need for easier access to specialist professionals capable of delivering talking therapies. The waiting times for this access leads to a reliance on voluntary organisations and/or peer support during times of crisis.	*"GPs don't necessarily have the training; you need someone specially trained to help [mental health] patients..."* [P4, G4 Dep Group]<br><br>*"...the CPN was a very delightful woman...but she had not been trained in psychotherapy. She came and I gave her a cup of tea and we had a chat, I mean that is not what is needed in depression."* [P1, G4 Dep Group]<br><br>*"...the ridiculous waiting times, some...takes 18 weeks to see a therapist"* [P4, G4 Dep Group]<br><br>*"How about electing someone as counsellor through a group or similar, so for you your brother or groups. You know forget about*	

	medications or counselling through the NHS." [P1, G4 Dep Group]<br><br>*"… yeah, although they're saying that CBT can be better than discussing with your GP, I mean probably yes, 'cause I mean probably, I mean they're trained in…in that specific field" [I4, Homeless People]*	
People with multi-morbidity are particularly concerned about starting additional treatments because of contraindication, and require more time for discussion with HCPs. They also want health information that is tailored for them and takes account of the complications of having multiple health conditions.	*"But my husband has diabetes and I don't know whether that surgery where everyone is just channelled through for a flu jab, I don't know if they consider whether he has that and if there are any contraindications but he's just one of the crowd and there must be others like that."* [P1, G3 Cervical Cancer Group]<br><br>*"...there's no way that people with epilepsy can take the flu vaccines"* [P4, G3 Cervical Cancer Group]<br><br>*"Now for instance the GP that I go to has 3 stairs up to a room, now I understand, a GP is duty bound to see you downstairs in a room which there is but if you've got a bad bad em let me think. Leg and if you've got a breathing problem, I get there and I'm honestly on my knees and that's fine it's you know oh you're looking fine, you know 7 minutes."* [P2, G3 Cervical Cancer Group]<br><br>*"Another aspect of the question is whether the stronger evidence comes from groups that are more vulnerable and not just age related, or whether it's across the board, because if the stronger evidence comes from em people who have underlying conditions that's quite important to know, it becomes a personal choice as it will help us decide, I don't know it's complicated."* [P1, G3 Cervical Cancer Group]	

Theme 1.4: Interacting with healthcare professionals

Findings 1.4 : Interacting with healthcare professionals	Key quotes	Notes on finding

Most people considered clinical guidelines to be an empowering source of information that could allow patients to ask why specific treatments are not available to them.	*"...when patients go and see the doctor they don't have a reasonably clear picture of what they can expect of the doctor...if there was a guideline which said you were entitled to 12 minutes for example you could say well hang on....it would be helpful to know what we're entitled to, what they are expected to give you as part of their service."* [P3, G3 Flu Vac]<br><br>*"...I think the point about that is that if you didn't know that people thought you should have an MRI scan every two years, you might not get...if you're relying …the GP or the hospital consultant may decide not to provide that, and…you can ask for that..."* [P1, G9 Dundee Group]<br><br>*"if it [health information] gives you the knowledge then it gives you the ammunition to question why you're getting the treatment or why you're not getting treatment"* [P1, G5 Dundee Group]<br><br>*"I think people need to em be aware when they go and see the doctor. I've read through some of the guidelines for mental health, the SIGN ones and I think they're pretty helpful, especially for people who aren't aware of the options that are available to them"* [P7, G4 Depression Group]<br><br>*"yes it might give them more confidence because the clinician can say 'look here is a guideline that you can take home with you' I would say more confidence for thinking 'ok' and ask. If there is more information you can ask"* [P4, G6 Young People Group]<br><br>*"this gentleman...wasn't given the information that he thought he needed until the Internet gave him that opportunity, so it wasn't a case of eh, eh validating the information that was there, it was a case of then you have the opportunity to ask the questions having...gained some knowledge on the Internet." [P1, G5 Dundee Group]*<br><br>*"…on that so we definitely promote and we know people come to us*	

	and have a good awareness of NICE, not just for the decisions but also how can they use it to speak to their healthcare professional and say 'I think I should be getting this' [P6, G7 Communication Professionals]<br><br>*"Em … yeah but I think that might be quite confrontational [taking a patient guideline to an appointment with a GP] and that might be a last resort mmm, I don't think I'd march in and go 'right … yeah, you know this is my right!'..."* [P3, G1 Cervical Cancer Group]<br><br>*"...if I started to suspect that there was something not quite right, he or she [GP] wasn't doing exactly what they should be doing, then I want to go and look at a guideline m-mmm, then I might then go back to them and say 'the guideline says this, why have you not chosen to do that?' m-mmm, and use that as a start of a conversation" [P3, G1 Cervical Cancer Group]*<br><br>*"I was just saying that the words there were choose and choice, the patient chooses to have this facility and it's in the guidelines the doctor would have to have a valid reason for declining to provide the service."* [P5, G2 Diabetes Group]	
Some patients are concerned that asking for specific treatments or discussing clinical guideline recommendations with a healthcare professional could damage their relationship. Therefore it may be best to trust the healthcare professional to make the right treatment decisions.	*"Yeah 'cause you can't go into your GP and say "right, I need that test done", you know…"* [P4, G9 Dundee Group]<br><br>*"Em … yeah but I think that might be quite confrontational and that might be a last resort, I don't think I'd march in and go right …yeah, you know…this is my right"* [P3, G1 Cervical Cancer]<br><br>*"It's quite hard to challenge your GP isn't it?"* [P1, G3 Flu Vacc]<br><br>*"I mean I don't think doctors like when people go along and people say oh I think I've got this or this you know but on the other hand if they did things properly then people wouldn't"* [P3, G3 Flu Vacc]	

	"I think if you do have guidelines which are expanded and they do give you a lot of information then I don't think we should be going into challenge the doctor" [P1, G5 Dundee Group]<br><br>*"...you can't, you've, you've got to go to the persons who's going to direct you and have the contact, you're no' going to have no contact, you can'nae just bypass him and say "right … I don't get on with you", or "you don't give me the information I need, so therefore…" [P1, G5 Dundee Group]*<br><br>*"you can get too much information though. If you get too much then you might, think that their opinion is right and then they might argue against the clinician, who might be doing something for a certain reason and the patient might think that they know their own circumstances better and whoever is treating you can contradict that"* [P1, G6 Young People Group].	
Some patients want to be able to rely on their HCP to prescribe treatment and would only consider accessing patient clinical guidelines if they felt unable to rely on their HCP to provide the appropriate treatment.	*"I don't want to have to check on what GP is doing, I want to be able to trust them…but if I can't, I need to have access to that information [clinical guideline]"* [P3, G1Cervical Cancer]<br><br>*" ...it's like I'd just start assuming the GP's going to do everything right, and I would only start digging into guidelines if I started to suspect that there was something not quite right, he or she wasn't doing exactly what they should be doing, then I want to go and look at a guideline"* [P3, G1Cervical Cancer]<br><br>*"Yes, you feel that the doctor knows best, you would hope the doctor knew best for your own…in a way"* [MC, G9 Dundee]	
People are concerned that HCPs are not passing on good quality health information, such as patient clinical guidelines that outline treatment options, to them. This leads to a lack of trust	*"..the doctors are not always forthcoming as to what's on offer."* [P3, G3 Flu Vacc]<br><br>*"Do you think the doctors don't hand these out because they're not interested in diabetics? Because a lot of them think it's your fault you're fat because you're eating too much? Cut down, sort yourself*	Some people seem to think clinicians have an ulterior motive for withholding health information.

in HCPs.	*out, just think it's your own fault.....I mean that's the impression I get."* [P7, G2 Diabetes Group]<br><br>*"But the likes of flu vaccination, who's going to do that if the doctor doesn't give the information? You should get that from your GP surgery."* [P3, G3 Flu Vacc]<br><br>*"...in case you get a doctor who doesn't want people to know much, and then he writes it in medical terms so it, it completely blinds you" [P2, G5]*	
Some people are surprised at the idea that they would need to know about guidelines, they assume that their HCP implements them.	*"I'm pretty unimpressed to hear that some GPs are not following that guideline, because I don't worry much about guidelines because I assume that my GP does"* [P3, G1 Cervical Cancer Group]<br><br>*"I don't want to have to check on what GP is doing, I want to be able to trust them…but if I can't, I need to have access to that information [clinical guideline]"* [P3, G1Cervical Cancer]<br><br>*" ...it's like I'd just start assuming the GP's going to do everything right, and I would only start digging into guidelines if I started to suspect that there was something not quite right, he or she wasn't doing exactly what they should be doing, then I want to go and look at a guideline"* [P3, G1 Cervical Cancer]<br><br>*"…to a certain extent you do have to rely on professionals making judgements about the strength of evidence, and em you know I can't do everybody's job, at some point you have to trust them"* [P3, G1 Cervical Cancer]<br><br>*" I think I'd only be looking at guidelines if I suspected that my GP wasn't following them, so I wouldn't then be going to the GP for it … I'd be going somewhere else..." [P3, G1 Cervical Cancer]*<br><br>*"But yeah, but then if you were told that you should be getting a letter*	Some people felt that guidelines are only of use to a patient if the HCP (usually GP) is not doing their job.

	and you know that you're not, so GPs are not following the guideline, suddenly then it becomes interesting to me, suddenly then I think, “oh right, maybe I do have to have a look” …" [P3, G1 Cervical Cancer]<br><br>*"Yes, you feel that the doctor knows best, you would hope the doctor knew best for your own … in a way"* [MC, G9 Dundee]	
People that are aware of clinical guidelines generally think that they are just for health professionals.	*"They're aimed at the health professionals, not the [public]…"* [P6, G1 Cervical Cancer Group]<br><br>*"Is it [clinical guidelines] a document the doctors have?"* [P1, G3 Flu Vacc Group]<br><br>*"...the guidelines are also for the doctor to say 'well this is what you should be doing if a patient pops in...'"* [P3, G5 Dundee Group]<br><br>*"...they're two different things aren’t they, I mean if you talk about the NICE guidelines they're really actually guidelines aimed at clinicians, you talk about say the government guidelines they're aimed at the public"* [P1, G7 Communication Professionals Group]<br><br>*"Isn't it for practitioners to get the best evidence of...clinical research and then put it into practice" [P2, G8 Dundee Group]*<br><br>*"...clinical guidelines presumably come from eh the clinicians or drug companies, something like that, I'm not sure that they actually are intended to get down to the level of the patient"* [P1, G9 Dundee Group]<br><br>*"...I thought they were for clinicians so that...know what, how to treat a patient…"* [P5, G9 Dundee Group]	
When patient guidelines are produced they are not always given the same resources as the clinical version, which may result in a lower quality product.	*"..it's sort of tacked on to their existing workflows, .that there's a, they feel like they've got to produce a patient document somewhere on the thing, so they do it and they put it there, it's not embedded in the process"* [S4, G7 Communication Professionals Group]	This is the only quote directly on this, but it fits with other groups thoughts on financial constraints leading to patient information not being a priority.

There is support, from the communication professionals, for plain language versions for patients and clinicians, as opposed to separate versions.	*"Ideally wouldn't it be nice if...guidelines were written so everyone could read them"* [P7, G7 Communication Professionals Group]<br><br>*"...I think it's almost over complicating it to say let's have a doctor version for people who like long words and a patient version for people who don't like long words, why not just have a plain English version of a NICE guideline that anyone can read." [P7, G7 Communication Professionals Group]*<br><br>*"...I don't know if you can have guidelines which are separated from the doctor's guidelines..." [P1, G5 Dundee Group]*	Note that the majority of patients, in contrast, consider the use of medical terminology to make accessing information aimed at professionals impossible. Only one, from the group who work at University of Dundee, mentions looking at research papers.

Theme 2: What patients want to know

Theme 2.1: My treatment choices

Findings: 2.1: My treatment choices	Key quotes	Notes
One size does not fit all - patients want information that enables them to make a choice between treatment options.	*"I think it might depend as well, if it's something that has been maybe hereditary in your family, and even though you are only 20, but your...yes...your mother or your grandmother or something that...you would think well, I want to be tested m-mmm, you know when I'm 20, not when I'm 35 or something"* [P2, G1 Cervical Cancer]<br><br>*"I am really horrified and I mean that, horrified that CBT seems to be the only therapy on offer because it's not a one size fits all"* [P1, G4]<br><br>*"So there are reasons for depression it might be medication [for a physical health problem] that causes depression, might be things in the past so it's not and I'm sorry to say it again a one size fits all solution"* [P1, G4]	Participants made it clear that individual differences, such as family background, co-morbidities and lifestyle all impact on what treatment are right for them - not just effectiveness. Choice means more likely to find something that works for everyone - otherwise excluding anyone that doesn't fit with majority treatment

	"people who have just been diagnosed the opportunity to see what to do and what treatment they should have and what treatment other people have so it might be good for them to say 'this is all the treatment that is available' and then it might be more accessible. And then they can kind of say 'this guideline says this but can I have this' or something, so it gives them all the options" [P2, G6 Young People]<br><br>*"[People want to know] you know "what is MS?", and then "what are the treatments available?"" [P6, G7 Communication Professionals]*<br><br>*"Well it all comes doon tae the auld, auld thing, it may help one person, it dis'nae help person "B"" [P1, G8 Dundee Group]*<br><br>*"Yeah I think it's good that it [patient clinical guideline] gives you the options, you know …" [P5, G8 Dundee Group]*<br><br>*"You got three options there and you know it gives you a choice of either being assessed or sharing your problems with someone but then again some people might not want to do that so the other option is of going through you know a computerised programme for those who are able and all those who just, you know CBT books which again it's giving two good options there that should be accessible to pretty much everyone" [P5, G8 Dundee Group]*<br><br>*"What might be able to help alleviate that problem, whether it be via … change of lifestyle, talking therapy … eh last resort in my opinion would be medication" [I1, Homeless person interview]*<br><br>*"As that lady said, you should give the information to them as choices and let them make up their own minds"* [P7, G2 Diabetes Group]<br><br>*"So that's promoting Warfarin [table from a guideline] … would, would, would there be, can you find out what aspirin alone*." [P1, G9 Dundee]	

	"Yeah I, I, I think the point about that is that if you didn't know that people thought you should have an MRI scan every two years, you might not get, you know you, if you're relying…the GP or the hospital consultant may decide not to provide that, and…you can ask for that..." [P1, G9 Dundee Group]<br><br>*"There's also the fact that as you said there the doctors may choose not to offer this, I don't think that's right. The choice should be the patient's."* [P4, G2 Diabetes Group]	
Patients want to be able to choose to refuse a treatment. Patient clinical guidelines may empower people to refuse treatments that they do not want.	*"So my understanding is if SIGN says that patients should have a choice then the GP would have to have a very good reason for refusing the patient's choice. Because if the patient chooses not to use it then that is their choice."* [P5, G2 Diabetes]<br><br>*"I suppose there's a fear, if you don't have the vaccine you think well if I get the flu that will be my own fault, you know, it's almost like pushing into, I don't know it's a difficult one"* [P2, G3 Flu Vacc]<br><br>*"...if it gives you the knowledge then it gives you the ammunition to question why you're getting the treatment or why you're not getting treatment" [*P1, G5 Dundee Group]<br><br>*"I keep getting drummed into me "it is a choice not to take the treatment", and that should be made clear to the patients as well. With guidelines I'm just wondering does any, has anyone started thinking about "should you be thinking …", you know if, guidelines for clinicians are saying you should offer patients this in this situation, what about the impact on the patients who look at that and think "they're telling me I need to take this", do we need to think a little bit about the choice for the patient, being reflected in that?"* [P7, G7 Communication Professionals]	
Shared decision making is important to patients, but it must	*"...you know you're relying on their expertise but then at the same time*	

be carried out supportively. Patients can feel that they are being asked to make health decisions without the information and support necessary. Having a patient clinical guideline to take away following discussion with a health professional was highlighted as a useful aid to shared decision making.	*they're giving you, they're giving you the option to say “well … mmm, I don't know if I want to take a tablet, could I maybe, what about, you know something else?”* [P7, G1 Cervical Cancer]<br><br>*"...need training for healthcare professionals, it should be a shared decision and on what are patient’s interests? As I say need to be aware of the vaccines and reaction....so I think we need advice"* [P4, G3 Flu Vac]<br><br>*"[sighs] It [Draft patient guideline] … again it's a bit … eh, it's hard to understand, not I think for someone as I say with depression, I mean I, I know when — I'm a bit better now — but when I first … it's like … it's giving you know sort of three or four options to go for, like I mean...you wouldn’t know which way to turn first"* [I4, Homeless Person Interview]<br><br>*"I like the idea of information being able to let people find out what to expect of them but at another level that’s putting a lot of responsibility back on the patient and I still, might be an old fashioned attitude but I still go to see my GP and in my mind and although it’s a GP I’m going to see the specialist, I don’t want to be the one making decisions, because what do I know about the medical background of things?"* [P1, G3 Flu Vacc]<br><br>*"to have to go and do your own research, it’s putting so much pressure on patients, I had a friend, this is a slight deviation but she had cancer and in terms of treatment she was given loads of information and asked to make her decision. She said to me why are they putting it back on me?" [P1, G3 Flu Vacc]*<br><br>*"Yeah I appreciate the shared bits but this ladies experience was that it was very much for her to decide and you can see the staying away from compensation culture type but it makes it very hard for someone who is unwell."* [P1, G3 Flu Vacc]	

	"I think it's an additional burden, I know friends who have had cancer treatment, there are real big differences between some of the options, you know radiotherapy, chemotherapy top-ups after surgery, things like that, and these are big decisions? For people and at the end of the day you know fine for the doctor to say here are the options but most people would like the doctor to...well, direct you to the right option. I had a friend who said what if it was your wife? And the guy did give her an indication, probably a lot of them wouldn't but I mean I know it's a good thing patient choice but I think for a lot of people it's a burden and I think the time constraint, if they could talk with you for say 15 minutes to explore all the options rather than go away and think about it, what do you want?" [P3, G3 Flu Vacc]<br><br>*"…See you could discuss some o' this stuff with your doctor…he could give it to you in a fashion that eh would move away from the kind of horrors that you might get if you read it yourself"* [P4, G5 Dundee group]<br><br>*"..if you go away with paperwork...people who are unable to absorb that information diminish their lifespan by 5 years research had shown so we know the GP becomes much more critical when we think about it like that because how many of us have listened to someone explaining something and when asked if you've understood you say yes? And you have at them time but you go away and...how much do you take in...?" [P1, G3 Flu Vac]*<br><br>*"The doctor has to use his or her judgement as to whether it [a recommended treatment] would be useful for the individual."* [P1, G2 Diabetes Group]	

Theme 2.2: Harms and lifestyle

Findings: 2.2: Harms and lifestyle	Key quotes	Notes
Information on harms is	*"I mean some antidepressants cause weight gain, some people really*	

necessary for patients to make a treatment choice. They must be able to judge whether the benefits of a treatment outweigh the risk.	*get fat and they find that very distressing, it makes you even more depressed so people need to be made aware." [P1, G4 Depression Group]*<br><br>*"Well I think for flu vaccination how likely are you to feel unwell. Our technician took the flu vaccination and was in bed unwell for 4 days whereas I didn't have it and took the flu and was in bed for 5 days. So he was more unwell from taking the vaccine than I was from flu so it's things like that you know if you thought you were likely to be poorly for 3 days or something, if you could quantify it like that you know."* [P3, G3 Flu Vacc Group]<br><br>*"Well I was simply given medication for 5 years and I think it made me worse not better, the side effects of medication included panic attacks in supermarkets, constipation, I won't go into details and oh yes libido just vanished but nothing else was offered to me except the visit to the CPN..."* [P1, G4 Depression Group]<br><br>*"I know that if I am taking tablets I read all the information. I don't know why but I want to."* [P3, G6 Young People]<br><br>*"Well that phrase "risks outweigh benefits", I think that rings true for everyone"* [P10, G7 Communication Professionals]<br><br>*"...there was an operation offered which involved cutting eh up the side o' the head, and eh very close to the artery that goes up to the brain, and eh after talking to the, the medics about the matter, eh wi' any procedure as we've, as I found out there's a downside … "what's the downside?", the downside wi' that particular op is … it's so close, it's so minutely close to the brain, you can finish up in a wheelchair …So the patient then has to sit down and decide … "what are we going to do?" "Is it worth it?""* [P2, G9 Dundee Group]<br><br>*"I like the bit "what are the benefits and harms?", yeah that's quite good, it's something that you would be interested to read a bit further*	

	wouldn't it?" [P4, G9 Dundee Group]<br><br>"*…this many people benefit, but there is, this many people may have a serious side-effects or something"* [S2, Communication Professionals]<br><br>*"…I've heard that the, at some stage that, all of us we should take an aspirin every day, a lose dosage, I've also heard that aspirin can 'cause cancer…"* [P1, G9 Dundee Group]<br><br>*"...so you've got to just like the flu vaccine you have to decide is this going to be ok for me, especially if you're on a lot medication and as you were saying you might react badly to anything you are given...I think the GPs, I think there's something wrong" [P2, G3 Flu Vac]*<br><br>*"it [patient clinical guidelines] gives me eh a balanced view, whether it's going to be good for me or not, or whether the, the bleeds worse" [NR, G5 Dundee]*	
People expressed concern regarding the lack of information on harms, or even minimisation of potential harms by health professionals.	*"I've gone round to the pharmacy and asked as it says in leaflet not to drive or something but the doctor never said anything about that and I mean I'll sit and go through the packet inserts and it doesn't say in here so is that based on something else? The GP should tell you all this before you read it on the paper because you know if you're not allowed to drive that has a huge impact on people's lifestyle and are they just advising everyone to avoid alcohol or is here a really good reason like if you take it you'll have a .... or whatever, I think it's really important to know if people have understood."* [P3, G3 Flu Vacc Group]<br><br>*"Yeah 'cause your doctor never tells you, you've got to read…the leaflets, the doctor never says"* [P4, G9 Dundee]<br><br>*"...but eh she [mother] went into hospital for a major operation, at least it was major as far as she and myself were concerned, but eh they,*	

	they thought little of it, and eh, but they did, told me nothing about how she would be afterwards…" [P2, G5 Dundee Group]<br><br>*"the doctor eh wanted to em give me an operation, and I, I looked it up on the Internet, what, what it, what he was going to do and the consequences. He didn't tell me about the consequences right of what he was going to do"* [P2, G5 Dundee Group]<br><br>*"...quite frankly Warfarin is a, is a rat poison, you know and they make it sound here [table from clinical guideline] like it's, it's sugar pills mmm, you know and I would want to know you know that it's, it's very, it meets very major screening to basically make sure that you don't kill your patient with it" [P3, G5 Dundee Group]*<br><br>*"I, I'm quite interested in the fact that, does it say Warfarin doesn't, doesn't em 'cause any major lifestyle changes… yeah whereas people I know have been on it just hate …you know hate being on it because of the lifestyle … implication" [P1, G9 Dundee Group]*<br><br>*"…always have a a side-effect inside their boxes, and the doctors don't believe you that they do. I have terrible nightmares with some of the pills I'm on and scare me to pieces...Or when you say to the doctors about the side-effects they just pooh-pooh, or "don't be ridiculous", you know and therefore you, you carry on..." [P10, G8 Dundee Group]*	

Theme 2.3: What to expect – causes, symptoms and outcomes

Findings: 2.3: What to expect – causes, symptoms and outcomes	Key quotes	Notes
People most require good quality health information at the time of diagnosis. They can become frustrated and angry if they do not	*"I, I think, I, I think sometimes it, it depends if you're actually in a situation, and I was recently given some medical news and I then wanted to know absolutely everything, I wanted to read every piece of research, I wanted to look at the impact factor of the journal, how*	

receive it	*many other people OK had cited the work, you know because I, it was something really important and it affected me, so I wanted to know..."* [P4, G1 Cervical Cancer Group]<br><br>*"P3: And he was newly diagnosed and he came along to our meeting.*<br><br>*P4: ....sparks coming out his ears...he was furious that he hadn't got information"* [G2 Diabetes Group]<br><br>*"I just woke up in hospital and was told I had Type 1 diabetes. I didn't know anything about the illness"* [P2, G2 Diabetes Group]	
People are eager to know about all the possible outcomes from a treatment and what to expect from services and recovery	*"Yes, they are super, he had about 6 of them, one for investigative procedures and one for surgery and then you know after care, they were very useful with pictures, explained everything, they really did, they went into everything and addressed things well you know if you wanted to know what happened afterwards. I felt if you wanted to know something they were useful." [P3, G3 Flu Vacc Group]*<br><br>*"My brother in-law recently had surgery for a hernia operation which failed by keyhole surgery so he went back for the bigger operation and em when he was discharged from hospital you would think someone would sit him down and say we'll do this and we'll do that but nobody I know has had this process and literally as he was going out the door he said to a nurse what should I be doing? He had to ask, he had to take the initiative."* [P3, G3 Flu Vacc Group]<br><br>*"When my father had to have this operation he was told that there was a 1 in 5 chance that he would survive the operation and what actually happened was he came through the operation but didn't come round properly and he was in intensive care, really it would have been much better if he hadn't had the operation, it would have been better to continue with what he had until it was catastrophic. We felt, it sounded like they were laying their cards on the table at the time, about the specifics but what they didn't tell us was what would happen if he*	Also supported by quotes from other themes and groups.

	survived the operation but had all these other complications and if there was a 1 in 3 chance of that you would think well the chance of it going well is 2 in 3 and we would use this to make our decision but that wasn't something we knew, no-one ever mentioned it, nobody prepared us. No-body told you about the risk of stroke or any of these things that could happen but it wasn't until he came out of intensive care that someone actually told us what had happened." [P3, G3 Flu Vacc Group]	
People want to know what to expect from the condition they have - its symptoms, treatments, and realistic outcomes.	*"It shouldn't be..... We don't know what we're entitled to............we and our families are living with this illness, we should know what to expect..."* [P2, G2 Diabetes Group]<br><br>*"Yes, you should go to your doctor and they should say we've got these thingies [patient clinical guidelines], this is what you..... As you say you don't know what to expect...."* [P2, G2 Diabetes Group]<br><br>*"That's something that tends to be glossed over by the medical profession, they don't tell you that if your blood sugar is above a certain level for ten years then you can end up blind....there's a 40% chance of blindness, losing a leg, a foot or whatever, it's scary."* [P1, G2 Diabetes Group]<br><br>*"People feel they are not expected to all of a sudden not have this condition, that they are not suddenly going to get better, therefore there must be an awareness that it's not you that's letting people down by not getting better it takes time and somewhere along that line you have to get that message or you as a patient will become more depressed."* [P2, G4 Depression Group]<br><br>*"the broad brush effect that you go and you say "you have cancer, here's what you have", you know "you have a small cell carcinoma", or whatever you have, eh and all the chances of dealing with it, or what treatments can be going down"* [P1, G5 Dundee Group]	

	"you know "what is MS?", and then "what are the treatments available?" [P7, G7 Communication Professionals Group]<br><br>*"Signs and symptoms"* [P5, G8 Dundee Group]<br><br>*"It told you what to expect… but this little book [patient information leaflet produced by a voluntary organisation]…told you everything, and you would read it and you'd think "oh, that's what happens"… [P3, G9 Dundee Group]*<br><br>*"And [patient information leaflet] even told the carers what to expect...[P4, G9 Dundee Group]*	
Clinical care pathways are a way to help patients know what to expect from their condition and its treatment.	*"…there is one for MS [a patient clinical guideline] and it does cover the broader things rather than the technology appraisals, and it's also about care pathways…much more broadly and so someone's who's living with a long-term condition sees that possibly far more useful than just simply wanting the answer they might get…from the media"* [P6, G7 Communication Professionals Group]<br><br>*"I guess also then you could also, in your guideline, it could also be stratified about where you are in your disease progression, particularly if...you might want to make different decisions"* [P10, G7 Communication Professionals Group]	Note that they are managed by name only by the communication professionals however they fit what is being described as lacking by the patient groups.
People did want to know about possible causes. However this was not mentioned frequently.	*"What brought it on"* [P1, G8 Dundee Depression Group]<br><br>*"...what you thinks' brought it on, you know, it could be a combination of factors, couldn't it?...Most people I think eh it's either health or money or eh marital problems, wi' most people...*[P1, G8 Dundee Depression Group]<br><br>*"M-m-m … whether or not, firstly whether or not I was in the risk group"* [I1, Homeless People Interview]	Causes are not as widely mentioned as treatments, outcomes, risks and symptoms.

	"Em, basically the causes…and symptoms" [I4, Homeless People Interview]	

Theme 2.4: Self-management

Findings: 2.4: Self-management	Key quotes	Notes
Patient clinical guidelines ability to enable self management is a priority for patients	*"How to help yourself...Help yourself, yes, I think that's the top priority [for patient clinical guidelines]"* [P3, G2 Diabetes Group]<br><br>*"No I think you need to monitor, you want to.... If you want to sort out your diet etc you need to know what you're doing or what you're not doing. I do mine morning and night and sometimes in between times."* [P6, G2 Diabetes Group]<br><br>*"...it's important that people are aware of this information source [patient clinical guidelines] how they can improve the situation for themselves..." [P2, G4 Depression Group]*<br><br>*"health promotion and easy things to do that are part of your life. [shows me a leaflet] so instead of saying, 'raise your blood sugar' you would say 'go and eat a mars bar' Rather than something about blood glucose which you don't..." [P4, G6 Young People Group]*<br><br>*"Things that you can do yourself to help" [P6, G8 Dundee Depression Group]*<br><br>*"And I would have thought the majority of people would rather go down the, the more natural or more … you know things that they can maybe sustain just in every day to day life approach, in terms of … in terms of sort of doing the diet and the…exercise and things like that first and possibly the, the nutritional supplements or medicine would come at a later stage" [P5, G8 Dundee Depression Group]*<br><br>*"What might be able to help alleviate that problem, whether it be via …*	

	change of lifestyle, talking therapy … eh last resort in my opinion would be medication" [I1, Homeless People Interviews]<br><br>*"I like the idea of an emergency plan, for anyone that takes the vaccine, I'm in good health but maybe I should have some information so that if I have a bad reaction to it what should I do?" [P1, G3 Flu Vacc]*	
Patient guidelines, and any form of good quality health information, enable self management and reduce the need for input from health services.	"When one is diagnosed with diabetes, what should happen is that you should go on a course, an awareness course where you will learn to self-manage and that's when this guidance [patient clinical guideline] should be given out." [P1, G2 Diabetes Group]<br><br>*"Don't forget the more information we get, the better we can look after ourselves. If we have these guidelines, you see him [GP] less..."* [P2, G2 Diabetes Group]<br><br>*"If you've got these guidelines, it saves time, it saves money. In the long term if we know what we're entitled to and can look after ourselves and have our questions answered, we will visit the doctor less because we can look after ourselves."* [P2, G2 Diabetes Group]<br><br>*"I get the test strips..... A Type 2 diabetic going to his doctor....if something goes wrong and blood sugars are too high, it's another eleven months before it's checked again, it's wrong, in that eleven months an awful lot of damage can be done"* [P4, G2 Diabetes Group]	Patients would actually like to have more contact with their healthcare professionals but given the infrequency of contact self management is even more important. The diabetic group are keen to highlight this point; they were particularly concerned by a lack of access to patient guidelines and withdrawal of funding for blood sugar monitoring.
One size does not fit all - some patients may be unable to make use of interventions aimed at self-management or require more support to do so.	*"Wouldn't it depend on the ability of the patient to use the results meaningfully? The doctor has to use his or her judgement as to whether it would be useful for the individual."* [P1, G2 Diabetes Group]<br><br>*"Giving out monitors and test strips without the education on what patients should do with the results isn't helpful, it could be a problem. It has to be given out with information on what are the consequences of not keeping control." [P1, G2 Diabetes Group]*	

	"I was lucky when I was diagnosed, I've got a medical background I trained as a nurse, so it wasn't as big an ordeal when it came to diet, I knew that I had to cut out this that and the next thing, what I could have or substitute, the likes of milk, changing sugar for sweeteners, like change from butter to flora, I had a good idea of what I had to do." [P3, G2 Diabetes Group]<br><br>*"yeah cause I have personal experience of it and the strips like only give you a number and you would need like a leaflet or something to tell you what the number is. It's not 'low' or ' high ' or something you would need to have some explanation."* [C, G6 Young People Group]	
Some people linked taking responsibility for your own lifestyle with the ability to self manage your health condion(s)	*"I like the bit about self-management and a', 'cause see at the end of the day … it dis'nae matter how many professional are helping you, at the end of the day you need tae dae it yourself and it's basically down to you, it's like, it's like being an alcoholic or you're a heavy smoker or something and you're wanting tae quite, there's only so much the National Health Service can dae for yae, it's really doon tae you at the end of the day yeah anyway, you know"* [I5, Homeless People Interviews]<br><br>*"…Now my opinion is that we know our own body to an extent and we know if we feel good or if we don't feel so good, and you can sit doon and analyse and say "well what am I doing wrong?", or "what should I be doing that I'm not doing?", and eh I think personally that's as good a, that is a, a very good guideline, as far as I'm concerned" [P2, G9 Dundee Group]*<br><br>*"Researcher: 'So you're saying this woman wasn't informed, she wasn't aware? Do you think if she was given a patient version of the guideline, do you think that would that have been helpful?'*<br><br>*P4: 'Yes'*<br><br>*P3: 'If she was prepared to accept it! It's their attitude too'*	

	R: 'So attitude would play a role in the acceptance of the guidance on self help?'<br><br>*P7: 'Yes*<br><br>[G2 Diabetes Group]	

Theme 2.5: Signposting

Findings: 2.5 Signposting	Key quotes	Notes on finding
People want information that refers them to appropriate sources of support and places to access treatment.	*"...so if you need support with something they [referring to GP with access to a clinical guideline] can suggest places to get that."* [P4, G1 Cervical Cancer Group]<br><br>*"Where you should go. It's not a case of being spoon-fed because we're all adults but if you're suffering from this or have the signs of this, this is where you can go for help? We need to know that"* [P6, G2 Diabetes Group]<br><br>*"Em where I could get help"* [I4, Homeless Person Interviews]<br><br>*"...sometimes it annoys you because if you have a specialist diabetic nurse, they should be saying there is a group of people [Diabetes voluntary organisations] who can help you. It took about a year and a half before I found that out." [P2, G2 Diabetes Group]*	People are angry at lack of good health information and once they "find" voluntary organisations that provide it they are angry that they were not signposted to them earlier.
It is not adequate to only provide websites when signposting an organisation, some groups, particularly older people, require services that can be accessed by phone or dropping in.	*"The other thing is it's all very well talking about the internet but there is a lot of depression among older people who do not have access to internet so I mean it is I think even more depressing to be told by your GP to go on a website you know that's not ok."* [P1, G4 Depression Group]<br><br>*"Referring people to the internet I think is just the modern way of*	See themes reflecting need for print information for older people and vulnerable groups also.

	saying just push off." [P5, G4 Depression Group]	

<u>**Overarching theme 2: The Guideline**</u>

<u>**Theme 3: Properties of guidelines**</u>

Theme 3.1: Language and tone

Findings 3.1: Language and tone	Key quotes	Notes on finding
Keep language simple - minimise the use of medical and technical language, keep sentences and paragraphs short, and avoid ambiguity.	*"Yeah 'cause as, as long as it's got, if it's easy to read and not too medical or, or technical"* [P7, G1 Cervical Cancer Group]<br><br>*"Simple, yeah simple…you know straightforward, you know "this is why we're doing this"* [P7, G1 Cervical Cancer Group]<br><br>*"...it's got to be in lay terms, that the simplest person can understand" [P3, G2 Diabetes Group]*<br><br>*"As long as it's in a language that we can understand" [P4, G2 Diabetes Group]*<br><br>*"it got to be in words people can understand, non-jargon, it's got to be almost black and white...it's like the information from Diabetes UK, here's the fifteen checks that you should be getting from your GP....it's how you get the information through, how you communicate..." [P5, G2 Diabetes Group]*<br><br>*"it's not exactly jargon but if it's medical terminology that maybe needs to be simplified" [P1, G3 Cervical Cancer Group]*<br><br>*"...there was a lot of medical jargon...I know from experience and you know congratulations for producing this booklet [SIGN patient guideline] but patient themselves cannot all read it and therefore you*	There are many similar comments, they have not all been extracted here.

	have to produce something shorter and simpler. [P2, G4 Depression Group]<br><br>*"Could I just say that this menu of psychological therapies is very frightening. What exactly is behavioural activation? Mindfulness based cognitive behavioural therapy? You know these words frighten me, what do they mean?" [P1, G4 Depression Group]*<br><br>*"… it's what I call medical language… anthro., eh anti … thro., thromotic treatment, well you know…if to us that means how to stop thrombosis, then why don't they say so?" [P2, G5 Dundee Group]*<br><br>*"...I think it means it doesn't affect your daily lifestyle as much, low burden means it's not eh a 'burden' [laughs]…well, well is it not meaning a low burden, low burden on the National Health Service, because these people will tick along without… [P1, G5 Dundee Group]*<br><br>*"...so instead of saying, 'raise your blood sugar' you would say 'go and eat a mars bar' Rather than something about blood glucose which you don't [understand]."* [D, Young People Group]<br><br>*"I don't like the word "intervention", 'cause what does that mean?"* [P10, G8 Dundee Group]<br><br>*"'Structured Exercise Programme'… people might think what's "structured"....structured exercise programmes like whatever...What is it?"* P10, G8 Dundee Group]<br><br>*"it has the title Allograft Transplantation, it's quite a complicated title, but it's aimed at patients, I thought that's great, you know if people ask us about that then I can send them this document, but I'd be amazed if they found it..."* [S5, Communication Prof Group]<br><br>*"...the management or Type 1 diabetes in children and adolescents, you know I can't imagine that patients would be uninterested in it, if*	

	they came across it, but I think they'd probably come to Diabetes UK and get our "What Care To Expect" document" [S5, Communication Prof Group]<br><br>*"…if you don't mix with people I suppose the maintenance of social networks...'Personal meaningful activities'…what is that?"* [P10, G8 Dundee Group]<br><br>*"...if you just have shorter sentences and where you've got a long word, if, if you make it into a, if, if you can possibly break it into say a slightly different thing, you can reduce the reading age of something quite considerably and it's much more helpful, because if people are confronted with long sentences and technical terms they...maybe don't get past that first introduction…"* [P1, G9 Dundee Group]	
Medical terminology is identified as the main reason that patients and clinicians cannot use the same guideline	*"...there is a NICE guideline, we'll go and look at the guideline to see OK, what will you expect from treatment, but we do find that we have to basically translate it"* [P7, G7 Communication Prof Group]<br><br>*"…they [Patient and clinicians version of a guideline] should be the same, but they should be basically different language"* [P1, G5 Dundee Group]<br><br>*"...clinician's are much more technical so there is going to be a few words that a clinician would know but Joe Bloggs's wouldn't. You're looking at having two separate ones, for clinicians and for patients..."* [P3, G6 Young People Group]<br><br>*"...the health people will be like legal people they, they want to keep a' the fancy words and that because it is, it is an exact meaning to them...and if you open it up to make the ordinary man on the street try and understand it you'll probably lose a bit of the definition of the meaning..."* [P1, G5 Dundee Group]<br><br>*"Ideally wouldn't it be nice if [there were like? 13.00] guidelines were*	

	written so everyone could read them" [P7, G7 Communication Prof Group]	
The use of language that people do not understand can lead to distrust of the information and the developers of the information.	*"...why do certain groups of people have to communicate in a way that's really difficult to understand? I don't know what the vested interest in you know policy-makers communicating in acronyms all the time...you know it just makes no sense to me"* [S8, G7 Communication Professionals Group]<br><br>*"…throwing in a piece of information that is....I used to work for a, an organisation who form the working for Bills that go through parliament, and I know for a fact that they deliberately word them like they do so that you have to employ a solicitor"* [P2, G5 Dundee Group]<br><br>*"...in case you get a doctor who doesn't want people to know much, and then he writes it in medical terms so it, it completely blinds you" [P2, G5 Dundee Group]*<br><br>*"...then there becomes an issue of language, because if you're making a guideline for a GP…or a consultant clinician, the language that you use will be …very different, and a layman may not be able to…understand the language..." [P1, G9 Dundee Group]*	
Avoid a negative or dogmatic tone. People expressed a preference for the tone of writing to be as positive as possible, particularly when mentioning a lack of evidence for a treatment. However this was balanced by the need to convey factual information in a straight forward manner.	*" I think the only problem is it's starting off with a negative…i.e. "there is no evidence …", it's starting off with a negative which is the wrong approach"* [P3, G8 Dundee Group]<br><br>*"Be positive"* [P10, G8 Dundee Group]<br><br>*"I was going to say that's quite unhelpful 'there is no good evidence'…right away you're, again you're going into the negative"* [P3, G8 Dundee Group]<br><br>*"I would rather have said 'some people have found the health bonus of herbal remedies'…and just start off like that, you know … so say what is possible rather than what isn't possible"* [P3, G8 Dundee Group]	

	"…the question's got to be asked and it's got to be answered and it's quite straightforward … and you can't really dress it up can you?" [P10, G8 Dundee Group]<br><br>*"…'cause the rest of it's quite positive, 'yeah – give it a try', but we'll tell you that there's no evidence"* [P10, G8 Dundee Group]<br><br><br>*"…this seems to be slightly negative — the way it's portrayed [likelihood of weight loss following an obesity intervention]…you may only, might lose, might lose a bit less. I know you have to cover all eventualities but maybe slightly more in a positive … obviously you can't use the words 'you will'…"* [I1, Homeless People Interviews]<br><br>*"it also depends [on the way of] giving the information, the ways that [they] put it over you know if they say dogmatically you must to this you must do that"* [P3, G2 Diabetes Group]	

Theme 3.2: Meaningfulness

Findings 3.2: Meaningfulness	Key quotes	Notes on finding
Health information becomes meaningful and highly valued if it refers to a condition that you or someone you care for has been diagnosed with.	*"...it depends if you're actually in a situation, and I was recently given some medical news and I then wanted to know absolutely everything, I wanted to read every piece of research, I wanted to look at the impact factor of the journal, how many other people had cited the work, you know because...it was something really important and it affected me, so I wanted to know"* [P4, G1 Cervical Cancer Group]<br><br>*"It's extremely general...think if you were in that situation it might mean a bit more to you, because you'd be looking for what applied to you"* [P4, G5 Dundee Group]	This may influence the results of this analysis which has sometimes asked people to comment on health information on conditions that they do not have.
Patient involvement in research and guideline development will	*"that is not patients as guinea pigs but patients as members of research teams to say ask the right questions in the first place and*	

increase the meaningfulness of the guideline to the patient. Chiefly by ensuring that the research itself and the outcomes reported have meaningful to patients.	*what is the impact assessment of having people butting in on trial steering committees and so on and I think it is absolutely crucial that this is done, I mean how many patients were on the steering committee of the trials that they are quoting for CBT? I mean you know you've got to have the patient perspective at the beginning of the research otherwise you might be looking at the wrong end of the telescope."* [P1, G4 Depression Group]<br><br>*"Patient input is quite new and in the guidelines that I'm involved with now I continually hear we can't produce guidelines where there isn't research and what patient groups are saying and what people experience, and therefore somewhere along you cannot get approved research this is going to be missed, we do have to highlight the things that are very important to patients"* [P2, G4 Depression Group]<br><br>*"This is a new thing to have patient input and there is very good work..."* [P2, G4 Depression Group]<br><br>*"Yeah because often clinicians might measure outcomes but then the, the things that they think are important are not the things that the patients think are important [are actually suffering for this disease that's why I think it's important that they do go to Patient Groups when they're doing their guidelines m-mmm, because patients will soon tell you, you know what matters to them about coping with their condition I think"* [P3, G1 Cervical Cancer]	
If information presented to people does not appear to be aimed at 'someone like me' it will usually be rejected. Therefore health information needs to be tailored to the audience as much as possible.	*"...this [a table] doesn't seem to apply to patients because it says does it apply to your patient which suggests just to me that it's aimed at the doctor, and for the doctor I think it's dumbed down too much…"* [P3, G5 Dundee]	Related to the belief and appearance of clinical guidelines as just for health professionals. Also use of language and tone that is excessively medical. The extremely negative reaction of many people to the use of very prominent QR codes on some

		draft materials may be related to a feeling that they are not for them if using a technology they do not understand.
Ensure the focus of the information is on what the patient wants to know and not technical information. While the communication professionals suggested that information on how a guideline is produced will help patients to understand guidelines. Most patients indicated that they are interested in this information, but it should not be made too prominent as it can distract from what they have come to the guideline to find out. Others were not interested in it at all.	*"...something that would helpful would be...telling the story of how the guidance came, because...stories are what people react to most, so like this is what's happened, they met on this, they decided this, they looked at this data, they came to this [decision]"* [P4, G7 Communication Prof Group]<br><br>*"...that's very important in general about communicating science, kind of opening this black box that all of this seems to go inside, that you don't talk about how all of this worked out, you just talk about the end product"* [P2, G7 Communication Prof Group]<br><br>*"I would kick off with point number one 'What Is Obesity?', because that's where you're going to start off, 'How The Guidelines Have Been Produced', could be down at the bottom…"* [I1, Homeless People Interviews]<br><br>*"There's no point in teaching somebody, or getting somebody to look at how the guidelines are produced when they're actually wanting to find out "What Is Obesity?""* [I1, Homeless People Interviews]<br><br>*"...the doctors are going to advise the patients on the information in guidelines that they...have received, so the origin of the guidelines is coming from where?"* [P1, G8 Dundee Group]<br><br>*"'Cause if you thought you were overweight you're not worried about how the information has been got...think it needs to be there…but not in that order"* [I1, Homeless People Interviews]<br><br>*"Yeah I think you need to know [what a guideline is] before [reading the guideline]"* [I3, Homeless People Interviews	Not all groups were shown full patient clinical guidelines with materials on guideline production so reflected in the limited range of groups represented in these quotes.

Theme 3.3: Trustworthiness and credibility

Findings 3.3: Trustworthiness and Credibility	Key quotes	Notes on finding
The source of guidelines credibility seems to be based on the understanding that they are the result of a thorough review of the available evidence.	*"...it gives it more weight if it's based on a guideline...because you assume you know even...if you don't understand exactly what a "guideline" means, but it gives it some em, as I say "weight" that it's based on some evidence from somewhere that this is a good thing to do, someone's looked at some evidence and made a guideline from that" [P4, G1 Cervical Cancer Group]*<br><br>*"You would hope that whoever produced the guideline would have looked at...the research, so it synthesising a lot of different information, and making a valued judgement about that, that we think there is sufficient convincing evidence..."* [P4, G1 Cervical Cancer Group]<br><br>*"...SIGN decides that this actual activity should be included in the guideline, then that's met the criteria, the evidence backs it being made a guideline..." [P5, G2 Diabetes Group]*<br><br>*"…some of it [information from the internet] might actually be dated, whereas presumably the guidelines that we're looking at will be, should be the very latest information..."* [P1, G5 Dundee Group]<br><br>*"...the guidelines..."you have a small cell carcinoma"...what treatments can be...I think it has to be before anything goes on, it has to be validated, there's little point in having it without that...I think they have to be reliable."* [P1, G5 Dundee Group]	Although this was not discussed by many groups, it was suggested as the source of the guidelines credibility by all that did discuss credibility or reliability of recommendations.
Guidelines do not have to be implemented and this can undermine their credibility and meaningfulness for patients.	*"...guidance is a guidance, it's not a rule or a law, so senior staff can say there's the money we'll spend it there, not on people with diabetes..."* [P6, G2 Diabetes Group]<br><br>*"...the guideline, the doctor doesn't necessarily have to follow it…[they can] Keep the patient in the dark and…decide what happens"* [AJ, G9	

	Dundee Group]	
An apparent conflict of interest in the producers of health information and research can undermine credibility	*"I went to Victoria retinal screening a couple of months ago, there were leaflets in the waiting area but they were all from drug companies. They're all trying to sell blood glucose monitors." [P1, G2 Diabetes Group]*<br><br>*"How are you going to get the evidence if you don't have the research and some research isn't sexy or backed by the drug companies" [P1, G4 Depression Group]*	
Changes to recommendations can threaten the credibility of a guideline. The reason for a change should be described in an understandable way but in considerable depth.	*"The other thing though if they change the guidelines...I think they should explain, lots of people will say how come? Why are they now saying that we should get it..."* [P3, G3 Flu Vacc Group]<br><br>*"I think they need to do a better job explaining why...you can explain it, we're not stupid, if there's some good reason for changing the guideline then let's hear what it is, it should be able to stand up to scrutiny."* [P3, G3 Flu Vacc Group]<br><br>*"...go to work on an egg...and then they're very bad for [you]"* [P2, G9 Dundee Group]<br><br>*"The thing is that there's so many variables and over time even, even what seems to come occasionally from the medical people, seems to vary, I'm talking in terms of the...optimum, or, or the maximum alcohol intake per week, that seems to fluctuate eh from year to year, em so you know how can you enshrine something like that into stone?"* [P1, G5 Dundee Group]	
Health information, produced by the NHS is considered trustworthy and reliable. Patients will look for health information marked as NHS produced.	*"...there is a lot of information out there...you get some really whacky sites. I know when...you needed advice they liked you to look at NHS."* [P3, G3 Flu Vacc Group]<br><br>*"We think if we pick up a leaflet in Ninewells [Hospital], that we think*	

	it's going to be safe, it's an NHS leaflet..." [P1, G5 Dundee Group]<br><br>*"...some of them were accredited NHS sites...so common sense tells you there's a certain accreditation" [P1, G5 Dundee Group]*<br><br>*"NHS [sites are] helpful, useful"* [I4, Homeless People Interviews]	
The credibility and trustworthiness of guidelines is harmed by the public perception that strong evidence for effectiveness is ignored if the costs of a treatment are high.	*"...one of the things that I have a problem with the guidelines...say 'well we want to know how strong the evidence is', well sometimes...the evidence may be strong but it's just expensive, we're not doing it anyway, and that's where guidelines also fail...'cause then you're not getting it, even if it's good."* [P3, G5 Dundee Group]	Note this is discussed under costs and cost effectiveness but does impact trust in guidelines so is left here as well.

Theme 3.4: Guidelines as restrictors of access to treatment

Findings 3.4: Guidelines can restrict access to treatment	Key quotes	Notes on finding
Guidelines recommendations can prevent access to some preferred treatments. Patients are also concerned about the potential for inappropriate referrals to treatments with a stronger evidence base than options that may suit them better.	*"It's the guidelines, I used to get strips, type 2, I don't get them now because according to doctor I don't need to, I've got it under control but to me I don' t have it far enough down but I don't get the test strips to do it."* [P6, G2 Diabetes Group]<br><br>*"Well I had heard of NICE and SMC but you tend to hear more about what they do, given publicity when they don't recommend a particular drug or whatever for some cancer treatment or something like that, I don't know that I would have connected them to guidelines for flu..em...vaccination."* [P3, G3 Flu Vacc Group]<br><br>*"Exactly but you started off with CBT and CBT is the one that doctors know about but it's not the only solution, and I think that this idea that because there is evidence then you've got to refer people to CBT, I think it's really really dangerous to be perfectly honest." [P1, G4 Depression Group]*	The communication professionals were the only group that appeared to be aware that guidelines recommend more treatments then they do not recommend.

	"I am really horrified and I mean that, horrified that CBT seems to be the only therapy on offer because it's not a one size fits all" [P1, G4 Depression Group]<br><br>*"...what you need more than anything [when you are depressed] is someone to talk to who's not going to judge...what's even worse is that people with depression should be referred to computer counselling on the CBT model....the only reason that there's evidence...is because it's been researched because it's quite easy to research because it's highly structured..." [P1, G4 Depression Group]*<br><br>*"I mean the problem I have with guidelines [is, or 'cause?] usually, it says "well you have disease A, and this is what we're going to do" and what I would like in a guideline is "well this is what we should be doing in 98% of the cases, but there might be those 2% where this would be better", and I do miss the kind of alternatives. I mean that's, that's what the whole thing is about so he comes and says "well, you know you should have an operation", and you think "well is there any other way"…"* [PR, G5 Dundee Group]<br><br>*"I mean this is quite advanced [SIGN patient guideline] and we want something like that as well but nothing about evidence based you know forget about that because it's not true just because you don't have the evidence doesn't mean it doesn't help, and that kind of jargon is not helpful if you are in the depths of depression."* [P1, G4 Depression Group]	
People are concerned that complex interventions are more difficult to research and therefore may never acquire the evidence necessary to be recommended in a guideline. People fear this leading to a restriction in access	*"...this evidence based I mean it begs the question if you see what I mean and I'm using this in the real sense and not in sense that one hears every day the question is what kind of counselling or talking therapy is most effective? Now if you only ask the question about one form of counselling then you're not going to get a proper answer. That is not evidence. CBT happens to be have been very widely researched a because a)someone in America is promoting it and b)*	This may lead to a disconnect between what people experience in the community and what the guidelines recommend, which is confusing for the patient. Note that this concern is coming from the more informed patient groups

to complex intervention that are not gaining funding for research or are hard to manualise.	*it's easy to research because it's more like a tick box exercise and more fluid person centred type therapy so no wonder there's evidence for that but you know it's very difficult to get funding for research particularly if you're researching a talking therapy which isn't very fashionable at the moment..."* [P1, G4 Depression Group]<br><br>*"...the absence of evidence does not mean evidence of absence, you know just because there hasn't been research in a whole lot of other therapies, the most widely used type of psychotherapy is person-centred...and has been very effective for many people even if the evidence base is not quite as hard as CBT simply because it hasn't been possible to do research due to funding problems."* [P1, G4 Depression Group]<br><br>*"'... have a slightly concern over the flagging up, so blatantly the evidence-base...for chronic conditions, for things like physiotherapy…with MS and other conditions...it may be quite disheartening or undermine their chances to access that kind of service…and while I'm all for evidence-based, I'm not sure there's ever going to be an evidence-base for many things…things that just aren't easy to trial…"* [P6, G7 Communication Prof Group]	from voluntary organisations etc, who are aware of a range of treatments.
Reasons behind not recommending an intervention in a guideline is generally seen as cost based rather than effectiveness based.	*"That was a cost cutting exercise, it's to save money, it's about money not the illness, they have to cut back. As you say they say Type 2 don't need to monitor"* [P2, G2 Diabetes Group]<br><br>*"The GP might choose not to offer it but it doesn't say why, and that could come into the whole rationing thing. You know I've only got so much of a budget and I can't give out everything. And that will let me out of it. But if it gives reasons why they've not got a machine then that would explain to people rather than 'oh they're not giving me this, they haven't got money for it.' But if it like gave reasons then people would understand."* [B, Young Peoples Group]<br><br>*"…yeah but I wonder where cost comes...in decision making"* [P1, G9	Note this is coded elsewhere under trustworthiness and costs and cost effectiveness. However it also relates to restrictions to treatment, since the majority seem to belief that not recommending a treatment is purely an exercise in cutting costs.

	Dundee Group]	
In contrast to the patient groups, the communication professionals considered NICE, in particular, to be objective and a gold standard in guideline methodology. The misperception of NICE, by the public, could be addressed by a more sophisticated media management strategy.	*"...until something's actually gone through NICE or the equivalent then it's not really a guideline it's an organisations point of view"* [S4,G7 Communication Prof Group]<br><br>*"...NICE panels ...they're so hot on their Conflict of Interest and people literally have to leave the room if they've got anything vaguely associated, it's not that they can just say 'well…my Disclosure of Interest' and then the people will take it with a pinch of salt, they literally have to leave the room…"* [S1,G7 Communication Prof Group]<br><br>*"I think it's an absolute crying shame that NICE have the reputation they do and the way the public, because I think everyone thinks, well not everyone, but everyone here sort of seems to be agreement that the job they do is pretty much invaluable…"* [P4, G7 Communication Prof Group]<br><br>*"it [NICE] was so respected internationally…we don't appreciate it but everyone round the world is envious"* [S1,G7 Communication Prof Group]<br><br>*"I think that NICE do a really good job...what frustrates me is...the campaign is always around 'NICE has got it wrong, we need to change NICE's mind' and I always want to...say 'well…why is no one asking why this drug is so expensive?'"* [P7, G7 Communication Prof Group]<br><br>*"...it's about the way they present it so they, they have a poor media management of their decisions…"* [P1, G7 Communication Prof Group]<br><br>*"...with the Abiraterone decision the other week, we very clearly said in all our statements - 'firstly we want Janssen to lower the price', and 'secondly we want NICE to use the End of Life guidelines', the thing the media picked up on was 'NICE got the wrong guidelines'* [P4, G7	

	Communication Prof Group]<br><br>*"They actually approve more cancer drugs than anything else, you know it's just…they're, they're more positive than negative, you know that comes up, but you wouldn't know that from reading [about NICE]…"* [P8, G7 Communication Prof Group]	

Theme 4: Presenting the evidence

Theme 4.1: Numbers

Findings: 4.1 Numbers	Key quotes	Notes on finding
Some people find information in a numerical format a helpful and direct way to communicate health information. While others are likely to disengage when presented with complex numerical information.	*"...it [table of numerical information on Warfarin] gives eh an indication of what would work, and what may not work, it gives that indication right away...It's very brief...well I found it helpful..." [NR, G5 Dundee Group]*<br><br>*"…I mean I, I would want to see something concrete … numerical if you like…"* [P1, G9 Dundee Group]<br><br>*"No I would rather just see words [instead of chart] …like 'so and so per cent did this, and so and so per cent did that'*…[P5, G9 Dundee Group]<br><br>*"...what we need is it'll be... sort of pictorial representation, some sort of…simplified eh description of the problem...really enabling me to have more clarity...because first glance that's going to put me off [table of numerical information on Warfarin]...*" [P6, G5 Dundee Group]	
Presenting outcomes in a numerical format gives the information credibility	*"...I think if you see something written down like that...there's a tendency to take that as the gospel…because it says there 'prevent 9 non-fatal strokes'... and you accept that..."* [P4, G5 Dundee Group]	
Numerical information can be dehumanising.	*"...that is an easier way to do it, there's no getting away from it, I mean a, a broke, breakdown like that [numerical information on prostate screening]...well my problem is that dehumanises…when you're talking about thousands..."* [NR, G5 Dundee Group]	Personal stories are highlighted by the groups as a way to make health information more engaging and relatable. Note that only one

		person states this directly but all groups find the materials presented can be difficult to engage with and dry, and the majority favour personal stories as a way to make it more relatable.
People find it very difficult to interpret relative risk without information on baseline/absolute risk. The communication professionals found this particularly frustrating.	*"What frustrates us...when we get, for instance press releases and often when we drill down to the paper it's very difficult to, they'll say something...[about how] it improves or reduces your risk of whatever cancer by 5% ...but they never talk about...the risk that they're going up from…"* [P1, G7 Communication Professionals Group]<br><br>*"…they don't talk about the baseline risk and, so often it's quite an effort to pick out what the actual real improvement or greater risk that they're giving you is, you know you're 5% risk is, is nothing, and we do try quite hard to put that into our pieces but sometimes it's very hard to get hold of and we have to explain it quite heavily and I think the public because it doesn't get "fronted up" enough, it's quite hard for us then to communicate that to the public...you know the journalists in this area would like it generally to be pushed out there more so the public understood that more…"* [P1, G7 Communication Professionals Group]<br><br>*"…I e-mailed the Press Officer saying "this means nothing" [without] absolute risk, and I wish everybody would do that...[if] It's not there at all, it's really annoying"* [P7, G7 Communication Professionals Group]<br><br>*"...[Someone is] developing an on-line tool that you can put relative risks into that will give you the absolute figures, which is think will be one of the most amazing things that's ever…"* [P4, G7 Communication Professionals Group]	Note only the communication professionals discuss this issue directly but the other groups did find complex numerical information challenging to interpret without contextual information. The simpler the better.
The communication professionals strongly supported the use of frequencies, and whole numbers, in reporting numbers to patient	*"...we tried to put it down to if a 1,000 people take aspirin for this many years then this many of them are likely to get cancer, if a 1,000 people don't, then this many are likely to get cancer…this many people out of 10, or 100, or a 1,000, instead of percentages, percentages are hard*	

groups.	*to think about"* [P8, G7 Communication Professionals Group]<br><br>*"they...explained why they don't routinely do scanning, so they showed how many people would be misdiagnosed if you did scanning, and then went on to say we think with this trial, what we know so far is that actually it'll be much lower number of people" [P10, G7 Communication Professionals Group]*<br><br>*"...in numbers rather than percentages … so if this many people do this, then…this many people benefit, but there is, this many people may have a serious side-effects..."* [P8, G7 Communication Professionals Group]	
Many people are interested to hear the personal stories of others and find them helpful.	*"...I'm quite a logical thinker...but I might be interested in stories..."* [P1, G3 Flu Vacc Group]<br><br>*"Chest heart and stroke they've got a good leaflet...it's got cases on people who have developed things and you know how they're getting on you know but it give you, sometimes you say oh that's interesting, so it just shows you."* [P2, G3 Flu Vacc Group]<br><br>*"I particularly found helpful some of the organisations dealing with mental health issues like Bipolar Scotland who has various case studies. I don't know if they are evidence based but it's a good resource anyway for access to information" [P7, G4 Depression Group]*<br><br>*"...testimonies of patients who have been through the experience…I think that would be an excellent idea…"* [P2, G5 Dundee Group]<br><br>*"I think that [use of personal stories in guidelines] would be good"* [P10, G8 Dundee Group]	In general groups were in favour of personal stories, although some people actively did not find them helpful.
Personal stories can help a person to understand what their treatment choices are and to	*"...personal experiences....that kind of thing sort o' encourage you and it helps you to decide what options you had"* [P7, G1 Cervical Cancer Group]	

choose between them.	*"...they're giving advice again aren't they, they've been through it, if you're listening to somebody's personal opinion…"* [P4, G9 Dundee Group[]	
Personal stories may help to humanise/personalise the evidence presented in patient guidelines.	*"...that is an easier way to do it [graphical representation of prostate cancer screening outcomes], there's no getting away from it...breakdown like that …well my problem is that dehumanises...when you're talking about thousands there then it's very difficult to..."* [P1, G5 Dundee Group]<br><br>*"I think personal stories are valuable because you know it's a, a genuinely real case study and...people can relate to that …rather than just...people saying there's no evidence and...people can relate to the personal stories a bit more..."* [P3, G8 Dundee Group]	
Personal stories can help a person to not feel alone with their health condition.	*"...when you get some headline talking about a celeb, drugs for depression help and I think that's very helpful, it's comforting to know that somebody who apparently has everything actually has depression...you know Stephen Fry is the obvious example so and so has depression so that means it's not just me...I think that would be very helpful to a lot of people."* [P4, G4 Depression Group]<br><br>*"…you can listen to Mr Smith or Mrs Brown's story, how does it relate to my own story…"* [P1, G8 Dundee Group]<br><br>*"...somebody might, might say something and you think 'oh, that's exactly what I was thinking', or feeling you know…you can relate to it…"* [P4, G9 Dundee Group]	
Personal stories can be alienating if the reader does not recognise themselves in the story. Personal stories should be balanced and represent different possible outcomes from treatment.	[She personally doesn't like narratives or stories as she finds them alienating. If she, or her patients, don't recognise the scenario they may feel worse or feel that they are not responding as they should.]<br><br>*"...where are you going to get all the information...you know it's going to be sporadic...the information, you can't have everybody saying, giving their little story…"* [P1, G5 Dundee Group]	bracketed quote is from an unrecorded telephone conversation between researcher MC and a person who could not make the depression focus group. It is in a note form so cannot provide direct quotations.

	"…we could get both sides then [from personal stories]…" [P2, G5 Dundee Group]<br><br>*"You'd have to have more than one, you'd have to have a position, a negative and…somebody in between, yeah…"* [P1, G9 Dundee Group]	
Some people actively do not want to hear others personal stories, and would rather have factual information presented to them.	*"Probably the numbers and information for me [instead of personal stories]"* [P5, G9 Dundee Group]<br><br>*"You don't want to hear somebody's personal view…that's been through something, no?* [P4, G9 Dundee Group]<br><br>*No...no"* [P3, G9 Dundee Group]<br><br>"...*stories would be all right on an app but they would take up too much room on a leaflet. Yeah if something like while your app was loading it came up with a wee story that would be all right but not on a leaflet."* [P4, G6 Young People Group]	It does seem like this is a minority group, but it should be noted that they do not appeal to all people. Other point is that they are not a priority for many people and would not necessarily be a good use of space in a print format

Theme 4.2: Charts

Findings 4.2: Charts	Key quotes	Notes on finding
Simple charts were appreciated for their ability to convey a substantial amount of information in a visually attractive format.	*"I like graphs, I like pictorial stuff…because you can get an immediate analysis of something..."* [P1, G5 Dundee Group]<br><br>*"...I think graphs can get over an awful of information … in a much quicker period, it's a picture isn't it, a graphs a picture, and a picture's worth a thousand words. If you try to read everything you had in that graph…… so if you can come up with pictures and graphs initially, eh whether it's a graph as C said or a pie chart or a, a block graph, it can quickly get over stuff"* [NR,G5 Dundee Group]<br><br>*"...as I say you can get all that information from graphs, straight away, as soon as you look at a graph, they can give you a hundred graphs if*	Bar chart shown to G8 was simple and well liked, all the more complex charts were disliked and found difficult to interpret

	you want about all different em symptoms or whatever, and from the graph you can pick out a lot of information from that, and it's only a glance you need, you're not having to sit and digest it and look at words you don't understand…" [P1, G5 Dundee Group]<br><br>*"…tables and stuff like that in the midst o' text are very attractive, to me, when I open, when I turn a page, even though the table or the graph on, two pages on is not relevant to what I'm reading at the moment, but it's something relevant half a page on, you turn the page, you, your…eyes automatically it goes to that…"* [P1, G5 Dundee Group]<br><br>*"....graphs are handy …You've got the, the numbers and the visual … the graph itself...that catches your eye right away…"* [P1, G8 Dundee Group]<br><br>*"It takes you straight to the graph [bar chart] first before the reading at the top...it makes you want to read at the top after that..."* [P10, G8 Dundee Group]<br><br>*"the graph's [bar chart] quite good though, it's...very informative and it's very simple to look at and simple to follow and simple to read, you know so it's good, aye"* [P1, G8 Dundee Group]<br><br>*"I'd maybe do bar charts rather than that [complex weight loss intervention chart], a bar chart across might, might make a difference, or a pie chart even..."* [I3, Homeless People Interviews]<br><br>*"I think graphs are easy to understand … again as long as it's not too much information then I think a graph is good..."* [I4, Homeless People Interviews]	
A chart needs to be accompanied with a text explanation of the data presented.	*"No I would rather just see words [instead of a chart]…like 'so and so per cent did this, and so and so per cent did that'"*… [P5, G9 Dundee Group]	One size doesn't fit all. Some people prefer information presented graphically and others prefer text.

	"Yeah I think you need...I would like both really [chart and text explanation], 'cause I think some people…need the visual…to confirm you know what they're reading...sometimes...you know the reading can be quite difficult…" [P1, G9 Dundee Group]<br><br>*"Yeah I can see, that the, to be honest the narrative is doing more… than the actual graph is… so I mean you could nearly do away with that and just use the narrative"* [I3, Homeless People Interviews]	
Complex charts/graphics were confusing for most people, if the meaning is not apparent at first glance most people will ignore the chart/graphic.	*"I find it hard to read [Prostate cancer screening chart]...and I'm not fond of the puzzles either..."* [P3, G5 Dundee Group]<br><br>*"…I don't like it [Prostate cancer screening chart], it doesn't make an instant visual…"* [P1, G5 Dundee Group]<br><br>*"...I personally will look at graphs and, and, and pie charts and things, but if I saw a...this [Prostate cancer screening chart] I, I just would'nae …I'd just scoot, scoot over it"* [P5, G5 Dundee Group]<br><br>*"...that's [Prostate cancer screening chart] showing the numbers that were … treated unsuccessfully…but I, I just can't [understand it]"* … [P1, G9 Dundee Group]<br><br>*"...they...wouldn't want to sit down and then read through it [complex weight loss intervention chart]...no"* [I1, Homeless People Interviews]<br><br>*"...if you look at it very carefully, you know that's what will happen...if you're on the programme but it doesn't … you know without reading it, it doesn't leap out at you and say…I mean obviously that's a decline in...weight...but it doesn't register that straight away … I don't know if that's just me..."* [I3, Homeless People Interviews]	
The communication professionals thought that online graphics are very useful to communicate	*"...represent a risk of benefits graphically using people...it was amazing, I really like that"* [P11, G7 Communication Professionals Group]	However as noted elsewhere many people do not have access to online information.

evidence.	*"So graphics really work but you do need to remember that not everyone is able to see them..."* [P4, G7 Communication Professionals Group]	

Theme 4.3: Costs and cost-effectiveness

Findings 4.3: Costs and cost effectiveness	Key quotes	Notes on finding
Indicating that the evidence for an intervention is weak, or making a recommendation against an intervention, is considered to be about reducing costs and not about effectiveness	*"That was a cost cutting exercise [recommendation that type 2 diabetics do not need to self monitor their blood sugar], it's to save money, it's about money not the illness, they have to cut back. As you say they say Type 2 don't need to monitor"* [P2, G2 Diabetes Group]<br><br>*"It all [recommendation that type 2 diabetics do not need to self monitor their blood sugar] comes down to finance"* [P4, Diabetes Group]<br><br>*"The GP might choose not to offer it but it doesn't say why, and that could come into the whole rationing thing. You know I've only got so much of a budget and I can't give out everything. And that will let me out of it. But if it gives reasons why they've not got a machine then that would explain to people rather than 'oh they're not giving me this, they haven't got money for it.' But if it like gave reasons then people would understand."* [B, Young Peoples Group]<br><br>*"…yeah but I wonder where cost comes...in decision making" [*P1, G9 Dundee Group]<br><br>*"...they won't recommend some drugs, on the basis of cost-effectiveness when they can prolong someone's life ...they are … em authorising some treatments and not others, and I feel… [it is about] money"* [P4, G1 Cervical Cancer Group]<br><br>*"I think, I mean I think there is a perception of rationing."* [P3, G3 Flu Vac Group]	Note this point is made in several other themes also.

	"If there was like only weak evidence to eh, to, to get a screening, what would you think of that?" [Researcher]<br><br>*"Money"* [P4, G1 Cervical Cancer Group]<br><br>*"Well because you, you hear of, you, you know, they, they won't recommend some drugs, on the basis of cost-effectiveness when they can prolong someone's life or you know there's, there's one recently em that helps people with em macular degeneration problems and you think, well you know we're spending, they are … em authorising some treatments and not others…"* [P4, G1 Cervical Cancer Group]	
Some people did think that recommendations are based on costs and effectiveness, and even support the role of costs in making treatment recommendations.	*"I think because it costs to have a screen, then will GPs say well we're not going to do that, because it costs money and there's no evidence, because these guidelines I believe are, you know based on cost effectiveness, as well as things like false positives and whatever, so I mean the case for breast screening I think is mixed because, you know there, there's evidence that, that it throws up a lot of false positives...with all the, you know chaos and cost that produces, but I think if clinicians are given a choice about whether to do something or not, they will look at how cost effective it is"* [P4, G1 Cervical Cancer Group]<br><br>*"Well it's so important in everything, the cost effectiveness, it had to be … what would you say … one of the major brickworks o' the whole show. If the thing's no viable because it's too expensive it's just not going to work, sooner or later it's going to close the doors, so it's so important right and the more cost effective it can be and still let's say bringing home the goods then that's great, that's a bonus, aye"* [P1, G8 Dundee Group]	Note that this perspective is rare and most people, aside from the communication professionals, considered any lack of support for a treatment to be about financial constraints.
Most People did ***not*** want to be given information on cost effectiveness. Any information on costs and cost effectiveness was	*"No, I don't think [I want to know about cost effectiveness]…I think there's a cynicism...that many people feel as though treatments are now cost-based"* [P1, G5 Dundee Group]	Note the point on language here in the quote from I1. Could this information on costs be given in a more sensitive way, using a more

associated with rationing, and denial of access to services.	*"Yeah and that's one of the things that I have a problem with the guidelines as well, when you say "well we want to know how strong the evidence is", well sometimes you know the evidence may be strong but it's just expensive, we're not doing it anyway, and that's where guidelines also fail, you know 'cause then you're not getting it, even if it's good"* [P3, G5 Dundee Group]<br><br>*"No, no what I mean is, if someone's depressed then they don't care do they?...they just want help, they don't care how much it costs…"* [P10, G8 Dundee Group]<br><br>*"…because of the cost-effective approach … I think there would be a better way of actually delivering that information saying that it would be more beneficial to you rather than using the words 'cost-effective,' 'cause people then think … 'ooh, part of a money-saving government scheme,' then push people down the route of CBT"* [I1, Homeless People Interviews]<br><br>*"I would do, I would leave it [cost effectiveness information] all out…"* [I1, Homeless People Interviews]<br><br>*"I don't think you need that information [on cost-effectiveness] because I think it's…that's down to the, the NHS...it's not down to the patient..."* [I4, Homeless People Interviews]	friendly tone and language?
Providing a full rationale that Peoples can understand, for recommendations against a treatment, may stop people from assuming that such a decision is purely cost based.	*"The GP might choose not to offer it but it doesn't say why, and that could come into the whole rationing thing. You know I've only got so much of a budget and I can't give out everything. And that will let me out of it. But if it gives reasons why they've not got a machine then that would explain to people rather than 'oh they're not giving me this, they haven't got money for it.' But if it like gave reasons then people would understand."* [P2, G6 Young Peoples Group]	
The communication professionals think that it would be more	*"The information on the cost is often buried in the guidelines so you have to like, you're talking about trawling through to find it so if that*	The communication professionals group was in agreement with this

effective to make information on costs more accessible in a guideline, so that the underlying rationale behind not recommending a treatment is more evident.	*was [easier to find] that would be far more effective"* [P10, G7 Communication Prof Group]	point. This is an interesting conflict with the patient groups that generally do not want to know about costs and perceive all such information as negative. This is difficult to balance with them wanting a clear, easy to understand rationale for why something is, or is not, recommended.
Computerised Cognitive Behavioural Therapy (CCBT) was perceived as a treatment option intended purely to save money.	*"...save money but, I think…it [Computerised Cognitive Behavioural Therapy]...I mean it would, it would save money but I don't think it's cost-effective because it's not practical…I mean doing something on-line, it's just like talk., talking to somebody on Facebook or on email or, and it's, it's very impersonal"* [I4, Homeless People Interviews]<br><br>*"...what's even worse is that people with depression should be referred to computer counselling on the CBT model."* [P1, G4 Depression Group]	It was considered an unhelpful treatment option despite evidence supporting its effectiveness.

Theme 4.4: Levels of evidence & recommendations

Findings 4.4: Levels of evidence & recommendations	Key quotes	Notes on finding
The term weak recommendation was unclear in meaning, and perceived as negative in tone.	*"[a weak recommendation means that] there's not enough evidence for blood tests?"* [P6, G2 Diabetes Group]<br><br>*"You mean [by weak recommendation] they were half hearted about it?...maybe 51% of doctors thought it was a good idea and 49% thought it wasn't."* [P3, G3 Flu Vac Group]<br><br>*"I think weak means em..em..em. they're not sure, they have doubts about it"* [P4, G3 Flu Vac Group]	Note that all these interpretations are guesses - it is not evident what is meant by the term itself

	"I would interpret that as maybe that it should only be for people with particular conditions?" [P1, G3 Flu Vac Group]<br><br>*"I think it means it's the person's right to not have it, for whatever reason it's up to them...Most people should decide if they do want it."* [P2, G3 Flu Vac Group]<br><br>*"A weak recommendation is a recommendation that was more or less on the right line"* [P2, G4 Depression Group]<br><br>*" [Weak evidence means] there wasn't enough information to support."* [P4, G6 Young People Group]<br><br>*"It's [Weak evidence] maybe like the backing behind it isn't that strong. It could either be that or like there is some reasoning behind it but it's quite weak or it could be like it's not very direct. Instead of being really direct, strong, straight to the point, it's kind of like round the houses."* [P2, G6 Young People Group]<br><br>*"it's not really the best direction that it would give the patient or the clinician. There could be other more successful things."* [P1, G6 Young People Group]<br><br>*"It can really confuse people...what it meant so if you saw "weak" they would think that it didn't work very well, not that the evidence was "weak", they'd think "oh it has a weak effect"* [P7, G7 Communication Professionals Group]<br><br>*"But that's it, recommendation is, is as loaded a word as, as 'weak'…"* [P8, G7 Communication Prof Group]	
People generally have a good understanding of what strong evidence means.	*"I would say it was like...it's been proven that … that, that evidence … you know someone's done some hard research...and it's been proven"* [P7, G1 Cervical Cancer Group]	People seemed to assume that all evidence used to make a recommendation would (or should) be strong.

	"You would hope that whoever produced the guideline would have looked at a-l-l the research, so it synthesising a lot of different information, and making a valued judgement about that, that we think there is sufficient convincing evidence…" [P4, G1 Cervical Cancer Group]<br><br>*"Well strong evidence is eh, eh … what would you say, you're going to accept that a lot easier…"* [P2, G9 Dundee Group]	
The majority of people did not like the idea of a weak recommendation which was based on weak evidence. They did not want weak recommendations included in patient guidelines. However, people do want to know about the rationale underlying a recommendation, and could feel misled if the strength of the evidence is not revealed.	*"I wouldn't want to hear it [a recommendation] was weak...well that recommendation for flu vaccine, I would take it with a pinch of salt if it's based on weak evidence and actually I would think what are they doing producing a recommendation if it's not strongly endorsed."* [P3, G3 Flu Vacc Group]<br><br>*"I think that would be rubbish to say that. We recommend it but it's no based on strong evidence, I think it's a rubbish statement."* [P1, G3 Flu Vacc Group]<br><br>*"So, so you would want to know even if there was weak evidence for something?" [researcher]*<br><br>*"Yep"* [J]<br><br>*"Yeah"* [K]<br><br>*"I think you need to be honest, we take it for granted that there's a guideline recommending that over 65s should have the vaccine and the evidence is that it will benefit you and I think people will think there is strong evidence so I think it's being economical with the truth, you know we're not giving you the full story."* [P3, G3 Flu Vacc Group]<br><br>*"If we say to patients we've got a weak recommendation no I don't think a weak recommendation is good...it's confusing...I think guidelines are a voice and in a situation when you need quick*	People are surprised that any treatment might be based on weak evidence. It is both the term "weak" and the notion of weak evidence that is disliked.<br><br>However, the idea that a recommendation should not be applied in a blanket way to all people is well supported by the patients. These groups understood a weak recommendation as something based on weak evidence.

	information to go through, [a weak recommendation] is confusing for me...you want to put an answer in front of them" [P2, G4 Depression Group]<br><br>*"If there isn't any evidence it suggests to me that there's not much point in issuing that [a weak recommendation or one based on weak evidence]"* [P3, G4 Depression Group]<br><br>*"It's [a weak recommendation or one based on weak evidence] discouraging, it requires more clarity"* [P3, G4 Depression Group]<br><br>*"one size doesn't fit all so it would be absolutely wrong to say the evidence was weak."* [P4, G4 Depression Group]	
Most people did not understand why there would be uncertainty around a treatments effectiveness.	*"Going back to a criteria, if SIGN decides that this actual activity should be included in the guideline, then that's met the criteria, the evidence backs it being made a guideline, if there was no evidence to support that why would you put it into the guidelines in the first place. So if it's proven that having blood sugar monitoring is a pointless exercise then you wouldn't have it in the guidelines, if it's been proven to be beneficial to patients then it goes in the guideline because it's met the criteria."* [P5, G2 Diabetes Group]<br><br>*"I don't know how that would work if you could say to people we're giving you something we don't have enough research for."* [P2, G6 Young People Group]<br><br>*"It's kind of contradicting itself by saying that…yeah "there's no evidence"…and then it says "there [could be] much to be gained from…yeah that's contradictory isn't it?"* [P10, G8 Dundee]	Note that what people do understand is treatments being effective for some groups of people and not others e.g. Co-morbidity, age, contraindications. Weak evidence/recommendation was seen as equivalent with no evidence.
The term moderate evidence was found unhelpful and vague by most people. People require an explanation of the underlying evidence to make sense of the	*"I think that's [term moderate evidence] not specific enough"* [P3, G5 Dundee Group]<br><br>*"I think the moderate thing, it makes it, basically what you're reading there of little value at all…"* [P1, G5 Dundee Group]	

meaning of 'moderate quality evidence'"	*"…so it doesn't, it, really it means nothing…'moderate'… moderate to whom?...you know what's moderate to you…"* [P1, G5 Dundee Group]<br><br>*"But you need a lot more information than just eh a part of a sentence to tell you that information, they can't tell you that in three or four words…"* [P1, G5 Dundee Group]<br><br>*"where does it come from…'who, who judged that?'…you know I could say it's moderate, or I could say it's great"* [P3, G5 Dundee Group]	
Indication that the evidence for a treatment is weak, or moderate, may not have a strong influence on a person's intention to access a treatment.	*"It's like what you were saying earlier about everyone's different, you know maybe some people...wouldn't be put off by that [weak evidence], they would still anyway whereas other people might think "no I can't go to that", so…I don't think it sways you either way"* [P10, G8 Dundee Group]<br><br>*"Once again it [draft patient guideline] says 'there is no good evidence'...it doesn't put me off, but it makes you think … well I'll try myself, you know…"* [P10, G8 Dundee Group]<br><br>*"Depends how desperate you are, doesn't it?...If it's a 50/50 chance and you're desperate, you might go with it…"* [P5, G9 Dundee Group]<br><br>*"[If the evidence for a treatment was weak or moderate] You'd probably think twice wouldn't you?"* [P5, G9 Dundee Group]	
Avoiding the use of terms such as weak and instead giving a more thorough explanation of the reasons for uncertainty was considered the best approach to explaining evidence levels to people.	*"well if you were to kind of word it like 'it's worked for so many people and it might work for you' yes it's worked but it's not worked for everyone...you should emphasise the positive. So like saying it has worked but they haven't had enough time or enough people to try it on. 'We think it works and it's worked in this this and this situation and we have this amount of people who've tried it but we need this amount to say for sure.'"* [P3, G6 Young People Group]<br><br>*"...it's not one word, it's probably about 40, but it, it's something along*	Note a similar point about changes to guideline recommendations. Support for a relatively brief and understandable rationale for each recommendation is strong.

	the lines of…'we don't expect new evidence to change our conclusion about this treatment' or 'we do expect…'" [P7, G7 Communication Professionals Group]<br><br>*"Don't duck it. If it is uncertain, say so…you know patients don't like it, but it's better than pretending"* [P7, G7 Communication Professionals Group]<br><br>*"...take your time to explain that uncertainty properly…and don't, don't take any shortcuts on uncertainty, and whatever else you do"* [S7, G7 Communication Professionals Group]<br><br>*"...back that up with the story as well, why, 'why is it uncertain', I think, break it down"* [P6, G7 Communication Professionals Group]<br><br>*"It would be more helpful for me to say something like em research is ongoing..em.. and evidence is not, it's very fluid."* [P3, G4 Depression Group]<br><br>*"I think in terms of the recommendations, weak recommendations I think that's fine as long as we have the other psychological therapies here as a sort of offcast. I've said before as long as you say there's no hard evidence however some people might find them helpful then that's ok."* [P7, G4 Depression Group]<br><br>*"We reverted to putting it in the text [having tried a star rating system], so we would write something about, you know … "the evidence shows this works for X out of 10 people", or whatever, "… however, the evidence is not very strong", or "the evidence is uncertain … we can't rely on the results", something like that to actually explain it in words"* [P7, G7 Communication Prof Group]	
Patient information should have the least amount of ambiguity possible.	*"The options need to be black and white...If the patient has the options in black and white...that would certainly be very valuable."* [P3, G2 Diabetes Group]	

	"Don't think introducing doubt [into recommendations] is a good thing" [P1, G2 Diabetes Group]<br><br>*"One piece of advice [about producing patient guidelines] I would say is, try to avoid ambiguity..."* [P1, G5 Dundee Group]<br><br>*"…where...the guidance is "talk to your GP"...people really don't like that…they want "should I have a PSA test - or not?"* [P4, G7 Communication Prof Group]<br><br>*"Well it [table of information on Warfarin] says something contrary here...it says 'patients should be told that treatment with Warfarin will mean changes to diet and lifestyle'…* [P2, G9 Dundee Group]<br><br>*"Right well it, it has a contradiction then because it says here…'most patients find Warfarin to be a low burden'… "* [P1, G9 Dundee Group]	
Some people were aware of uncertainty in effectiveness introduced by treatments being tested in limited populations and then prescribed to a wider population. This was a concern for people with multi-morbidity, who were worried about the possible harms of being on multiple treatments.	*"Another aspect of the question is whether the stronger evidence comes from groups that are more vulnerable and not just age related, or whether it's across the board, because if the stronger evidence comes from em people who have underlying conditions that's quite important to know, it becomes a personal choice as it will help us decide, I don't know it's complicated."* [P1, G3 Flu Vac Group]	
The homeless people were shown a chart with a bar representing uncertainty around the expected weight loss from an obesity intervention. Only one of the 4 understood what this bar represented.	*"What do you think the orange bar is trying to tell you?* [interviewer]<br><br>*Good question....Don't know"* [I1, Homeless people interviews]<br><br>*"I think it's … it just gi'es you a variation…about what you...you might be able to lose...they're no' actually saying that you will lose that, because I don't think that would be the right answer anyway, they have got tae dae a variation because of maybe body size or whatever,*	

	you know … but it's good 'cause it gi'es you a rough idea..." [I5, Homeless People Interviews]	
Findings 5.6: Amount and complexity of evidence	Key quotes	Notes on finding
Most people want to see a simple and brief rationale for the recommendation, providing a summary of the evidence but not in great detail. A minority of people want access to a full summary of the evidence underlying a recommendation.	*"Simple, yeah simple…you know straightforward, you know 'this is why we're doing this'"* [P7, G1 Cervical Cancer Group]<br><br>*"...maybe the rationale. You will be given this treatment x and y, to deal with these symptoms. And just to show how it's been collated."* [P1, G6 Young People Group]<br><br>*"...actually explaining the rationale, certainly from, as a sort of non-clinician, non-expert user of the kind of, of this stuff, the explanation of the why the decision is made, having to read through a kind of 40 page appraisal/consultation document to try and find out was it because of a cost-effectiveness, was it 'cause of efficacy, was it 'cause there was no data in the trials really - all they want is a couple of lines…"* [P4, G7 Communication Prof Group]<br><br>*"… they just want to know the answer, and the basic rationale"* [P1, G7 Communication Prof Group]<br><br>*"Yes highlighting what part of, why the decision's made, and that it is made because of these trials to show this, it would just be a really simple way of getting that message out, that we haven't just made this decision up, you know out of malice"* [P7, G7 Communication Prof Group]<br><br>*"...if that could be conveyed in the guideline, so that people could immediately see what was the thing that was likely to change, if the decision, if you want to change the outcome how do you go about it?"* [P10, G7 Communication Prof Group]	Note that all the young people wanted to know about evidence, but not about quality of evidence.

	"Sort of an executive summary really, 3 or 4 paragraphs saying what the position is, what it was based on..." [P4, G7 Communication Prof Group]<br><br>*"…you'd want to know the trials and the studies that had been done, and where, and when [laughs]…eh and get, get some idea of you know some sort of general consensus…you're not, you're not going to go on just one single study that's been done somewhere…"* [P1, G9 Dundee Group]<br><br>*"a' this information … s-sorry … a' this information's been pooled, eh assimilated, the best of which...that is the information that I am interested in…that, the rest o' it, I'm no interested in that's already been decided by the, the clinicians, the professionals. The information that they have decided is..the best that there is..."* [P2, G9 Dundee Group]<br><br>*"You take in the best of it…you're summing it up, that's what you want"* [P2, G9 Dundee Group]<br><br>*"personally I think that would be too much [information on the quality of the evidence]."* [P5, G6 Young People Group]<br><br>*"I think if you were the kind of person who wanted to know the quality then you would do that yourself."* [P4, G6 Young People Group]<br><br>*"...knowing what's behind the guideline would help; I doubt any other members of the public have much idea what they actually base it on."* [P3, G3 Flu Vacc Group]<br><br>*"I think that's the thing, I think some people want a lot of information and they want to know where the information has come from. You know I'd say I was probably at that end of the spectrum but a, a lot of other people are happy [with just the recommendation]"* [P4, G1 Cervical Cancer Group]	

To be meaningful to the patient a guideline has to strike a balance between being too simplistic or being overly complicated and technical. There is a fine line between giving insufficient, and oversimplified, information and overly complex information that people find overwhelming.	*"…it might be counterproductive to put too much...detail on"* [P1, G5 Dundee Group]<br><br>*"...that's what I'm missing, is very often, I mean those [health information] leaflets, you know I've got, I've had a few of those and you read through them and you think well this is so general, it doesn't apply to me, and it's not even interesting to me, you know and then you go to the doctor and you get the high level stuff and you think 'well, I can't understand that', it's the middle level that's missing…" [P3, G5 Dundee Group]*<br><br>*"So a lot of people still want, even within that chronic thing simple answers along the way, and it would make a fully explanatory guide, massive in…hard copy obviously...my question is 'can I get this?', or 'should I get this?', and the more that it's explained in terms of all these different things that are considered the harder it becomes for some people…to manage, so there's just the balance…"* [P6, G7 Communication Prof Group]<br><br>*"...I'm going to have a read of this again [table of evidence about Warfarin], and I'm going to see if I can digest it a wee bit more accurately…this is the type of paper you would need to read, in my opinion, you need to sit down and read it half a dozen times..."* [P2, G9 Dundee Group]<br><br>*"...that's what I mean, for a doctor I think it's not specific, for the patient it's way too specific mmm, you know …so it basically fails on both points"* [P3, G5 Dundee]	Suggested ways to achieve this is layering in an online format and tailoring information to specific audiences e.g. producing multiple versions of guidelines in multiple formats.
The communication professionals want access to the full evidence underlying recommendations. Some participants from other groups also showed an interest in accessing detailed research	*"This is probably entering a world of pain, but would, if NICE mandated, was mandated to publish all submitted evidence, even confidential Pharma evidence, the, I mean Pharma companies are going to have to submit this evidence to get the drugs approved, so would this be a way of getting data that's hidden…"* [P4, G7	Very few people, other then communication professionals were interested in detailed research evidence but people did want to know about a treatments effectiveness in a way they can

evidence.	Communication Prof Group]<br><br>*"… I think that's, it's a really important thing to know what the figures are, what the trials are.."* [P4, G7 Communication Prof Group]<br><br>*"What trials the decision was based on, preferably with links to the Pub Med versions of it..."* [S4, Communication Professionals]<br><br>*"...it depends if you're actually in a situation, and I was recently given some medical news and I then wanted to know absolutely everything, I wanted to read every piece of research, I wanted to look at the impact factor of the journal, how many other people OK had[cited the work, you know because I, it was something really important and it affected me, so I wanted to know"* [P4, G1 Cervical Cancer]	understand.
The amount, and complexity, of information contained in a patient clinical guideline can be overwhelming and lead to information overload.	*"...simplified eh description of the problem....really enabling me to have more clarity em on...first glance...because first glance that's going to put me off"* [P6, G5 Dundee Group]<br><br>*"Way too much…it [a draft patient guideline] needs to be more concise…I personally think that is well over the top"* [I1, Homeless people interviews]<br><br>*"I would simplify…that's an awful lot of information to take in…too much...[it is] information overload [referring to draft patient guideline]"* [I1, Homeless people interviews]<br><br>*"…you see I think...if that was given to a person they would say "OK, I'll read all of it," but they would give up..."* [I1, Homeless people interviews]<br><br>*"Well you've got information overload…straight away..."* [I1, Homeless people interviews]	Evidence presented to patients can be overwhelming. Some groups are unfamiliar even with what evidence is. Others do want a summary of the evidence.

Theme 4.5: Anecdotal evidence

Findings 4.5: Anecdotal evidence	Key quotes	Notes on finding
The influence of personal experience, stories of what happened to family members, friends and celebrities are influential, and can override evidence presented to patients. The influence of anecdotal experience of harms appears particularly strong.	*"…'cause I hear what my sister says and then what..."* [P1, G1 Cervical Cancer Group]<br><br>*"I'm quite interested in the fact that, does it say Warfarin doesn't, doesn't em 'cause any major lifestyle changes…yeah whereas people I know have been on it just hate…you know hate being on it because of the lifestyle implication"* [EJ: G9 Dundee Group]<br><br>*"Because of my diagnosis, my doctor said you have to take it, so I took it and I ended up in hospital, I was very ill....ever since then, I've not taken the flu vaccination.... so em... They had to stabilise that problem, they have to identify what strain, what type of vaccine for flu.....until they know that..."* [P4, G3 Flu Vacc Group]<br><br>*"So do you feel that you could be sent a letter from the GP practice and if you hadn't had that personal experience you would think that it was a good thing for you personally to take the flu vaccination because you've been offered it and it's only because of that bad experience that you would know better? You would knock your faith in their judgement and recommendation."* [P3, G3 Flu Vacc Group]<br><br>*"Our technician took the flu vaccination and was in bed unwell for 4 days whereas I didn't have it and took the flu and was in bed for 5 days. So he was more unwell from taking the vaccine than I was from flu so it's things like that you know if you thought you were likely to be poorly for 3 days"* [P3, G3 Flu Vacc Group]<br><br>*"For somebody who is depressed, I would think myself that CBT would be the last thing I would try. I would first of all sit down with that person and find out what he or she thinks is the problem, he needs listening to, not judging, or not being told pull their socks up..." [P1, G4 Depression Group]*	Note that some are driven to find out all the factual information that they can and access as much hard evidence as possible, but this is a minority.<br><br>Also see harms sub theme and shared decision making finding for other quotes on influence of anecdotal information.

If a person has found an intervention helpful, it is hard to understand why it is not recommended by a guideline.	*"I joined self-help groups, I think they've been helpful to me but there's no research evidence to say how effective they are whereas there's medication and talking therapies which are quite reasonably researched, you can see the evidence for them. Other things can be just quite as helpful...I found self-help groups particularly helpful and you can see the impact they had." [P7, G4 Depression Group]*<br><br>*"I would have thought personally a self-help group would have been quite a good thing to be involved in, simply because … you listen to the other man's story, or, and how does it collate wi' your own? You know and you pick the best bits out" [P1, G8 Dundee Group]*<br><br>*"...art therapy has no evidence. Art therapy for people with depression I can say, in my experience, people have wandered in to our workshop and they are down...I have seen people coming in and I have a chat and I really do praise them, we have lots of paints around, bits and pieces, they don't have to pay for it, but have to keep it clean and it's like whole new people, whole new personalities so yes it's good." [P5, G4 Depression Group]*<br><br>*"I mean it's a very odd list, it doesn't even list the main therapies which are available in the community. It really is quite odd." [P1, G4 Depression Group]*	Seems a particular issue for the depressed group
Any treatment "is worth a shot" irrespective of the evidence base - if you are suffering or at risk of death.	*"I mean we find, we do find that particularly with sort of drugs that cost a lot or treatments that are very long-shot, the cancer, if someone's dying of cancer they will literally do anything, and want to try anything, anything that gives them any hope at all…"* [P8, G7 Communication Professionals]<br><br>*"…even though you go 'well it's like 5%'…they go 'we'll take it!'..."* [P8, G7 Communication Professionals]<br><br>*"Yes everything's worth a shot"* [P4, G8 Dundee Group]	

	"…you know if it's helped like eh, you know if it's helped one person then you know that's a benefit surely" [P5, G8 Dundee Group]<br><br>*"If it's a 50/50 chance and you're desperate, you might go with it…"* [P5, G9 Dundee Group]<br><br>*"…I'll try anything...Oh I'll, I'll try anything…believe me it's…I would…"* [I4, Homeless People Interviews]<br><br>*"I'm a great believer that if you have pain and you feel that this may help you, and you have this let's say "illness" you will try it … you will try it, if you think it will help you. It may not but at least if you think it will you will more than… you'll be happy to go ahead and try it, definitely" [P1, G8 Dundee Group]*	

Theme 4.6: Symbols

Findings 4.6: symbols	Key quotes	Notes on finding
Symbols are most useful if they are understandable from the first glance.	*"... I've seen like the traffic lights scoring which is a lot easier to understand than 1 to 5."* [P4, G6 Young People Group]<br><br>*"…just a logo with a 'thumbs up'… would be helpful [to instantly indicate something that is recommended]"* [P4, G7 Communication Prof Group]<br><br>*"Back to the smiley faces again…because everybody knows [what they mean]...that is an instant…"* [I1, Homeless People Interview]	
Symbols can be very confusing, it may be better to use a text explanation instead of, or alongside symbols, if communicating about evidence.	*"[we gave] sort of star ratings to treatments...we tried to use grade for it, 'cause we have grade with clinical evidence...so if you saw "weak" they would think that it didn't work very well, not that the evidence was 'weak', they'd think 'oh it has a weak effect'…or to give one star for the treatment it meant that one person out of five would be cured or something…we reverted to putting it in the text, so we would write*	This is very similar to what was found when MC tested use of + symbols to indicate strength of evidence

	something about, you know … 'the evidence shows this works for X out of 10 people', or ... 'however, the evidence is not very strong', or 'the evidence is uncertain … we can't rely on the results', something like that to actually explain it in words" [P7, G7 Communication Prof Group]	
Only 1 person understood the use of +/- symbols to represent levels of evidence. Shown to G8 and all the homeless interviewees.	*"What the wee ones wi' the plus in them? ..because what is that for?"* [P10, G8 Dundee Group]<br><br>*"You would...wonder what the heck they were for"* [P6, G8 Dundee Group]<br><br>*"what about the little pluses with the circle round them, the symbol that's a plus and a circle, did you understand what that meant?" [Researcher]*<br><br>*“No” [P4, G8* Dundee Group*]*<br><br>*“No” [P2, G8* Dundee Group*]*<br><br>*“No"* [*P8, G8* Dundee Group]<br><br>*"What about the symbols here; do you understand the purpose of them?” [Researcher]*<br><br>*“Up to four, or, and if you have all four then it is the most beneficial to you…it's basically a star rating, isn't it?"* [I1, Homeless People Interview]<br><br>*"Right, you have a plus sign … if you don’t read that [key for symbols]…what does that mean?" [I1, Homeless People Interview]*<br><br>*"…that, that's not as good as that one I would say because the four pluses are…only in that a plus is a good thing and a minus is not so…"* [I3, Homeless People Interview]	Highlighted in bold, the one person who did understand the use of the symbols to represent strength of evidence.

	"Do you know what this, em these little crosses mean?" [Researcher]<br><br>*"No idea"* [I4, Homeless Interviews]<br><br>*"would you have an idea of what they meant?" [Researcher]*<br><br>*"No"* [I4, Homeless Interviews]<br><br>***"Oh I just about the quality o' the information, isn't it?...how good is it, do you think the evidence...works"*** **[I5, Homeless People Interview]**	

<u>Theme 6: Format</u>

Theme 5.1: Layering

Findings: 5.1 Layering	Key quotes	Notes on finding
There was broad support for layering of information, in a format that allows the reader to access the level of detail that they want.	*"Layering…as well, because some patients that, patients are as different as we are, you know any of us could be a patient, doctors can be patients, patients can be patients, incredibly stupid can be patients, or anyone, you know anyone can be a patient of any sort, so and all sorts of people want different levels of information so it needs to be easy to get the top line and then to drill down when and...and how you want to"* [P8, G7 Communication Prof Group]<br><br>*"...try and provide information at different levels so you can drill down to the level that you're interested in and I think that's really important 'cause patients are interested at very different levels. Some people just want to know the top line, some people want to know 'well why … what's this study based on?', so to give the option of different levels of, I think is useful"* [P7, G7 Communication Professionals Group]	The young people may be particularly keen on information layering. They want to be able to come back and look up more detail at any time. They are keen on the use of technology, such as QR codes/Apps, to facilitate this.

	"…I think coming up with a system of informing people is perhaps going to be difficult because different people want different depths of information. I think the thing is to have a system that people can engage with, if they want more" [P4, G1 Cervical Cancer Group]<br><br>*"some people will look for loads and loads [of evidence]. If it's there then people can choose to read it or feel that they have too many pills or whatever to read about them all and just ignore it but it is good to have it there."* [P3, G6 Young People Group]<br><br>*"you scan it and you can have them come up with a web page on your phone or a picture or an image and it's quite handy. Say for instance you've got your leaflet dot dot dot dot. More information QR code."* [P4, G6 Young People Group]	
A key advantage of online patient guidelines is layered information that enables people to access the exact amount of information required.	*"I'd quite like a guideline that was on the internet and it had, if you wanted more information "click here" right and you could then progress…depending on how much information you wanted"* [P3, G1 Cervical Cancer Group]<br><br>*"That depends on the format you're providing it in…'cause if you just print out a pdf that's slightly more complex than if you can have a Website where you can kind of click and all the stuff happens?... and more stuff appears..."* [P8, G7 Communication Prof Group]<br><br>*"Yeah and it's giving you the chance to go and do more if you want to, which…you know in this day and age is, it's all these apps and what not"* [I3, Homeless People Interviews]<br><br>*"I thought that was quite good, especially when you go to find more information at each [section of a table of information]…"* [P1, G9 Dundee Group]<br><br>*"but the internet is good because then you can decide what information you want, how much."* [P4, G6 Young People Group]	Note that people consistently highlight that although there are benefits of online content many of the more vulnerable groups, such as older people, do not have access to online information.

	"… 'cause if you just print out a pdf that's slightly more complex than if you can have a Website where you can kind of click and all the stuff happens...and more stuff appears...[P8, G7 Communication Professionals]<br><br>*"…you know eyes, point it at, how often should I have an eye test, or whatever, and then as Susan says you can click through different levels…*[P4, G1 Cervical Cancer Group]<br><br>*"...you know you said different stories, was that something that you would click on, or was that just something that was going to be part of a publication?...Yeah it looks like IT, if you're wanting to have eh stories in the background it has to be doesn't it"* [P1, G5 Dundee Group]	
The surface level of information should be kept as simple as possible, with the option to access more complex information.	*"If people don't want to know they won't look, but if people do they will dig deeper. We need basic information and it's down to individuals and if people want to dig deeper they will. If they don't want to bother, they won't bother"* [P2, G2 Diabetes Group]<br><br>*"yeah you could have bullet points and then look them up when you have more time."* [P1, G6 Young People Group]	
Layering was also highlighted as a format that facilitates access to individualised information.	*"...if you have it set up, the guidelines structured in a way where you can go deeper to find things which are pertinent to you as a, an individual well that's the way it should be" [NR, G5 Dundee Group]*	Not discussed directly by anyone else, but fits with concerns of groups about contraindications, need for taking account of individual difference like genetic family history in making treatment choices.

Theme 5.2: Tables and text

Findings 5.2: Table and text	Key quotes	Findings: Table and text
There was broad support for any form of chunking text, in particular bullet points and tables	*"For me, I'm quite a logical thinker so I would like bullet points..."* [P1, G3 Flu Vacc]	

were liked for their ability to convey information quickly and clearly.	*"I think you just want to give the patient information in bullets." [P2, G4 Depression Group]*<br><br>*"...I like the idea of the...eh bullet points...'cause that, again that simplifies, it highlights…" [I4, Homeless People Interviews]*<br><br>*"Yeah the layout's good though, the bullet points" [P5, G8 Dundee Group]*<br><br>*"It's clear and concise [m-mmm] and also the number of bullet points are only seven…" [I4, Homeless Person Interview]*<br><br>*"I think it's very clear, I mean it gives you a, a table there, you know and a key and everything, it's telling you what to do in, in boxes — I'm a great believer in boxes…" [I3, Homeless People Interviews]*<br><br>*"it [table of information on Warfarin] gives eh an indication of what would work, and what may not work, it gives that indication right away. It's very brief...I found it helpful" [NR, G5 Dundee Group]*<br><br>*"They're [tables] instantaneous as well…look at them you can understand them…" [P1, G5 Dundee Group]*<br><br>*"…tables and stuff like that in the midst o' text are very attractive, to me, when I open, when I turn a page, even though the table or the graph on, two pages on is not relevant to what I'm reading at the moment, but it's something relevant half a page on, you turn the page, you, your eyes automatically it goes to that..."* [P1, G5 Dundee Group]	
Information overload: Long, unbroken sections of text are less likely to be read. Short and simple text explanations were the preferred format.	*"Again, a quick read at that, I mean you're not, you're, for a start you're not digesting everything it's trying to tell you, at the start, you know you're, you're skipping over it"* [P1, G5 Dundee Group]<br><br>*"not too many words close together. People will look at it and think 'oh*	

	I don't want to read that' like seeing a massive text and it's too much." [B, Young People Group]<br><br>*"You don't want to crowd it...too much reading in it…"* [P10, G8 Dundee Group]<br><br>*"...sometimes if, if, you know the reading can be quite difficult…"* [P1, G9 Dundee Group]<br><br>*"Slightly too much information…but that seems to me to be, it [draft patient clinical guideline] could be more concise…"* [I1, Homeless People Interviews]<br><br>*"Well you've got information overload…straight away; there's, there's too much writing … and in fact … yeah, that's all the way through … why not … more graphic as opposed to so much text … pictures, smiley faces..."* [I1, Homeless People Interviews]<br><br>*"I mean again if you read it [section from draft patient guideline]...but you might not, you might look at it and think 'nah'"* [I3, Homeless People Interviews]<br><br>*"I think some of it's a bit long-winded…it could be shortened..."* [I4, Homeless People Interviews]	
Information produced as a hard copy should be kept short, and focused, to appeal to the majority of readers. However as noted elsewhere this is balanced by the need to provide detailed information. Hard copies may need to be produced in multiple formats to appeal to different groups.	*"this booklet [SIGN patient guideline]...patient themselves cannot all read it and therefore you have to produce something shorter and simpler."* [P2, G4 Depression Group]<br><br>*"I've got a positive suggestion, a slimmed down version of this [patient version of SIGN guideline], there's a 4 paged leaflet on depression and it says, are you depressed? How to find help, so something really basic, I mean this is quite advanced and we want something like that as well"* [P1, G4 Depression Group]<br><br>*"They [SIGN]...they've got a sort of shortened version which actually*	See other themes for more supporting evidence.

	works very nicely" [P7, G7 Communication Prof Group]	
Information presented in a table is given the weight of fact or evidence.	*"...a decision you've to make or a thing you go to the doctor about and that's given you a table of projected success or otherwise, it is invaluable … tables are good. 'Cause that's, that's the factual stuff...that it's getting factual stuff in tables and tables are evidence...aren't they?" [SB, G5 Dundee Group]*<br><br>*"Tables are extracted from fact, so tables…"* [SB, G5 Dundee Group]	
Ensure there are clear headings and sections on each page of a patient guideline, to flag different topic areas.	*"There seems to be set paragraphs probably for different sources of information, or a new topic [that is]...helpful"* [P5, G8 Dundee Group]<br><br>*"The heading stands out..."* [P2, G8 Dundee Group]	
Too much dense text makes it very hard to locate the information that you want to extract from a clinical guideline.	*"I was looking for the information about drugs and stuff and actually even when I got to the page saying Sutent's appraised for kidney cancer the very, the line I wanted to read on that page was "Sutent has, or hasn't been approved for kidney cancer", and it wasn't there, there was a pdf which I had to download, open up, skim through the opening of Executive Summary..."* [P4, G7 Communication Prof Group]<br><br>*"...you have to go to the full several hundred pages document, and neither that nor the intermediate one, which is aimed at healthcare professionals, nor the patient's smallest version, they don't, you can't cross-reference easily, section numbers don't tally…"* [P2, G7 Communication Prof Group]	
People lacking in health literacy were identified as a group that was more vulnerable because of their difficulties with comprehending the type of information found in clinical guidelines.	*"... you know we're medical literate, we understand what constitutes good research evidence, maybe what doesn't…so maybe we're in a slightly different category"* [P3, G1 Cervical Cancer]<br><br>*"you can reduce the reading age of something quite considerably and it's much more helpful...I think some adults may not have gone beyond that stage at school [10-11yrs]..."* (P1, G9 Dundee Group]	

	"...what would happen if somebody's dyslexic…" [I1, Homeless People Interviews]<br><br>*"...there was a lot of medical jargon...I know from experience and you know congratulations for producing this booklet [SIGN patient guideline] but patient themselves cannot all read it...[P2, G4 Depression Group]*<br><br>*"There was a recent article referring to medical illiteracy and its if you go away with paperwork or like inside your medicines packet there is information, people who are unable to absorb that information diminish their lifespan by 5 years research...*[P1, G3 Flu Vacc Group]	

Theme 5.3: Images and colour

Findings 5.3: Images and colour	Key quotes	Notes on finding
Images are helpful if they convey meaning, an image can make the topic that a section of text is focused on obvious from the first glance.	*"so what we need is it'll be some, some sort of pictorial representation, some sort of eh…really enabling me to have more clarity em on a, a first glance kind of type of looking at it, you know, em because first glance that's going to put me off [table of information on Warfarin]"* [P6, G5 Dundee Group]<br><br>*"...like a caricature, like sort of pointing to the throat or something like a, a man with a…you know so we know what we're talking about…"* [P6, G5 Dundee Group]<br><br>*"it [patient information leaflet] explained everything about the stroke to you...and you turned the page and, and just read it and it had pictures and, it was quite… it was explicit, you could…read it and understand it…"* [P2, G9 Dundee Group]	
Images will draw the eye to that section of a page, and break up the text.	*"…it's like if you open up a newspaper and it's, two pictures, I mean to me the first thing you do, you look at the pictures…"* [P1, G5 Dundee Group]<br><br>*"...you've got information overload…straight away; there's, there's too*	

	much writing … and in fact … yeah, that's all the way through … why not … more graphic as opposed to so much text … pictures, smiley faces...you know something" [I1, Homeless People Interviews]<br><br>*"I would probably give it a wee bit of colour if possible, but I mean again it is just really a, a statement isn't it, so, but yeah maybe a bit of colour just to catch the eye"* [I3, Homeless People Interviews]<br><br>*"I don't think, it's not necessarily that there's too much text, that's not a problem for me, eh but also some sort of pictorial representation would be e-h very conducive" [SB, G5 Dundee Group]*	
The use of images can make a guideline appear more 'personal' and can help to humanise the information being conveyed.	*"that [images] makes it more personal"* [B, Young People Group]	
Inappropriate or poor quality images may make a document less attractive and give it a 'cheap' appearance.	*"...sometimes when you see things like that [draft patient guideline] in the stand at the doctor you think well has somebody just typed it up in their break?...I was going to say pictures [would make it look more professional], but pictures might cheapen it"* [P10, G8 Dundee Group]	
The use of colour and images makes a guideline more attractive and therefore more likely to be read.	*"…more colours, em, I don't think, it's not necessarily that there's too much text...but also some sort of pictorial representation would be e-h very conducive"* [P6, G5 Dundee Group]<br><br>*"It's more attractive with colour…but I don't know if it's easier to read"* [P10, G8 Dundee Group]<br><br>*"I've got the black and white one but I think the colour one's good...colour's good isn't it?" [P10, G8 Dundee Group]*<br><br>*"you know boosted with diagrams and visuals…"* [P1, G9 Dundee Group]	
Work with peoples associations with colour, for example red and danger. Use of multiple colours in a chart can be confusing: keep it	*"…be careful with the colours, like amber green red..."* [P11, G7 Communication Prof Group]<br><br>*"...I would rather just see words…like 'so and so per cent did this, and*	People found the prostate screening chart confusing - colour use was unclear and numbers too high. However the concept of

simple.	*so and so per cent did that'…than, than all these colours [in a chart of outcomes from prostate screening]" [P5, G9 Dundee Group]*	false positives in a screening test was also something new to the group and made it more confusing. People found use of grey and white in a chart very unhelpful.

Theme 5.4 Format
The availability of a variety of formats, such as print, online materials, CD, and APPs, increases accessibility and enables dissemination to the widest possible audience.

Findings: 5.4 Format	Key quotes	Notes
Multiple formats are necessary if patient guidelines are to be accessed by the widest possible audience.	*"I think they should be in a variety of format, apps, internet so that anyone can source them, I think it's useful to have access to this information"* [P2, G4 Depression Group]<br><br>*"guidelines have got to go on the Internet, you can't have anything else nowadays because we have a whole, a whole generation who do nothing else but look at the Internet"* [P1, G5 Dundee group]<br><br>*"Researcher: so if it was on the internet would you be more likely to print it out, and then read, read it?*<br>*...Well I would, 'cause I like time to take it all in…at my age group"* [P1, G1 Cervical Cancer Group]<br><br>*"The web address for the guidelines would be helpful so people can get hold of copies, say this is the website if you want to get hold of a copy, here's the address..."* [P2, G2 Diabetes Group]<br><br>*"...that [referring to a patient version of a clinical guideline] is in quite an accessible format it's quite good for maybe people who are at university or have a decent level of education, so I think it's not going to be one answer for all" [P7, G4 Depression Group]*	Many people (older people in particular) want a print copy. If they can access something online they will generally print a hard copy. Particularly printed text versions of guidelines may require a relatively high level of education to process. It may be helpful to provide a range of levels of complexity of written materials. The suggestion that current patient guidelines may be too complex for some patients currently is supported by the feedback regarding information overload from text based guidelines.
There was support for newer	*"yeah apps are the way forward." [P2, G6 Young People]*	The use of prominent QR codes in

technology like APPs and QR codes, particularly from younger people. However all age groups recognised that only a minority of people can currently access patient clinical guideline this way. They can only supplement other formats.	*"...QR codes work well...you scan it and you can have them come up with a web page on your phone or a picture or an image and it's quite handy. Say for instance you've got your leaflet dot dot dot dot. More information QR code" [D: G6 Young People]*<br><br>*"yeah cos not everyone has an app on their phone. I think a lot more people would use the internet."* [P1, G6 Young People]<br><br>*"...my son in-law has got one of those phones, he does all this stuff, I feel exhausted thinking about how to work it."* [P3, G3 Flu vaccination group]<br><br>*"If you're depressed m-mmm and you had a Smart Phone, one, would you know how to use it? I've got a Smart Phone...I don't know how to..."* [I1, Homeless person]	the mocked up materials for user testing was very off putting to some people. The presence of them made them feel that the document was not for them and they were distracting.
Formats such as CDs and videos may be particularly important in reaching vulnerable and disadvantaged groups.	*"...a CD format ... that would, 'cause then you, you know could put it on and, and you can put it on again...and I always find when I'm, when I'm listening to something it tends to …and it, it sort of stays there. I think very often with sort of reading … you know it's, it's very…I mean I find it hard to take in information"* [I4, Homeless person]<br><br>*"...they had a video in the hospital, and they let us, made us sit in the living room and watch this, you know in this private room …yeah and we think it's a good idea before you go home [following a stroke]…*[P4, G9 Dundee Group]	NB: these formats have only been mentioned by these two people but are highly praised and the issue of difficulties with text and internet formats is mentioned frequently. As is the problem that many text versions are too demanding for some patients.
Vulnerable groups may benefit the most from a patient clinical guideline being available in multiple formats	*"...I find actually listening to something, so maybe a CD format ... that would, 'cause then you, you know could put it on and, and you can put it on again,...and I always find when I'm, when I'm listening...you end up learning the words…and it, it sort of stays there....reading …I mean I find it hard to take in information"* [I4, Homeless People Interviews]	Diabetes group G2 highlighted need for a large print format for people with poor eye sight at the end of the group after recorder turned off.<br><br>While not many people explicitly make the link with other formats, many raise concerns about

		vulnerable groups like elderly people, dyslexic people, and depressed people, those with limited literacy and how they will access online material or read relatively long and complex patient clinical guidelines.

Other themes

Theme: Voluntary Groups

Findings 3.3: Voluntary Groups	Key quotes	Notes on finding
Contact with patient groups gives patients access to people that have had the same experiences as they have - this is encouraging and highlights different options.	*"Yeah it's good to know that other people have been through certain situations the same as you... personal experiences...that kind of thing sort o' encourage you and it helps you to decide what options you had"* [P7, G1 Cervical Cancer]<br><br>*"...voluntary groups are important...people there have been there, done that and got the t-shirt, you can use it the way you want and you are talking to people who are in the same boat as you..."* [P2, G2 Diabetes Group]	
Patient groups are a good means of accessing the views of patients.	*"...often clinicians might measure outcomes but then the, the things that they think are important are not the things that the patients think are important are actually suffering for this disease that's why I think it's important that they do go to Patient Groups when they're doing their guidelines m-mmm, because patients will soon tell you, you know what matters to them about coping with their condition"* [P3, G1 Cervical Cancer]	
Patients with conditions that do not have a dedicated charity may be disadvantaged in terms of	*"...I think they'd probably come to Diabetes UK and get our "What Care To Expect" document, which just outlines the sort of minimum*	

accessing health information.	*things that you would expect from your doctor...but I'm just thinking of course there must be conditions and guidelines for conditions which doesn't have...a dedicated, you know charity…"* [P3, G7 Health Professionals]	
Although voluntary organisations are excellent sources of health information (see theme 1), they have organisational objectives which may lead to disagreement with guideline recommendations therefore information produced by them cannot entirely replace patient versions of clinical guidelines.	*"charities are producing the same information, sometimes maybe there'd be duplication, but we don't always blindly go along with the guidelines, we may fundamentally disagree with it, so they need to have a simple version that someone can see and we need to have simple information on how we comment on that"* [P2, G7 Health Professionals]<br><br>*"...charities have agendas and getting your, you know your advice on Abiraterone from our Website will give you a very different view from NICE's Website simply 'cause we're jumping up and down...trying to get it approved"* [P4, G7 Health Professionals]	
