# Supplementary material for: What do patients and the public know about clinical practice guidelines and what do they want from them? A qualitative study
Source: BMC Health Serv Res. 2016 Feb 24;16:74. doi: 10.1186/s12913-016-1319-4 (PMC4847193; doi:10.1186/s12913-016-1319-4)
Supplement: Additional file 3: — Depression focus group mock guideline materials. The mock guideline materials shown to the focus group with people with depression (PDF 134 kb) [file 12913_2016_1319_MOESM3_ESM.pdf]

My doctor says I have depression. What can I do to help myself feel better?

Some background

The advice in this document comes from a clinical **guideline.** Guidelines are produced to advise doctors and their patients what treatments are likely to work best for them. (We will use 'doctor' for simplicity, but this applies equally to other healthcare professionals such as nurses, therapists, and so on.)

Guidelines are not rules. Treatments recommended in guidelines are those likely to work for most people, most of the time. Everyone is different and it is possible that some recommended treatments are not the best ones for you. Your doctor knows your individual circumstances and is the best person to discuss this with you.

Recommendations in this guideline are based on evidence. Evidence comes from experiments that are generally referred to as clinical **trials.** Trials do not give exact right or wrong answers, but give us an **estimate** of how likely it is that a treatment will work. Many factors can influence the reliability of this estimate, so in this document we will indicate how sure we are of the estimate by linking it to a number (up to 4) of these symbols ⊕. The more symbols, the more reliable the estimate and the more likely you are to get the expected benefit if you adhere to the treatment.

What are the best ways to help myself feel better?

There is good evidence that using Cognitive Behaviour Therapy (CBT) can improve how you feel. ⊕⊕⊕⊕ This is a therapy that helps you to change your thoughts and behaviour. Evidence has shown that using this technique reduces the likelihood of having a major depressive illness by around one third compared to normal discussions with your GP.

This is probably the most effective treatment, and if your GP agrees that it could work for you there are three options you should consider. These are (i) seeing a therapist for a weekly session for around six weeks; (ii) accessing CBT through a computer program, or (iii) your GP can recommended guided CBT books. The option that you have will depend on your discussions with your GP and what is available in your area.

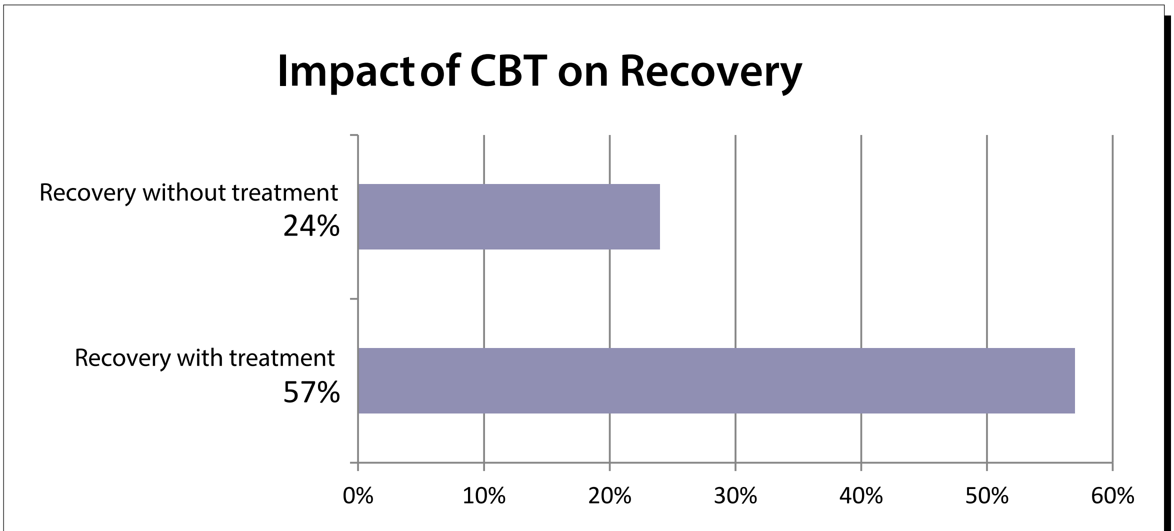

Percentage of people responding to treatment vs control group response with no treatment

Computerised CBT

Computerised CBT has been shown to substantially reduce depression and improve mood, as well as being acceptable to the majority of patients ⊕⊕⊕. It has also been shown to be a cost effective approach. (In other words the benefits to patients outweigh the cost of delivering the therapy). Having someone to support you while using a self-help computer program may be helpful. These programmes can help you to understand depression and develop the skills you need to deal with your problems.

Self-help

There is no evidence to tell us how useful **self-help support groups** are as a treatment for people with depression. This does not mean that there has not been success for some people, just that there is not enough research evidence to demonstrate its success. You may feel that it would be helpful to speak to people in similar situations who will know what you are going through. You may also find that combining some kind of informal support along with use of CBT works better than either on its own.

If this is not successful for you then your doctor will be able to discuss other treatments with you.

If you are comfortable about sharing experiences in a self-help group, you can try that in association with other therapies aimed at curing your depression.

I have heard that exercise can help cure depression. Should I give it a try?

Research has shown that **structured exercise programmes** can help improve symptoms of depression and lift your mood at least as effectively as drugs or CBT. By exercising you can build your self-confidence, meet other people and improve your mental health at the same time. **If you feel you would like to be more active, a structured exercise programme can be helpful in addressing depressive symptoms.**

Choose a type of exercise that you enjoy, as you are more likely to remain motivated to continue with the programme. If you have not exercised regularly for some time, consult your GP before starting a programme. A fitness instructor should be able to advise on the best form of exercise for you.

Will changing my diet or lifestyle help?

There is no good evidence that making changes to your diet or lifestyle will affect your mood or depression. There is much to be gained, however, through following general advice on factors such as:

- alcohol and drug use
- diet and eating behaviours
- maintenance of social networks and personally meaningful activities
- sleep problems.

I am interested in nutritional supplements and herbal remedies. Are any of them likely to be of help?

There is no evidence that the majority of herbal remedies or nutritional supplements make a difference to clinical depression. The sole exception is St. John's Wort where there is some evidence that it can work, but no better than standard drugs prescribed for depression. There is also evidence, however, that *Hypericum* (the plant from which St. John's Wort is derived) interacts with various prescription drugs - not just those prescribed for depression - with potentially serious consequences. This means that you should not take St John's Wort unless you have specifically discussed this with your doctor.

I am interested in complementary therapies. Are any of them likely to be of help?

Complementary therapies include a wide range of interventions in such as acupuncture, massage therapy, and yoga. There is no evidence that any of these therapies make a difference to depression. You should note that this lack of evidence does not mean these therapies do not work, just that there is no evidence that they do work. There is probably very little risk of harm if you try any of these therapies and they make you feel better. Just bear in mind that they should be used in conjunction with other tested therapies that have been shown to make a difference to your underlying state of health.
