# Supplementary material for: What do patients and the public know about clinical practice guidelines and what do they want from them? A qualitative study
Source: BMC Health Serv Res. 2016 Feb 24;16:74. doi: 10.1186/s12913-016-1319-4 (PMC4847193; doi:10.1186/s12913-016-1319-4)
Supplement: Additional file 4: — Healthcare scenario workbook. Healthcare scenario workbook used for interviews with people experiencing homelessness. (PDF 958 kb) [file 12913_2016_1319_MOESM4_ESM.pdf]

DECIDE

Developing and Evaluating Communication strategies to support Informed Decisions and practice based on Evidence

GRADE

Healthcare scenario:
Using a guideline on weight management

For User-feedback DECIDE WP3

January 2013

Instructions for discussion and feedback

- If you are paired with a colleague, please discuss the questions together and fill out one form.
- If you disagree or have a lot of discussion, please indicate this, reporting of your answers and your points of discussion.
- If you need more space, please use the back of the paper (label extra text with appropriate numbering).
- If you want to use extra paper, feel free! You can also make sketches if you have ideas of better ways of presenting the information. **It would be really appreciated.**

Healthcare scenario:
Using a guideline on weight management

- Imagine that you or a member of your family is worried about being overweight.
- You want to find out more about what sort of things might help a person to lose weight.
- At the moment, you or your family member does not want to discuss this with a GP or other health professional.
- You decide to look for more information yourself.

Question 1

Where would you look for information?

(please discuss…)

Answer:

Question 2

If you were worried about your weight, what information would you be looking for? What information would help you?

(Please discuss....)

Answer:

Instruction before 1st example page: First impressions

- On the next page (don't look yet!) you will find a list of contents for a booklet on dealing with obesity. We'd like you to imagine that you've found this while searching for information.
- When you look at the next page, we want your **first immediate spontaneous impression**. Don't think, just tell me the first thing that comes into your head when you see it. (Now you can look…)

Example page 1

SIGN
Scottish Intercollegiate Guidelines Network
Part of NHS Quality Improvement Scotland

NHS
Quality Improvement Scotland

Management of obesity in adults

This document includes information on:

1. How guidelines are produced
2. Key messages about managing obesity
3. What is obesity?
4. What can you do
5. Things your doctor will suggest
6. Things you and your doctor need to talk about
7. Where can you go for help and support

Question 3

What are your first spontaneous reactions?

Answer:

Question 4

Is there anything from your own list (your answer to Question 2) that you would add to the list in the example?

Answer:

Instruction before 2nd example page:

- One of the first pages in the booklet explains what a guideline is. The next page gives the text from the booklet.
- We would like **you to read the page** and then answer a few questions.

DECIDE
GRADE

Example page 2

How guidelines are produced

Guidelines help healthcare professionals and others decide about the best treatment and care for people with a particular condition. The aim of guidelines is to improve care, not to reduce costs. Although costs may be reduced, following a guideline could also increase costs by, for example, recommending more tests, longer hospital stays, or more specialist care. Guidelines are generally developed following a set method and use the best research evidence available. They are developed by an expert group (the guideline development group) that usually contains clinical experts, researchers, patients and carers. The topic of a guideline is chosen in several ways: the condition may be common, or put a high burden on people with it; new research may have become available and an existing guideline needs to be updated; there may be a lot of uncertainty or variation in clinical practice and a guideline could help reduce this; or health professionals, the public or others may suggest the need for a guideline. Other reasons are possible.

Producing a guideline can take a long time because of the quantity of research, or because the research is hard to interpret. Guidelines also need to take on board a lot of views, and that takes time. If there is not enough evidence from clinical research, the advice is based on the views of members of the group developing the clinical guideline and other experts. In some cases, the guideline development group may decide that there is not enough evidence to recommend whether a test or treatment is useful or not.

Guidelines are produced in a range of formats including paper, PDFs, electronic tools, and apps for smartphones. They are also sometimes tailored for different audiences so that, for example, there is a version for doctors and another version for the public. The research supporting these versions is the same but the presentation is different and they may choose to emphasise different aspects of the research. Versions for the public might concentrate on self-management for example.

Finally, guidelines are not rigid instruction books. The ultimate decision about a particular clinical procedure or treatment will always depend on each individual patient’s condition, circumstances and wishes, and the clinical judgement of the healthcare team.

Why now?

We update guidelines roughly every three years, or when significant research evidence becomes available. This November 2012 guideline is a routine update of an guideline published in August 2009.

Question 5

a. What do you think about this information? Is it enough/too much? Does it help you understand what a guideline is?

b. Is this information useful?

If you want to rewrite any part of this text, feel free to make a suggestion. Use a separate piece of paper.

Answer (a):

Answer (b):

Instruction before 3rd example page:

- The booklet also aims to explains what a person can do to manage his or her own weight. On the next page (don't look yet!) is a copy of a table from the booklet.
- When you look at the next page, we want your **first immediate spontaneous impression**. Don't think, just tell your colleague the first thing that comes into your head when you see it. (Now you can look…)

Example page 3

Preventing obesity		
**What you can do yourself**	**How good is the evidence that this works?**	**Where you can go for more help**
• Eat fewer energy-dense foods like high fat food, sugary drinks, chocolates and sweets and eat low energy-dense foods like cereals, fruit and vegetables instead.<br>• Eat less fast food and drink less alcohol.	○⊕⊕⊕	Have a look at http://www.nhs.uk/livewell/healthy-eating/Pages/Healthyeating.aspx
• Be more physically active and reduce things like watching TV.	○⊕⊕⊕	Being active doesn't mean you need to be sporty, you could simply walk a little more (try getting off the bus a stop early, or walking rather than driving to the shops). Have a look at http://www.nhs.uk/livewell/fitness/Pages/Fitnesshome.aspx for more ideas.
• Weigh yourself regularly.	○⊕⊕⊕	All you need is a set of scales.
Changing your behaviour		
• To manage obesity effectively, it's best to get professional advice on how to change your behaviour.	⊕⊕⊕⊕	Start by talking to your GP

Key: ⊕⊕⊕⊕ = High quality; ○○○⊕ = Very low quality

Question 6

What are your first spontaneous reactions?

Answer:

DECIDE GRADE

Question 7

a. Is this information understandable and helpful?

b. What do you understand from the circles with crosses in them?

c. Is the 'Where to go for more help' necessary/useful?

d. Anything you would add, remove or change?

(please discuss…)

Answer (a):

Answer (b):

Answer (c):

Answer (d):

Instruction before Example page 4:

- The booklet also aims to tell you something about the size of benefit that particular things (eg. reducing the amount you eat) will have on weight.
- We're going to show you an image (don't look yet!). We'll also hand you them as separate sheets because will ask you to compare several next to each other a little bit later.
- We want your **first immediate spontaneous impression** of the image on the next page. Don't think, just tell your colleague the first thing that comes into your head when you see it. (Now you can look….)

Example page 4: Version A

Program: *Reduce energy intake*

Outcome: *Change in weight*

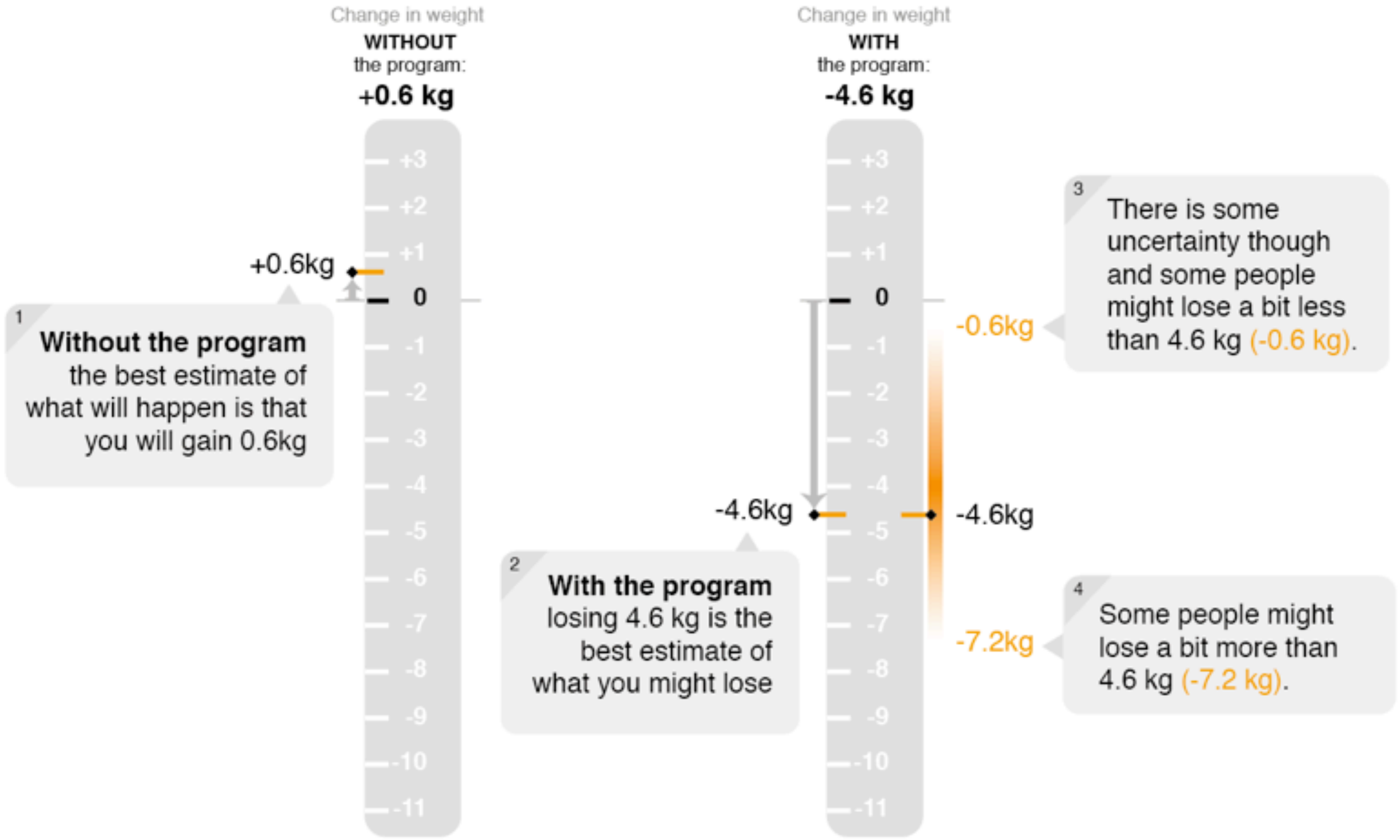

Question 8

What are your first spontaneous reactions?

Answer:

DECIDE

GRADE

Question 9

a. Does it contain enough information for you to understand what is likely to happen with and without the program?

b. If you wanted to know how much weight you would expect to lose, would this sort of image be useful? Is anything missing?

Answer (a):

Answer (b):

Instruction before Example page 5 and 6:

- We have a few versions of this image, each slightly different. The differences are small but potentially important.
- We're going to show you two more images (don't look yet!), which we'll also give you on separate sheets so you can put all of them next to each other.
- We'll tell you what has been changed and we'd like to know what you think. (Now you can look….)

Example page 5: Version B

Changes: we've removed the orange '–0.6kg' and '–7.2kg' by the orange bar.

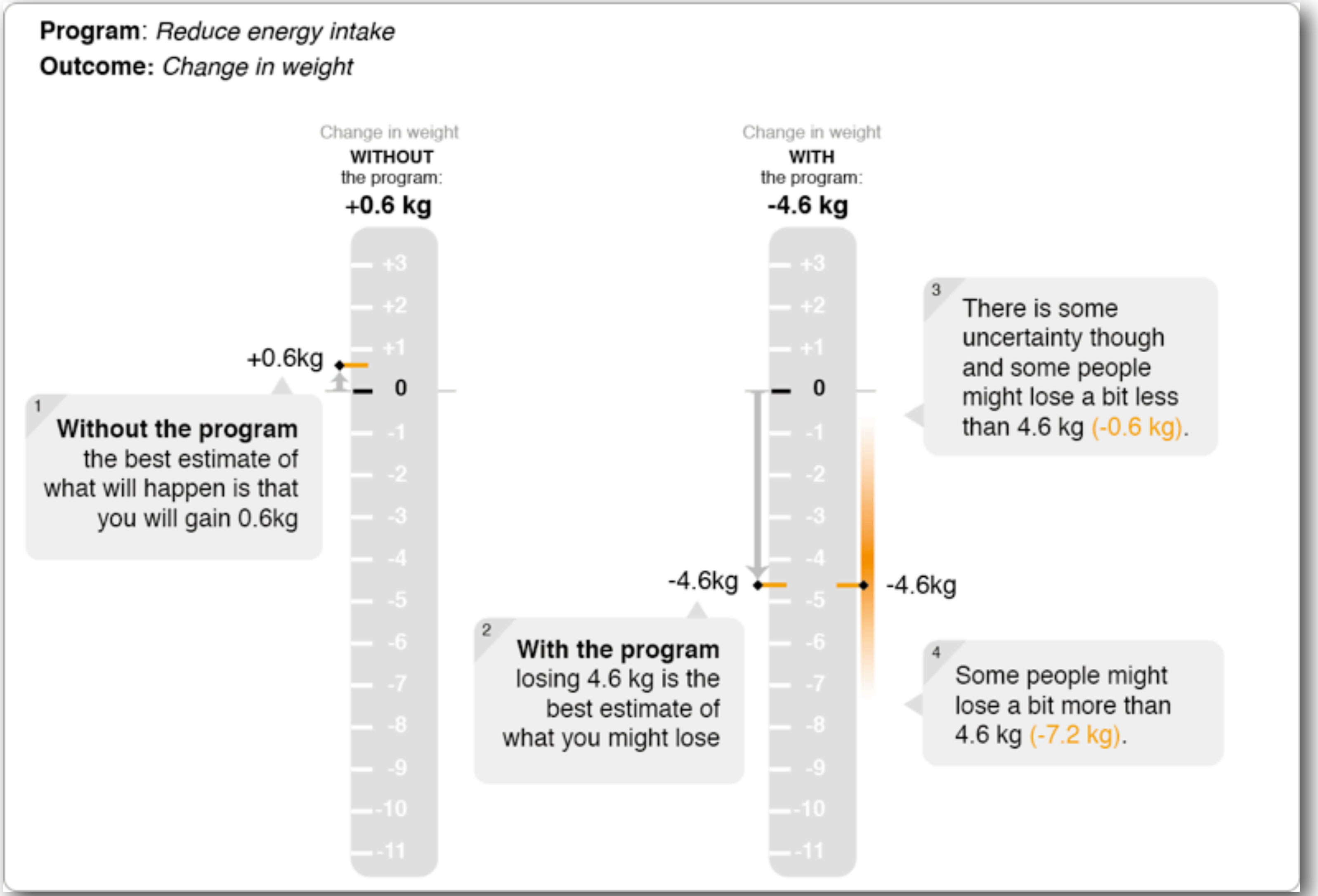

Example page 6: Version C

Changes: we've removed all the orange numbers.

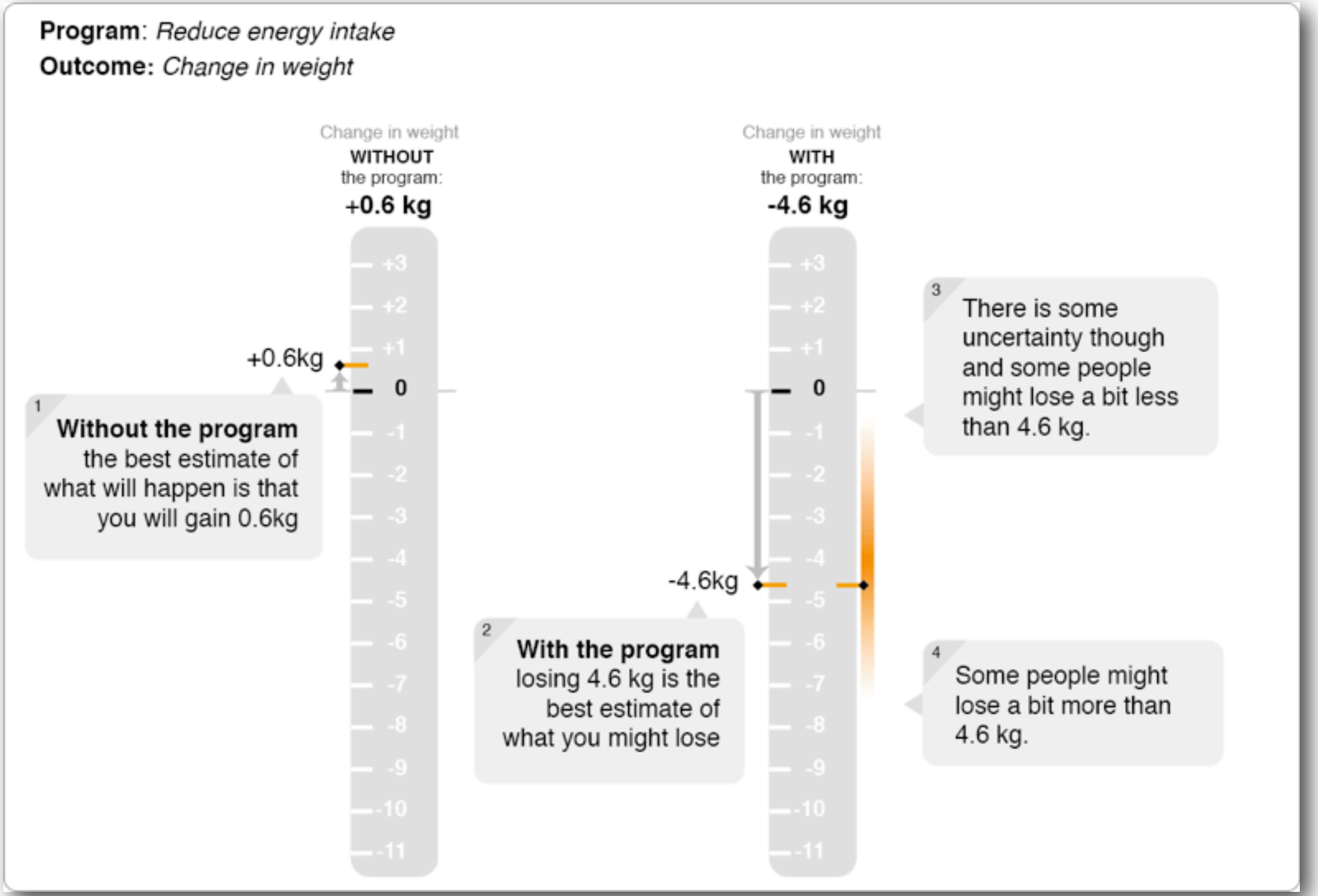

Question 10

a. Looking at all three images, do our small changes make any difference to how easy they are to understand?

b. What do you think the orange bar at the right is trying to tell you? What do you think the shading means?

c. Which of the images (A–C) works best for you?

d. Anything you would add, remove or change?

(please discuss…)

Answer (a):

Answer (b):

Answer (c):

Answer (d):

[Blank sheet for drawing any alternatives/ideas you have]

Thanks very much for your feedback – the last question is below! If you have any other comments, please write them in the box to the right.

Do you think this a good way of collecting user feedback:	Other comments about the information we've been showing you:

DECIDE
GRADE
